# Mining Featured Biomarkers Linked with Epithelial Ovarian CancerBased on Bioinformatics

**DOI:** 10.3390/diagnostics9020039

**Published:** 2019-04-09

**Authors:** Varun Chandra Alur, Varshita Raju, Basavaraj Vastrad, Chanabasayya Vastrad

**Affiliations:** 1 Department of Endocrinology, J.J. M Medical College, Davanagere, Karnataka 577004, India; varunalur@gmail.com; 2 Department of Obstetrics and Gynecology, J.J. M Medical College, Davanagere, Karnataka 577004, India; varshitha.r.gowda@gmail.com; 3 Department of Pharmaceutics, SET’S College of Pharmacy, Dharwad, Karnataka 580002, India; basavarajmv@gmail.com; 4 Biostatistics and Bioinformatics, Chanabasava Nilaya, Bharthinagar, Dharwad, Karanataka 580001, India

**Keywords:** epithelial ovarian cancer, bioinformatics analysis, differentially-expressed genes, PPI network, survival analysis

## Abstract

Epithelial ovarian cancer (EOC) is the18th most common cancer worldwide and the 8th most common in women. The aim of this study was to diagnose the potential importance of, as well as novel genes linked with, EOC and to provide valid biological information for further research. The gene expression profiles of E-MTAB-3706 which contained four high-grade ovarian epithelial cancer samples, four normal fallopian tube samples and four normal ovarian epithelium samples were downloaded from the ArrayExpress database. Pathway enrichment and Gene Ontology (GO) enrichment analysis of differentially expressed genes (DEGs) were performed, and protein-protein interaction (PPI) network, microRNA-target gene regulatory network and TFs (transcription factors) -target gene regulatory network for up- and down-regulated were analyzed using Cytoscape. In total, 552 DEGs were found, including 276 up-regulated and 276 down-regulated DEGs. Pathway enrichment analysis demonstrated that most DEGs were significantly enriched in chemical carcinogenesis, urea cycle, cell adhesion molecules and creatine biosynthesis. GO enrichment analysis showed that most DEGs were significantly enriched in translation, nucleosome, extracellular matrix organization and extracellular matrix. From protein-protein interaction network (PPI) analysis, modules, microRNA-target gene regulatory network and TFs-target gene regulatory network for up- and down-regulated, and the top hub genes such as E2F4, SRPK2, A2M, CDH1, MAP1LC3A, UCHL1, HLA-C (major histocompatibility complex, class I, C), VAT1, ECM1 and SNRPN (small nuclear ribonucleoprotein polypeptide N) were associated in pathogenesis of EOC. The high expression levels of the hub genes such as CEBPD (CCAAT enhancer binding protein delta) and MID2 in stages 3 and 4 were validated in the TCGA (The Cancer Genome Atlas) database. CEBPD andMID2 were associated with the worst overall survival rates in EOC. In conclusion, the current study diagnosed DEGs between normal and EOC samples, which could improve our understanding of the molecular mechanisms in the progression of EOC. These new key biomarkers might be used as therapeutic targets for EOC.

## 1. Introduction

Epithelial ovarian cancer (EOC) is one of the most common gynecological malignancies. At present, the therapy of ovarian cancer patients depends broadly on pathologic staging, while pathological biopsies still play a crucial role during diagnosis. The majority of patients with EOC exhibit a poor prognosis, and the disease is characterized by a high mortality rate [1]. Although various breakthroughs in this field allow for the clinical management of EOC, its underlying molecular mechanisms remain poorly understood. The mortality caused by EOC has lowered dramatically in the last decade due to great developments in early diagnosis and treatment [2]. The essential clinical diversity is most likely due to the genetic heterogeneity of each EOC patient [3]. Therefore, determining the diversity in the genetic profile of EOC that controls the prognosis, as well as specific risk evaluation based on genetic screening, would lead to novel and more powerful clinical strategies for treatment.

A large number of reports have been published to explore the molecular mechanism of EOC advancement. It has been shown that stimulation of oncogenes and inactivation of tumor suppressor genes are important for the induction and development of EOC [4,5,6].Nakayama et al. [7] found that multiple subsets of biomarkers undergo genetic changes, i.e., either activation or inactivation, during advancements and improvements of EOC Moreover, the deregulation of molecules in several cell signaling pathways, such as PI3K, AKT1 (AKT serine/threonine kinase 1), MET (MET proto-oncogene, receptor tyrosine kinase), HGF (Hepatocyte growth factor precursor), and their molecular crosstalk also play crucial roles in the molecular pathogenesis of E [8,9]. In addition, epigenetic alterations, such as DNA methylation and chromatin adjustment, are also accepted as crucial sharing factors in OC, and may provide useful molecular markers for disease advancement [10,11]. Though great advances have been made, the molecular mechanisms of EOC arefar from beingfully understood.

In recent years, microarray technology has been widely used to effect general genetic modifications during cancer progression [12,13]. Bioinformatics approaches are necessary to process the large amount of data that is generated by the microarray technology. In this analysis, we chose E-MTAB-3706 from array express, and used the limma R bioconductor package tool to detect differentially expressed genes (DEGs). Pathway enrichment analysis and gene enrichment analysis of up and down-regulated DEGs were carried out. We established a PPI network of the up- and down-regulated DEGs and picked out hub genes with high degrees of connectivity, high betweenness centrality, high stress centrality, high closeness centrality and low clustering coefficients. In addition, module analyses were performed. We also constructed a miRNA-target gene and TF- target gene interaction network. Overall survival (OS) analyses of these DEGs were made. Then, a correlation analysis based on TCGA database was performed to anticipate the probable relationship between genes, and served to inform the specific medicine treatment for patients. Therefore, it is a better way for us to further understand the molecular mechanisms of EOC.

## 2. Materials and Methods

### 2.1. Microarray Data

The microarray expression profile dataset E-MTAB-3706, which is based on the Illumina HumanHT-12_V4_0_R2_15002873_B Expression BeadChip (Illumina Way, San Diego, CA, USA), was downloaded from the European Bioinformatics Institute ArrayExpress database (https://www.ebi.ac.uk/arrayexpress/experiments/E-MTAB-3706/). The dataset contained 12 samples, including four high-grade ovarian epithelial cancer samples, four normal fallopian tube samples and four normal ovarian epithelium samples.

### 2.2. Identification of DEGs

Gene microarray analysis was handled through the R programming software (https://www.r-project.org/). Briefly, raw txt data were imported into R through lumi and beadarray version 3.6 package [14,15] and background correction, quantile normalization and microarray data condensation were performed. The common DEGs between high-grade ovarian epithelial cancer and normal ovarian epithelium were considered as the hub gens using limma package in R Bioconductor [16]. Genes with fold changes> 1 for up-regulated genes, fold changes >−1 for down-regulated genes and FDR (false discovery rate) < 0.0000015 were considered as highly significant.

### 2.3. Pathway Enrichment Analysis

Pathway enrichment analysis was performed using the online tool ToppCluster (http://toppcluster.cchmc.org) [17], which integrates different pathway databases such as The Kyoto Encyclopedia of Genes and Genomes (KEGG) (http://www.genome.jp/kegg/) [18], BIOCYC (https://biocyc.org/) [19], Pathway Interaction Database (PID) (http://pid.nci.nih.gov) [20], Reactome (http://www.reactome.org) [21], GenMAPP (http://www.genmapp.org/) [22], MSigDB C2 BIOCARTA (http://software.broadinstitute.org/gsea/msigdb/collections.jsp) [23], PantherDB (http://www.pantherdb.org/) [24], Pathway Ontology (https://bioportal.bioontology.org/ontologies/PW) [25] and SMPDB (http://smpdb.ca/) [26]. *p* ≤ 0.05 were considered to be significantly enriched pathways.

### 2.4. Gene Ontology (GO)Enrichment Analysis

Gene Ontology (GO) (http://www.geneontology.org/) includes the biological processes (BP), cellular components (CC) and molecular functions (MF) which are associated with genes and their products [27]. Gene ontology (GO) enrichment analysis was completed separately, using the ToppCluster (http://toppcluster.cchmc.org/) [17] online tool. *p* ≤ 0.05 was considered as the GO terms.

### 2.5. PPI Network Constructionand Analysis

To determine the interactive associations among the genes (up and down regulated) at the protein level, genes obtained from the EOC were mapped against protein-protein interaction (PPI) data using the online tool, InnateDB (http://www.innatedb.com) [28], which incorporates different PPI databases such as IntAc (https://www.ebi.ac.uk/intact/) [29], The Database of Interacting Proteins(DIP, https://dip.doe-mbi.ucla.edu/dip/Main.cgi) [30], The Molecular INTeraction Database (MINT, https://mint.bio.uniroma2.it/) [31], The Biomolecular Interaction Network Database (BIND, http://bind.ca) [32] and The Biological General Repository for Interaction Datasets (BioGRID, https://thebiogrid.org/) [33]. The topological properties (such as Node degree, betweenness centrality, stress centrality, closeness centrality and clustering coefficient) of the constructed PPI network were calculated using NetworkAnalyzer in Cytoscape (http://www.cytoscape.org/) [34].Briefly, in the networks, the nodes represent genes; the edges hinted at interactional relationships among them. The centrality of a network is expressed by the central degree, which is the granting of one gene to the genes in the proximity; this is expressed by the area of the nodes.The higher the degree value, the higher the area of the node. Therefore, important genes may be found from the PPI networks.

### 2.6. Module Analysis

To find modules ofhub genes in the PPI network, PEWCC1 [35] from Cytoscape (www.cytoscape.org/) [34] was subsequently applied. Module analysis resolves the associations between genes, and can help in the search for an important gene based upon complex regulatory associations.

### 2.7. Construction of the microRNA-Target Gene Regulatory Network

We obtained correlations between miRNAs and target genes using TarBase (http://www.diana.pcbi.upenn.edu/tarbase) [36] and the miRTarBase database (http://miRTarBase.mbc.nctu.edu.tw/) [37] through an online graph file generator NetworkAnalyst (http://www.networkanalyst.ca/) [38], which was visualized using Cytoscape version 3.6.0 (www.cytoscape.org/) [34].

### 2.8. Construction of the TFs-Target Gene Regulatory Network

The control of gene expression by TFs is crucial. The analysis of TF binding sites is necessary for research into gene control systems. In the present study, a transcriptional regulatory network was constructed using ChIP-X database (ChEA) (http://amp.pharm.mssm.edu/lib/chea.jsp) [39] using an online graph file generator NetworkAnalyst (http://www.networkanalyst.ca/) [38] and visualized using Cytoscape software version 3.6.0 (www.cytoscape.org/) [34].

### 2.9. Validation of the Expression Level of Hub Genes in PPI Network

The mRNA expression of the DEGs was analyzed in different stages of EOC with the assistance of UALCAN (http://ualcan.path.uab.edu/index.html) [40], which is an online tool thatdelivers customizable functionalities based on The Cancer Genome Atlas (TCGA).In the present study, the web tool was used to validate the expression levels of hub genes in the PPI network. A boxplot was generated to visualize any modified expression of hub genes.

### 2.10. Association of Hub Genes Expression with Survival of Patients with EOC

The online tool, UALCAN (http://ualcan.path.uab.edu/index.html) [40], was used to determine whether the hub genes could predict EOC patients’ survival. UALCAN integrates gene expression data with relapse-free and overall survival information from TCGA. Patients were categorized into high and low expression categories, according to the median value of hub gene expression. *p*-value < 0.05 was considered to be statistically significant.

## 3. Results

### 3.1. Data Source and DEGs Screening

**Figure 1:**
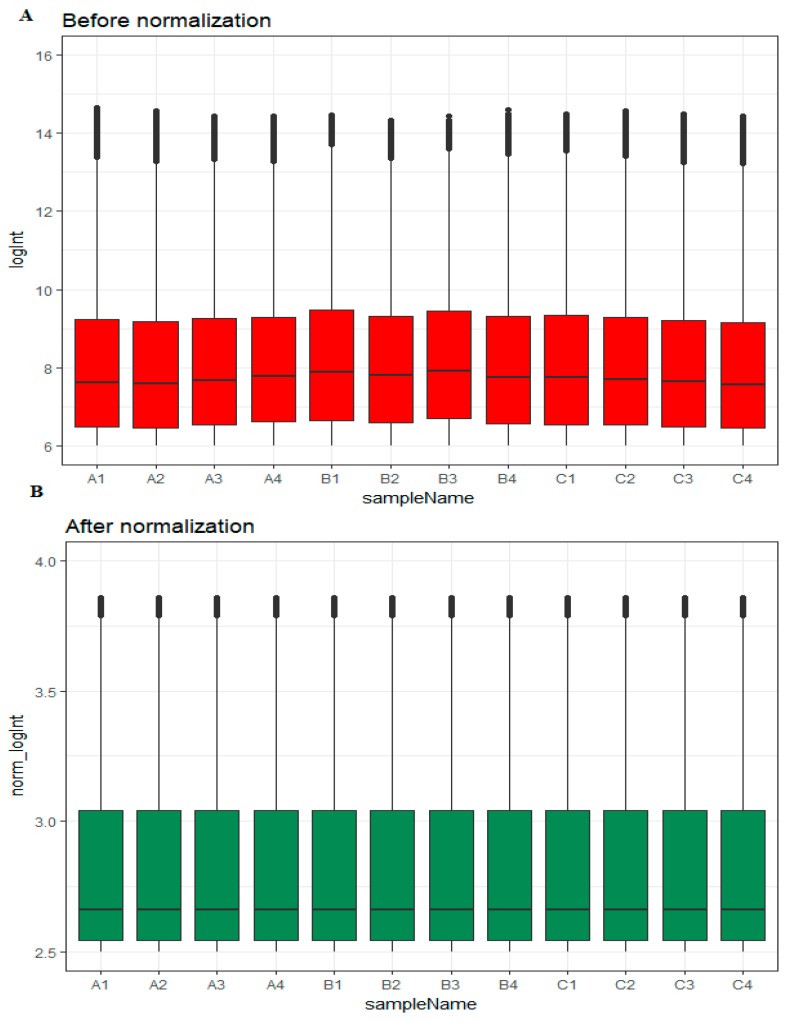
Box plots of the expression profiles. (**A**): Before normalization (red) and (**B**): after normalization (green). (A1, A2, A3, A4 = high-grade ovarian epithelial cancer, B1, B2, B3, B4 = normal fallopian tube samples, C1, C2, C3, C4 = normal ovarian epithelium samples).

Box plots before and after normalization of the raw data is shown in Figure 1A,B, respectively. Based on their EOC status, samples were divided into three groups: high-grade ovarian epithelial cancer (*n* = 4), normal fallopian tube (*n* = 4) and normal ovarian epithelium (*n* = 4).

**Figure 2:**
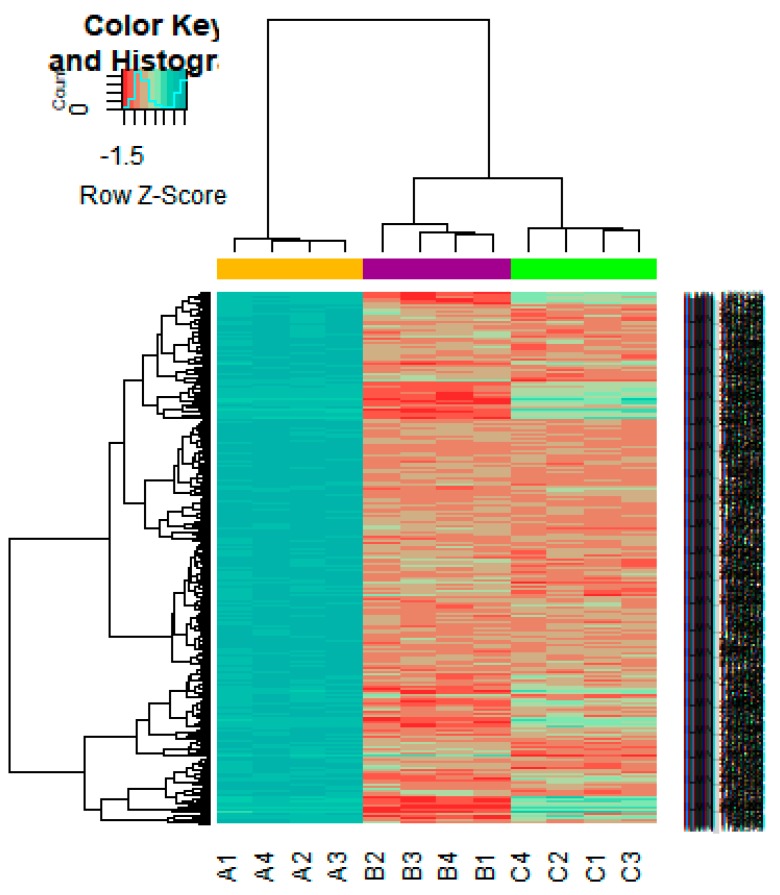
Heat map of gene expression (Up-regulated genes). The color key represents the logFCofDEGs. FC, fold change (A1, A2, A3, A4 = high-grade ovarian epithelial cancer, B1, B2, B3, B4 = normal fallopian tube samples, C1, C2, C3, C4 = normal ovarian epithelium samples).

**Figure 3:**
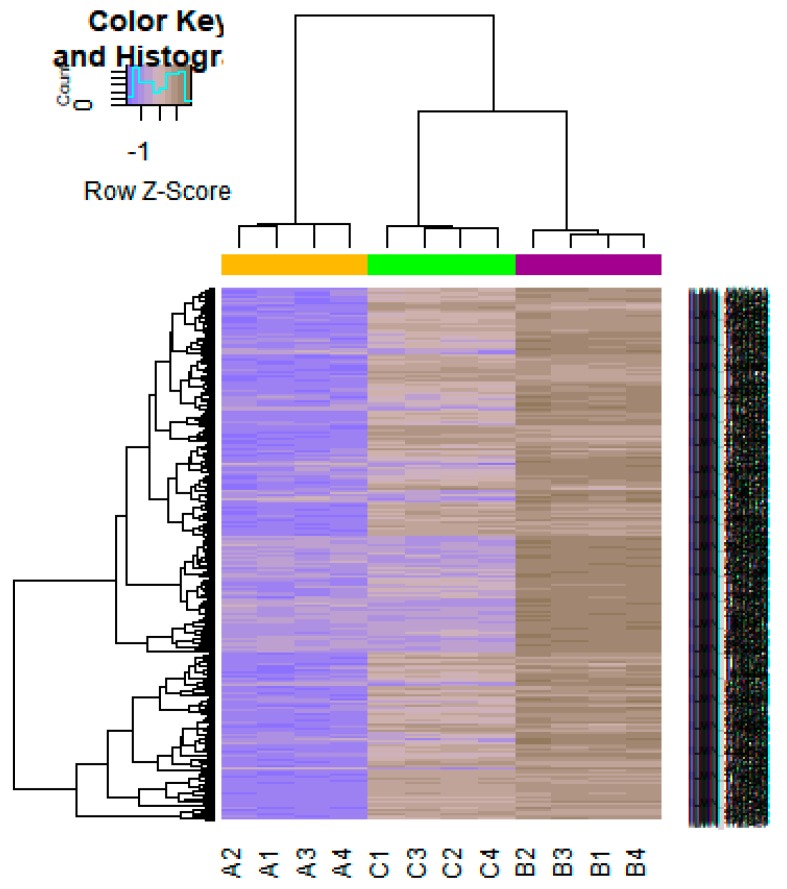
Heat map of gene expression (Down-regulated genes). The color key represents the logFC ofDEGs. FC, fold change (A1, A2, A3, A4 = high-grade ovarian epithelial cancer, B1, B2, B3, B4 = normal fallopian tube samples, C1, C2, C3, C4 = normal ovarian epithelium samples).

**Figure 4:**
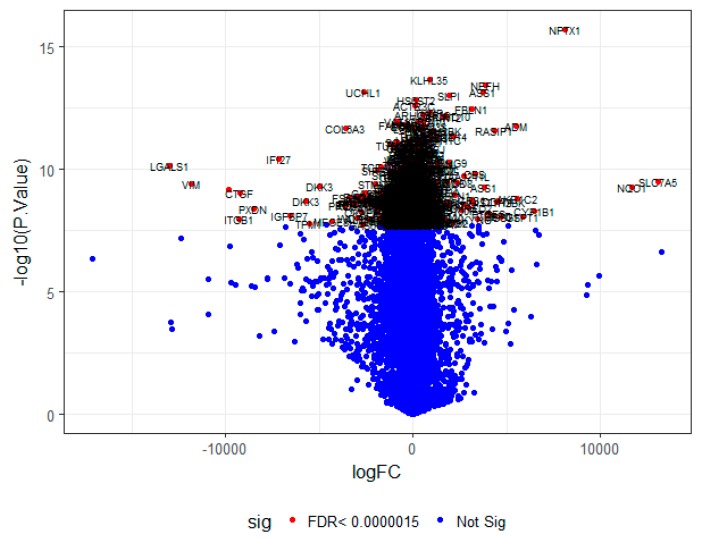
A volcano plot of differentially-expressed genes.

On the basis of the threshold criteria, a total of 552 DEGs were obtained, including 276up-regulated and 276 down-regulated genes in high-grade ovarian epithelial cancer samples, compared with normal ovarian epithelium samples (supplementary Table S1). FDR< 0.0000015, |logFC (fold change)| > 1 for up-regulated genes and |logFC| >− 1 for down-regulated genes. The hierarchical clustering analysis of up and down regulated genes in the dataset is shown in Figure 2 and Figure 3. A volcano plot was constructed to show the DEGs which might play key roles in EOC (Figure 4).

### 3.2. Pathway Enrichment Analysis

To initially comprehend the action of the genes, we submitted up and down regulated genes, respectively, to the online software, ToppCluster, to identify related pathways from different pathway databases such as KEGG, BIOCYC, PID, Reactome, GenMAPP, MSigDB C2 BIOCARTA, PantherDB, Pathway Ontology and SMPDB. Pathway enrichment analysis results showed that up-regulated genes were significantly enriched in chemical carcinogenesis, cell adhesion molecules (CAMs), urea cycle, creatine biosynthesis, EPHA2 forward signaling, syndecan-4-mediated signaling events, packaging of telomere ends, extracellular matrix organization, arginine and proline metabolism, glutathione metabolism, genes encoding enzymes and their regulators involved in the remodeling of the extracellular matrix, ensemble of genes encoding core extracellular matrix including ECM (Extracellular matrix) glycoproteins, collagens and proteoglycans, nicotine degradation, integrin signaling pathway, arginine and proline metabolic, ubiquitin/proteasome degradation, verapamil pathway and diltiazem pathway (supplementary Table S2).Down-regulated genes were mainly significantly enriched in the cell adhesion molecules (CAMs), axon guidance, creatine biosynthesis, 1D-myo-inositol hexakisphosphate biosynthesis II (mammalian), syndecan-4-mediated signaling events, amb2 integrin signaling, extracellular matrix organization, integrin cell surface interactions, glutathione metabolism, ensemble of genes encoding core extracellular matrix including ECM glycoproteins, collagens and proteoglycans, genes encoding structural ECM, integrin signaling pathway, methionine biosynthesis, ubiquitin/proteasome degradation, altered ubiquitin/proteasome degradation, guanidinoacetatemethyltransferase deficiency (GAMT Deficiency) and inositol metabolism(supplementary Table S3).

### 3.3. Gene Ontology Enrichment Analysis

According to the functional annotation in the GO database, the up-regulated geneswere mostly enriched in biological processes (BP) related to the translation andpeptide biosynthetic process, cellular component (CC) terms such as nucleosome and DNA packaging complex, and molecular function (MF) terms related to serine-type endopeptidase inhibitor activity and peptidase regulator activity. The GO enrichment terms of BP, CC, and MF for up-regulated genes are listed in supplementary Table S4. Meanwhile, the down-regulated genes were enriched in BP terms such as extracellular matrix organization and extracellular structure organization, CC terms such as extracellular matrix and proteinaceous extracellular matrix and MF terms such as cell adhesion molecule binding and peroxidase activity. The GO enrichment terms of BP, CC, and MF for down-regulated genes are listed in supplementary Table S5.

### 3.4. PPI Network Construction

**Figure 5:**
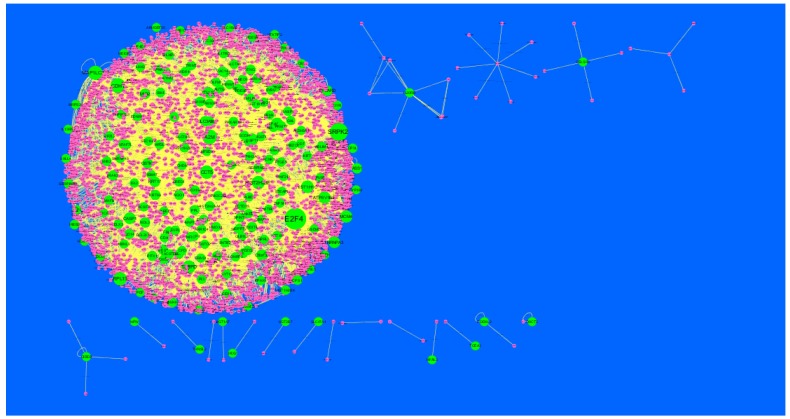
Protein-protein interaction (PPI) networks for differentially-expressed genes (DEGs). (Dark green round shape node represents up-regulated genes). Pink dots are also genes which are not hub or differential expressed genes. White rod shape is interaction with adjutant genes.

Node degree distribution was defined as the number of actual binding partners of a given protein [41]. The PPI network had (up-regulated) 4288 nodes and 8466 interactions (Figure 5). The hub gene with grater degrees such as E2F4 (degree = 479), SRPK2 (degree = 288), A2M (degree = 169), CDH1 (degree = 479) and MAP1LC3A (degree = 153) are listed in supplementary Table S6. 

**Figure 6:**
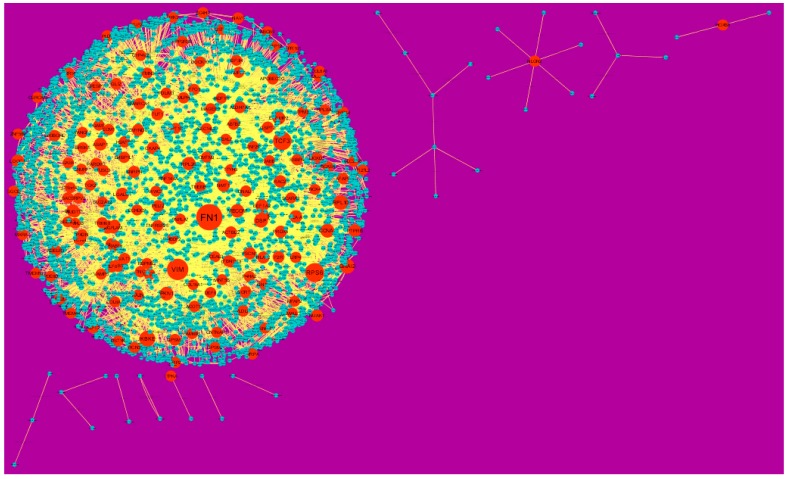
Protein-protein interaction (PPI) networks for differentially-expressed genes (DEGs). (Orange round shape node represents down-regulated genes). Green dots are also genes which are not hub or differential expressed genes. orange rod shape is interaction with adjutant genes.

**Figure 7:**
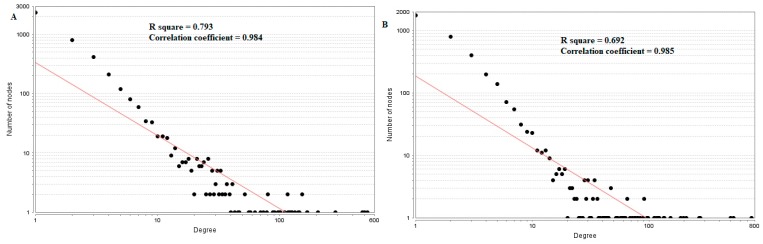
Node degree distribution for up (**A**) and down (**B**) regulated genes. Red lines: regression lines.

In the PPI network (down-regulated) had 3644 nodes and 7700 interactions (Figure 6).Hub genes with higher degrees such as FN1 (degree = 755), VIM (vimentin) (degree = 268), RPS6 (degree = 197), TCF3 (degree = 187) and IKBKE (inhibitor of nuclear factor kappa B kinase subunit epsilon) (degree = 117) are listed in supplementary Table S6. R square = 0.793 and correlation coefficient = 0.984 for node up regulated) (Figure 7A), meanwhile R square = 0.692 and correlation coefficient = 0.985 for node degree (down regulated) (Figure 7B).

### 3.5. Analysis of Topological Features of Nodes in PPI Network

**Figure 8:**
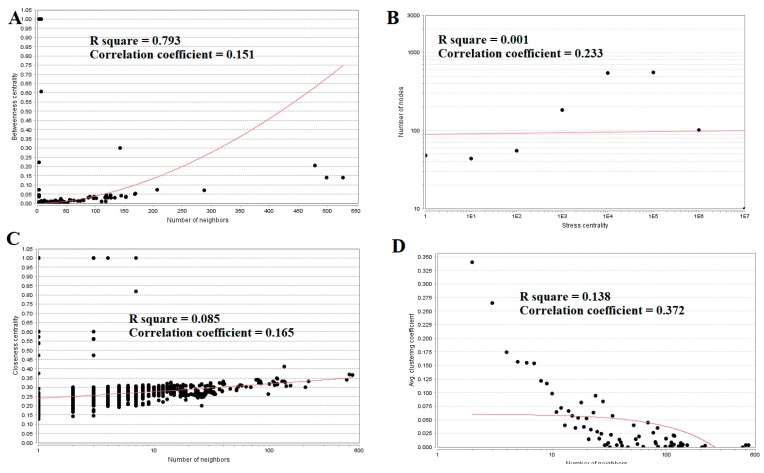
Regression diagrams for up-regulated genes ((**A**) Betweenness centrality; (**B**) Stress centrality; (**C**) Closeness centrality; (**D**) Clustering coefficient). The red straight lines and the red curve lines: regression lines.

Topological properties (betweenness centrality, stress centrality, closeness centrality and clustering coefficient) were analyzed for the PPI network.Betweenness centrality calibrates the progress of information through a node in the PPI network and helps in locating crucial but not very highly-connected nodes [42]. Hub genes(up-regulated) with high betweennesssuch as E2F4 (betweenness = 0.205549), CEBPD (betweenness = 0.046304), CCT5 (betweenness = 0.046304), ATP6V1B1 (betweenness = 0.032117) and MCM4 (betweenness = 0.027287) are listed in supplementary Table S6. R square = 0.270 and correlation coefficient = 0.151 for betweenness centrality (Figure 8A). The stress centrality of a node n is the number of shortest paths fleeting through n. A node has greater stress if it is crossed by a greater number of shortest paths [43]. Hub genes (up-regulated) with high stress such as SRPK2 (stress = 22460310), E2F4 (stress = 20592222), CDH1 (stress = 20592222), CCT5 (stress = 10033170) and HIST2H2BE (stress = 8392118) are listed in supplementary Table S6. R square = 0.001 and correlation coefficient = 0.233 for stress centrality (Figure 8B).Closeness centrality is a quota of centrality in a PPI network, which illustrates the moderate speed with which around walking processes influence a node from other nodes of the PPI network [44]. Hub genes (up-regulated) with high closeness centrality such asIL20RB (closeness = 0.818182), HNRNPA3 (closeness = 0.341767), HIST1H1C (closeness = 0.336839), MCM4 (closeness = 0.332809) and SRPK2 (closeness = 0.332547) are listed in supplementary Table S6. R square = 0.085 and correlation coefficient = 0.165 for closeness centrality (Figure 8C). A crucial peculiarity of scale-free PPI networks is the clustering coefficient, which indicates the reduction of node degree expansion [45]. Hub genes (up-regulated) with low clustering coefficients such as NPTX1 (clustering coefficient = 0), KLHL35 (clustering coefficient = 0), NEFH (neurofilament heavy) (clustering coefficient = 0), SLPI (clustering coefficient = 0) and TYMP (thymidine phosphorylase) (clustering coefficient = 0) are listed in supplementary Table S6. R square = 0.138 and correlation coefficient = 0.372 for clustering coefficient (Figure 8D).

**Figure 9:**
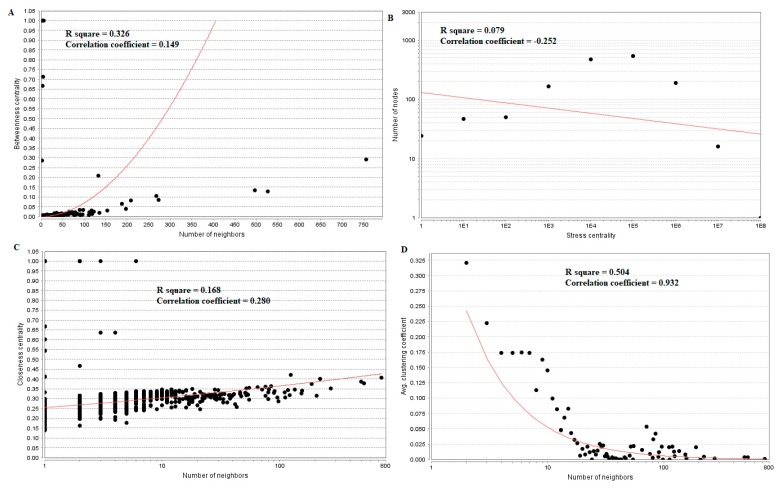
Regression diagrams for Down-regulated genes ((**A**) Betweenness centrality; (**B**) Stress centrality; (**C**) Closeness centrality; (**D**) Clustering coefficient). The red straight lines and the red curve lines: regression lines.

Hub genes (down-regulated) with high betweenness centralitysuch as UCHL1 (betweeness = 0.015169), HLA-C (major histocompatibility complex, class I, C) (betweeness = 0.015169), VAT1 (betweeness = 0.007237), ECM1 (betweeness = 0.007237) and SNRPN (small nuclear ribonucleoprotein polypeptide N) (betweeness = 0.006875) are listed in supplementary Table S6. R square = 0.326 and correlation coefficient = 0.149 for betweenness centrality (Figure 9A). Hub genes (down-regulated) with high stress such as FN1 (stress = 213338628), TCF3 (stress = 18049254), RPS6 (stress = 11434072), EFEMP2 (stress = 10312416) and MECOM (MDS1 and EVI1 complex locus) (stress = 8070650) are listed in supplementary Table S6. R square = 0.079 and correlation coefficient = −0.252 for stress centrality (Figure 9B). Hub genes (down-regulated) with high closeness centrality, such as FN1 (closeness = 0.407621), VIM (closeness = 0.400111), RPS6 (closeness = 0.373924), RPL26 (closeness = 0.354613) and LGALS1 (closeness = 0.351433),are listed in supplementary Table S6. R square = 0.168 and correlation coefficient = 0.280 for closeness centrality (Figure 9C). Hub genes (down-regulated) with low clustering coefficientsuch as FAM69B (clustering coefficient = 0), COL6A3 (clustering coefficient = 0), SNURF (SNRPN upstream reading frame) (clustering coefficient = 0), SCARA3 (clustering coefficient = 0) and TMEM107 (clustering coefficient = 0) are listed in supplementary Table S6. R square = 0.504 and correlation coefficient = 0.932 for clustering coefficient (Figure 9D).

### 3.6. Module Analysis

**Figure 10:**
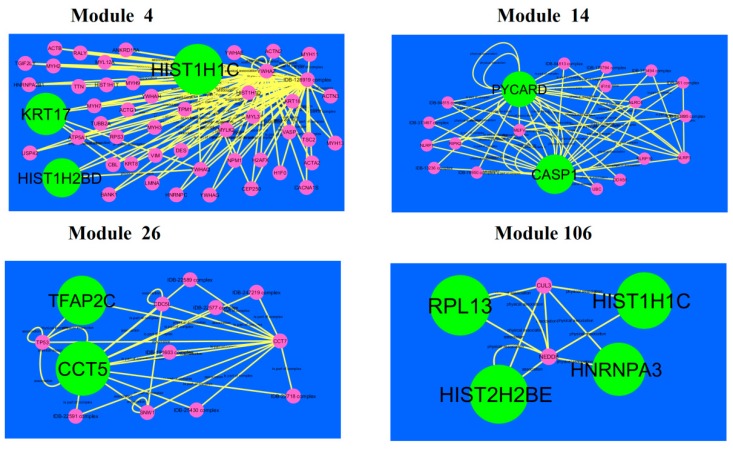
Modules in PPI network. Thedark green round shape nodes denote the up-regulated genes. Pink dots are also genes which are not hub or differential expressed genes. Yellow curve lines is interaction with adjutant genes.

A total of 921 modules were extracted from PPI network (up-regulated).Modules 4, 14, 26 and 106 were more significant (Figure 10). Module 4 had 50 nodes and 106 edges. Hub genes such as HIST1H2BD (degree = 18), HIST1H1C (degree = 102) and KRT17 (degree = 41) were involved in module 4.Module 14wascomposed of 20 nodes and 56 edges. Hub genes such as PYCARD (PYD and CARD domain containing) (degree = 47) and CASP1 (degree = 79) were involved in module 14.Module 26 had13 nodes and 29 edges. Hub genes such as TFAP2C (degree = 91) and CCT5 (degree = 126) were involved in module 29.Module 106had 6 nodes and 11 edges. Hub genes such as RPL13 (degree = 127), HIST1H1C (degree = 102), HNRNPA3 (degree = 80) and HIST2H2BE (degree = 117) were involved in module 29.

**Figure 11:**
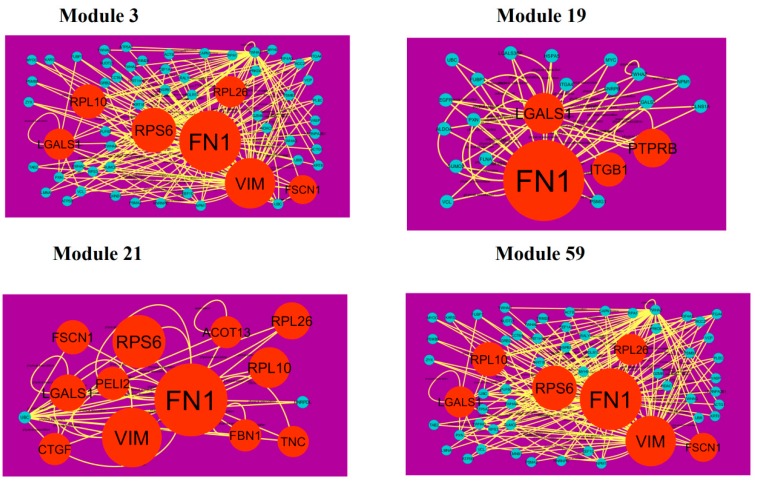
Modules in PPI network. The orange round nodes denote down-regulated genes. Green dots are also genes which are not hub or differential expressed genes. Yellow curve lines is interaction with adjutant genes.

A total of 939 modules were extracted from the PPI network (down-regulated). Modules 3, 19, 21 and 59 were more significant (Figure 11). Module 3 had 61 nodes and 173 edges. Hub genes such as FN1 (degree = 755), FSCN1 (degree = 34), RPL10 (degree = 91), VIM (degree = 268), RPS6 (degree = 197), LGALS1 (degree = 57) and RPL26 (degree = 59) were involved in module 3. Module 19 had 22 nodes and 51 edges. Hub genes such as ITGB1 (degree = 5), PTPRB (protein tyrosine phosphatase, receptor type B) (degree = 40), FN1 (degree = 755) and LGALS1 (degree = 57) were involved in module 19. Module 21 had 14 nodes and 38 edges. Hub genes such as FSCN1 (degree = 755), RPL26 (degree = 72), FN1 (degree = 72), RPS6 (degree = 197), RPL10 (degree = 197), VIM (degree = 91), LGALS1 (degree = 57), CTGF (connective tissue growth factor) (degree = 16), PELI2 (degree = 25), ACOT13 (degree = 15), FBN1 (degree = 1) and TNC (tenascin C) (degree = 8) were involved in module 21. Module 59 had 7 nodes and 15 edges. Hub genes such as EEF1A2 (degree = 54), ACTBL2 (degree = 54), RPS6 (degree = 197) and DSP (desmoplakin) (degree = 72) were involved in module 59.

### 3.7. Construction of the miRNA-Target Gene Regulatory Network

**Figure 12:**
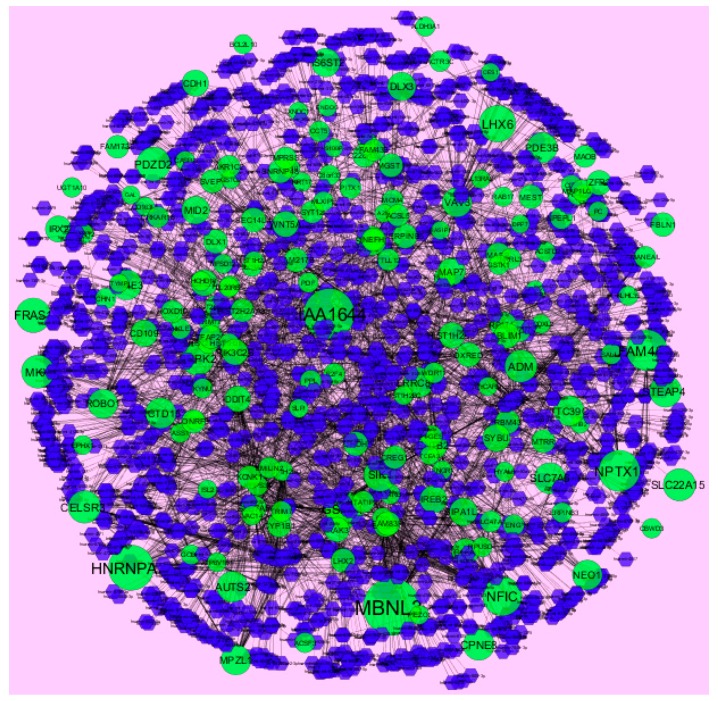
The network of up-regulated DEGs and their related miRNAs. The green circles nodes are up-regulated DEGs, and blue diamond nodes are miRNAs. Black lines means interaction with adjutant genes or miRNAs.

**Figure 13:**
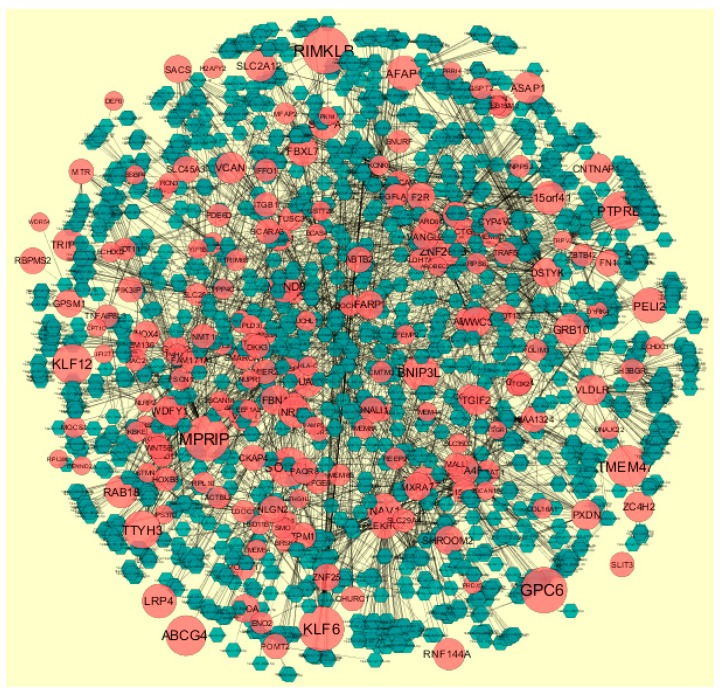
The network of down-regulated DEGs and their related miRNAs. The red circle nodes are the down-regulated DEGs, and green diamond nodes are miRNAs. Black lines means interaction with adjutant genes or miRNAs.

MicroRNAs (miRNAs) are endogenous, small non-coding RNAs whose actions control biomarker expression. The use of miRNAs as potential molecular markers for human cancer diagnosis, prognosis and therapeutic targets or tools, urgently study further examination and acceptance [46]. The miRNA for target up- and down-regulated genes is shown in Figure 12 and Figure 13. Of the up regulated hub genes, e.g., MBNL3 interacts with 308 miRNAs, KIAA1644 interacts with 265 miRNAs, HNRNPA3 interacts with 227 miRNAs and NPTX1 interacts with 200 miRNAs, as listed in supplementary Table S7. Of the down regulated hub genes, e.g., DSP (ribosomal modification protein rimK like family member B) interacts with 242 miRNAs, GPC6 interacts with 233 miRNAs, MPRIP (myosin phosphatase Rho interacting protein) interacts with 230 miRNAs, KLF6 interacts with 227 miRNAs and TMEM47 interacts with 203 miRNAs, as listed in supplementary Table S7.

### 3.8. Construction of the TFs-Target Gene Regulatory Network

**Figure 14:**
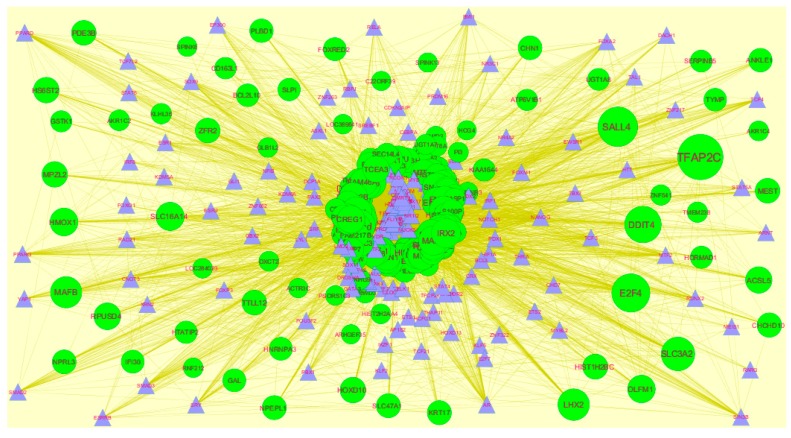
TF- gene network of predicted target up-regulated genes. Blue triangles are TFs and green circles are target up-regulated genes. Yellow lines means interaction with adjutant genes or TFs.

**Figure 15:**
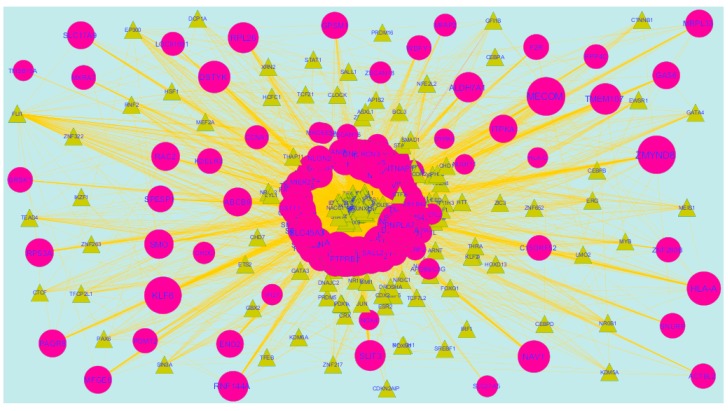
TF- gene network of predicted target down-regulated genes. Yellow triangles are TFs and pink circles are target down-regulated genes. Yellow lines means interaction with adjutant genes or TFs.

Transcription factors (TFs)were responsible for the advancement of many cancer types [47]. The TFs for target up- and down-regulated genes are shown in Figure 14 and Figure 15, respectively.O fht up-regulated hub genes, e.g.,KRT6A interacts with 124 TFs, MRPS30 interacts with 109 TFs, FAM217B interacts with 100 TFs, MFSD3 interacts with 95 TFs and KIAA1644 interacts with 92 TFs, as listed in supplementary Table S8. Of the down-regulated hub genes, e.g., TMSB15A interacts with 120 TFs, ZNF280B interacts with 100 TFs, BRSK1 interacts with 105 TFs, IFI27L2 interacts with 103 TFs and RIMKLB interacts 99 TFs, as listed in supplementary Table S8.

### 3.9. Validation of the Expression Level of Key Genes in PPI Network

**Figure 16:**
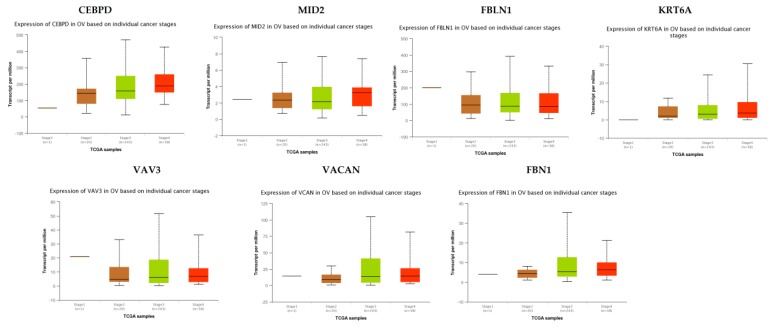
Validation of the expression of hub genes in TCGA database. High expression of hub genes in stages 3 and 4. CEBPD: CCAAT enhancer binding protein delta; OV: ovarian cancer.

**Figure 17:**
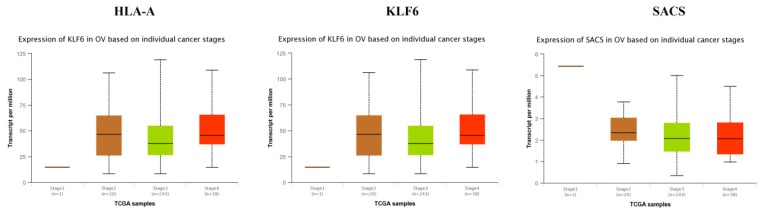
Validation of the expression of hub genes in the TCGA database. High expression of hub genes in stages 2 and 4.

TCGA data analysis showed that hub genes such as CEBPD, MID2, FBLN1, KRT6A, VAV3, VACAN and FBN1 were highly expressed in stages 3 and 4 compared to other stages (Figure 16), while hub genes such as HLA-A (major histocompatibility complex, class I, A), KLF6 and SACS (sacsin molecular chaperone) were highly expressed in stages 2 and 4 compared to other stages(Figure 17); these findings were consistent with the results of the microarray analysis.

### 3.10. Association of Hub Genes Expression with Survival of Patients with EOC

**Figure 18:**
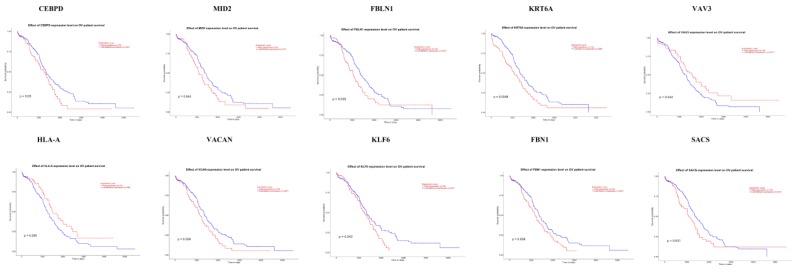
Kaplan-Meier survival curves using TCGA data validate the prognostic value of genes expressed in EOC. Blue is low expression and Red is high expression).

The UALCAN online analysis tool was used to analyze the prognosis of EOC according to the expression (low and high) of the hub genes. The analysis tool can do Kaplan–Meier prognosis analyses based on hub gene expression (low and high) and prognostic correlations in the TCGA database. As shown in Figure 18, the high expression levels of the hub genes such as FBLN1, VAV3, HLA-A and SACS correlated with favorable overall survival (OS) for EOC, while hub genes such as CEBPD, MID2, KRT6A, VACAN, KLF6 and FBN1 correlated with worst overall survival (OS) for EOC.

## 4. Discussion

Symbolic progress has been accomplished in the diagnosis and treatment of EOC. However, EOC remains the fifth most common cancer worldwide [48]. Due to the high risk of recurrence and the poor survival rates of metastatic EOC patients, high-grade ovarian epithelial cancer, normal ovarian epithelium and normal fallopian tube were used to investigate the molecular mechanisms which are associated with metastasis. In previous studies, gene expression profiling has been used to identify the biomarkers and pathways which are associated with EOC [49], as well as genome-wide changes in EOC [50].Based on the cutoff criteria, a total of 276 up-regulated and 276 down-regulated genes were diagnosed from E-MTAB-3706. Methylation inactivation ASS1 was linked with cisplatin resistance in EOC [51]. Over expression of SLPI was associated with pathogenesis of EOC [52,53]. HS6ST2 was critical for fibroblast growth factor 2 activation [54]. HS6ST2 was responsible for angiogenesis in colorectal cancer [55]. HS6ST2 is associated with angiogenesis in EOC [56]. FBLN1 was associated with invasion of EOC cells [57,58]. ADM (adrenomedullin) was linked with angiogenesis in EOC [59]. ADM was responsible forpathogenesis of EOC [60]. UCHL1 was associated with growth breast cancer [61]. The loss of tumor suppressor UCHL1 was responsible for the inactivation of apoptosis, as well as cisplatin resistance, in EOC [62]. Methylation inactivation of tumor suppressors such as UCHL1 [63] and TUSC3 [64] were diagnosed with EOC.IFI27 was responsible for the proliferation of epithelial cancer cells [65].Li IFI27 was associated with invasion and drug resistance in EOC [66]. The loss of tumor suppressor DKK3 was important for invasion of cervical cancer [67]. The methylation inactivation of tumor suppressor DKK3 was linked with pathogenesis of gastric cancer [68].The loss of DKK3 was responsible for the progression of EOC [69].The loss of GSTT1 was responsible for the inactivation of the detoxification processes in EOC [70]. Polymorphism in GSTT1 is important for the improvement of EOC [71,72].

In pathway enrichment analyses, chemical carcinogenesis, urea cycle, packaging of telomere ends, arginine and proline metabolism, genes encoding enzymes and their regulators involved in the remodeling of the extracellular, nicotine degradation, EPHA2 forward signaling, arginine and proline metabolic, and verapamil pathway were the most significant pathways from different pathway databases such as KEGG, BIOCYC, PID, Reactome, GenMAPP, MSigDB C2 BIOCARTA, PantherDB, Pathway Ontology and SMPDB for up-regulated genes.CYP1B1 was responsible for drug resistance in EOC [73]. Polymorphisms in EPHX1 [74] and GSTO2 [75] were responsible for the pathogenesis of EOC. MGST1 was linked with the invasion and chemoresistance in EOC [76].The loss of AKR1C2 was responsible for the advancement of prostate cancer [77], but the inactivation of this gene may be associated with the development of EOC. ALDH3A1 was responsible for drug resistance in breast cancer [78], but this gene may be associated with drug resistance in EOC. Polymorphism in UGT1A7 was an indicator of drug toxicity in colorectal cancer [79], but this polymorphic gene may be associated with drug toxicity in EOC. Mutation in UGT2B7 was important for the growth of breast cancer [80], but mutations in this gene may be responsible for the development of EOC. Mutations in ASS1 were involved in the development of lung cancer [81], but mutations in this gene may be linked with pathogenesis of EOC. VAV3 was linked with metastasis of EOC [82]. Single nucleotide polymorphism (SNP) in EFNA1 was important for the progression of gastric cancer [83], but SNP in this gene may be associated with development of EOC. SERPINB3 was associated with invasion of EOC cells [84,85]. CD109 was found to be associated with the growth of small cell lung cancer [86], but this gene may be associated with the pathogenesis of EOC. Methylation inactivation of EDNRB (endothelin receptor type B) was linked with the development ofprostate cancer [87], but the inactivation of this gene may be responsible for the progression of EOC. GSTK1, UGT1A10, UGT1A8, HIST1H2AC, HIST1H2BC, HIST1H2BD, HIST1H2BJ, HIST1H2BK, HIST2H2AA3, HIST2H2AA4, HIST2H2BE, MAOB (monoamine oxidase B), A2M, PI3, HYAL3, KCNK1, SNTB1 and SNTB2 were novel biomarkers for pathogenesis of EOC in these pathways. Meanwhile, cell adhesion molecules, creatine biosynthesis, syndecan-4-mediated signaling event, extracellular matrix organization, glutathione metabolism, the ensemble of genes encoding core extracellular matrix, integrin signaling pathway, ubiquitin/proteasome degradation and guanidinoacetatemethyltransferase deficiency (GAMT Deficiency) were the most significant pathways from different pathway databases such as KEGG, BIOCYC, PID, Reactome, GenMAPP, MSigDB C2 BIOCARTA, PantherDB, Pathway Ontology and SMPDB for down-regulated genes.NECTIN2was linked with cell adhesion and the migration of pancreatic ductal adenocarcinomas cells [88]. NECTIN2 was important for invasion and metastasis in colorectal carcinoma [89]. NECTIN2 was important for the pathogenesis of EOC [90]. VCAN was associated with the progression of prostate cancer [91]. VCAN was responsible for invasion of EOC cells [92]. Loss of HLA-A was found to correlated with pathogenesis of colorectal cancer, but the reduced expression of this gene may be associated with EOC [93]. ITGB1 was involved in cell adhesion and invasion of prostate cancer cells [94], but this gene may be associated with invasion of EOC. NECTIN3 was associated with the proliferation of colon cancer cells [95], but this gene may be linked with the development of EOC. TNC (tenascin C) was responsible for invasion of colon cancer cells [96] but was important for metastasis and angiogenesis in EOC [97]. FN1 was linked with the suppression of apoptosis in renal cancer [98], but this gene may be associated with the suppression of apoptosis in EOC. COL6A3 was responsible for the advancement of EOC [99]. FBN1 was responsible for the pathogenesis of EOC [100]. Polymorphism in GSTM1 was associated with cancer risk [101,102,103]. CTGF (connective tissue growth factor) was involved in the growth of breast cancer [104]. Methylation inactivation of tumor suppressor gene CTGF was responsible for the development of EOC [105]. GAS6 was important for the migration and invasion of prostate cancer cells [106]. GAS6 was associated with invasion of EOC cells [107]. Low expression of tumor suppressor IGFBP7 was responsible for angiogenesis in hepatocellular carcinoma [108]. The loss of IGFBP7 was found to be associated with angiogenesis in EOC [109]. MFGE8 was important for pathogenesis of prostate cancer [110]. MFGE8 was associated with the adhesion and migration of EOC cells [111]. Metylation inactivation of tumor suppressor SLIT3 was responsible for invasion of thyroid cancer cells [112], but silencing this gene may be associated with invasion of EOC. ECM1 was linked with angiogenesis and metastasis of breast cancer cells [113], but this gene may be responsible for metastasis of EOC. RAC2 was diagnosed with the growth ofbrain cancer [114], but this gene may be linked with the development of EOC. CNTNAP1, HLA-C, JAM3,NLGN2, COL16A1, COL8A1, EFEMP2, LRP4, MFAP2, P3H3, P4HA2, GSTT2, EGFLAM (EGF like, fibronectin type III and laminin G domains), PXDN (peroxidasin), ACTBL2, SNCA (synuclein alpha) and GAMT (guanidinoacetate N-methyltransferase) were novel biomarkers for pathogenesis of EOC in these pathways.

In GO enrichment analysis, cellular response to xenobiotic stimulus, nucleosome and serine-type endopeptidase inhibitor activity were the most significant GO terms, i.e., for BP, CC and MF for up-regulated genes. Polymorphism in NQO1 was associated with drug resistance in non-small cell lung cancer [115]. Polymorphism in NQO1 was responsible for the development of EOC [116]. Low expression of tumor suppressor SPINK13 was linked with invasion of EOC cells [117]. Methylation inactivation of tumor suppressor SPINT2 was diagnosed with the growth of melanoma [118], but the inactivation of this gene may be associated with the development of EOC.CES1, HIST1H1C andSPINK6 were novel biomarkers for pathogenesis of EOC in these GO terms. Meanwhile, extracellular matrix organization, extracellular matrix and cell adhesion molecule binding were the most significant GO terms for e.g. BP, CC and MF for down-regulated genes. FSCN1 was responsible for invasion of bladder cancer cells [119]. FSCN1 was linked with invasion of EOC cells [120]. GPC6 was involved in invasion of EOC cells [121]. Metylation inactivation of LGALS1 was associated with angiogenesis in colorectal cancer [122], but silencing this gene may be responsible for angiogenesis in EOC. NECTIN2 was associated with the proliferation of EOC cells [90].WNT5B and DSP were novel biomarker for pathogenesis of EOC in these GO terms.

In the PPI network (up regulated), hub genes such as E2F4, SRPK2, A2M, CDH1 and MAP1LC3A were identified with high node degrees. E2F4was linked with the proliferation of breast cancer cells [123], but this gene may be responsible for the proliferation of EOC cells. CDH1 was responsible for metastasis of gastric cancer and colorectal cancer [124]. Mutation in CDH1 was responsible for metastasis of EOC [125]. Hub genes with highest betweenness centralities such as E2F4, CEBPD, CCT5, ATP6V1B1 and MCM4. MCM4 was identified with the proliferation of non small cell lung cancer cells [126], but this gene may be responsible for the proliferation of EOC cells. Hub genes with highest stress centralities such as SRPK2, E2F4, CDH1, CCT5 and HIST2H2BE.Hub genes with highest closeness centrality such as IL20RB, HNRNPA3, HIST1H1C, MCM4 and SRPK2. Hub genes with lowest clustering coefficient such as NPTX1, KLHL35, NEFH, SLPI and TYMP. SRPK2, MAP1LC3A, CEBPD, CCT5, ATP6V1B1 and IL20RB were novel biomarkers for pathogenesis of EOC in this PPI network. Meanwhile, PPI network (down-regulated), hub genes such as UCHL1, HLA-C, VAT1, ECM1 and SNRPN were identified with high node degree. Hub genes with high betweenness such as UCHL1, HLA-C, VAT1, ECM1 and SNRPN. Hub genes with high stress such as FN1, TCF3, RPS6, EFEMP2 and MECOM. TCF3 was linked with drug resistance in gastric cancer [127], but this gene may be responsible for drug resistance in EOC. TCF3 was found to be associated with the growth of EOC [128]. MECOM was associated with the proliferation of glioblastoma multiforme cells [129]. MECOM was responsible for pathogenesis of EOC [130]. RPS6 was associated with drug resistance in colorectal cancer [131], but this gene may be responsible for drug resistance in EOC. Hub genes with high closeness such as FN1, VIM, RPS6, RPL26 and LGALS1. Hub genes with low clustering coefficient such as FAM69B, COL6A3, SNURF, SCARA3 and TMEM107. SCARA3 was associated with metastasis in EOC [132]. SNRPN, VIM, RPL26 and LGALS1 were novel biomarkers for pathogenesis of EOC in this PPI network.

In a module analysis for PPI network (up-regulated), hub genes such as HIST1H2BD, HIST1H1C, KRT17, PYCARD, CASP1, TFAP2C, CCT5, HNRNPA3 and HIST2H2BE were in all four modules. KRT17, KIF7, SLC7A5 and SLC3A2 were novel biomarkers for EOC in these modules. Meanwhile, in a module analysis for the PPI network (down-regulated), hub genes such as FN1, FSCN1, RPL10, VIM, RPS6, LGALS1,RPL26, ITGB1, PTPRB, LGALS1, CTGF, PELI2, ACOT13, FBN1, TNC, EEF1A2, ACTBL2and DSP were found to be in all four modules. EEF1A2 was associated with angiogenesis in breast cancer [133]. EEF1A2 was linked with pathogenesis of EOC [134]. RPL10, PTPRB, PELI2 and DSP were novel biomarkers for pathogenesis of EOC in these modules.

In the miRNA-target gene regulatory network (up-regulated), hub genes such as MBNL3, KIAA1644, HNRNPA3, NPTX1 and FAM46A. MBNL3, KIAA1644 and FAM46A were novel biomarkers for pathogenesis of EOC in this network. Meanwhile, in the miRNA-target gene regulatory network (down-regulated), hub genes such as RIMKLB, GPC6, MPRIP, KLF6 and TMEM47. The modification in tumor suppressor KLF6 was responsible for the advancement of prostate cancer [135]. The loss of tumor suppressor KLF6 was responsible for pathogenesis of EOC [136]. RIMKLB, MPRIP and TMEM47 were novel biomarkers for pathogenesis of EOC in this network.

In TF-target gene regulatory network (up-regulated), hub genes such as KRT6A, MRPS30, FAM217B, MFSD3 and KIAA1644. SNP in tumor suppressor MRPS30 was associated with advancement of breast cancer [137], but SNP in this gene may be responsible for pathogenesis of EOC. KRT6A, FAM217B and MFSD3 were novel biomarkers for pathogenesis of EOC in this network. In the TF-target gene regulatory network (down-regulated), hub genes such as TMSB15A, ZNF280B, BRSK1, IFI27L2 and RIMKLB. TMSB15A, ZNF280B, BRSK1 and IFI27L2 were novel biomarkers for pathogenesis of EOC in this network.

Survival analysis revealed that hub genes with high expression, such as FBLN1, VAV3, HLA-A and SACS were associated with improved survival in EOC, while hub genes with high expression such as CEBPD, MID2, KRT6A, VACAN, KLF6 and FBN1 showed the opposite tendency. Expression levels revealed that hub genes such as CEBPD, MID2, FBLN1, KRT6A, VAV3, VACAN and FBN1were highly expressed in EOC (stages 3 and 4), meanwhile those such as HLA-A, KLF6 and SACS were highly expressed in EOC (stages 2 and 4).

## 5. Conclusions

In conclusion, the purpose of this study was to improve our understanding of the molecular mechanisms underlying the progression of EOC through an integrated bioinformatics analysis that aimed to diagnose DEGs and the related pathways associated with the advancement of EOC. Our research also identified several hub genes and biological pathways that could aid in the search for novel biomarkers and therapeutic targets for the treatment ofEOC. However, further molecular biology experiments are required to validate the findings of this study.

## Supplementary Materials

The following are available online at https://www.mdpi.com/2075-4418/9/2/39/s1.
