# Supplementary material for: Mining Featured Biomarkers Linked with Epithelial Ovarian CancerBased on Bioinformatics
Source: Diagnostics (Basel). 2019 Apr 9;9(2):39. doi: 10.3390/diagnostics9020039 (PMC6628368; doi:10.3390/diagnostics9020039)
Supplement: Supplementary file 1 [file diagnostics-09-00039-s001.pdf]

Table 1. Differential gene expression (up and down regulation).

IlluminaId	GeneSymbol	logFC	p value	FDR	t value	Regulation	GeneName
ILMN_1814221	NPTX1	8120.166	2.02E-16	9.56E-12	107.0626	Up	neuronal pentraxin 1
ILMN_1693471	KLHL35	879.4869	2.03E-14	4.8E-10	66.98158	Up	kelch like family member 35
ILMN_1705153	NEFH	3866.938	3.55E-14	5.6E-10	63.27518	Up	neurofilament heavy
ILMN_1708778	ASS1	3751.105	6.81E-14	6.69E-10	59.20647	Up	argininosuccinate synthase 1
ILMN_2114720	SLPI	1949.583	9.25E-14	7.29E-10	57.39296	Up	secretory leukocyte peptidase inhibitor
ILMN_2415583	HS6ST2	150.4964	1.51E-13	1.02E-09	54.59292	Up	heparansulfate 6-O-sulfotransferase 2
ILMN_3185198	ACTR3C	112.0705	2.58E-13	1.53E-09	51.68897	Up	ARP3 actin-related protein 3 homolog C
ILMN_1700541	FBLN1	3126.79	3.36E-13	1.77E-09	50.31287	Up	fibulin 1
ILMN_2109708	TYMP	922.6535	4.87E-13	2.31E-09	48.44836	Up	thymidine phosphorylase
ILMN_2214473	ARHGEF35	555.516	5.87E-13	2.5E-09	47.53665	Up	Rho guanine nucleotide exchange factor 35
ILMN_1740170	CHCHD10	1770.965	6.4E-13	2.5E-09	47.11754	Up	coiled-coil-helix-coiled-coil-helix domain containing 10
ILMN_1800739	SPINT2	1656.074	6.86E-13	2.5E-09	46.78296	Up	serine peptidase inhibitor, Kunitz type 2
ILMN_3241522	ASS1	603.9339	1E-12	3.15E-09	45.0058	Up	argininosuccinate synthase 1
ILMN_1740233	UGT1A10	540.0026	1.1E-12	3.15E-09	44.5868	Up	UDP glucuronosyltransferase family 1 member A10
ILMN_3273229	MIR193BHG	273.3476	1.13E-12	3.15E-09	44.45755	Up	MIR193B host gene
ILMN_1748827	TMEM238	614.575	1.65E-12	3.8E-09	42.78856	Up	transmembrane protein 238
ILMN_1708934	ADM	5538.466	1.77E-12	3.8E-09	42.47741	Up	adrenomedullin
ILMN_2059797	NOL3	283.2824	2.07E-12	4.13E-09	41.80599	Up	nucleolar protein 3
ILMN_1720452	LONRF3	135.1837	2.18E-12	4.13E-09	41.57205	Up	LON peptidase N-terminal domain and ring finger 3
ILMN_2144426	HIST2H2AA3	435.4416	2.42E-12	4.35E-09	41.12968	Up	histone cluster 2 H2A family member a3
ILMN_1813314	HIST1H2BK	1064.222	2.48E-12	4.35E-09	41.03098	Up	histone cluster 1 H2B family member k
ILMN_1755657	RASIP1	4370.555	2.58E-12	4.37E-09	40.86067	Up	Ras interacting protein 1
ILMN_1742025	OLFM1	975.567	2.77E-12	4.45E-09	40.5662	Up	olfactomedin 1
ILMN_1682015	GAL	1105.312	2.82E-12	4.45E-09	40.49355	Up	galanin and GMAP prepropeptide
ILMN_1772957	FOXRED2	932.1938	3.54E-12	5.41E-09	39.56366	Up	FAD dependent oxidoreductase domain containing 2
ILMN_2396444	CD14	2188.76	4.19E-12	6.19E-09	38.89483	Up	CD14 molecule
ILMN_1741768	TMPRSS3	555.3086	4.37E-12	6.27E-09	38.72194	Up	transmembrane protease, serine 3
ILMN_1718972	MFSD3	935.4845	4.72E-12	6.57E-09	38.4233	Up	major facilitator superfamily domain containing 3
ILMN_1740234	GSTO2	524.5634	5E-12	6.76E-09	38.19439	Up	glutathione S-transferase omega 2
ILMN_1731086	ISL2	244.467	5.81E-12	7.48E-09	37.61258	Up	ISL LIM homeobox 2
ILMN_1838313	LRRC8B	215.5037	5.85E-12	7.48E-09	37.5879	Up	leucine rich repeat containing 8 family member B
ILMN_1757406	HIST1H1C	1258.306	6.09E-12	7.58E-09	37.43439	Up	histone cluster 1 H1 family member c
ILMN_1654916	KIF7	408.1329	7.44E-12	8.61E-09	36.67483	Up	kinesin family member 7
ILMN_1714709	OLFM1	679.8296	7.75E-12	8.73E-09	36.52295	Up	olfactomedin 1
ILMN_1767523	IL17RB	269.9503	9.33E-12	1.01E-08	35.83367	Up	interleukin 17 receptor B
ILMN_2095704	SYCP2	349.3677	1.14E-11	1.15E-08	35.10275	Up	synaptonemal complex protein 2
ILMN_2071641	KCNK1	122.4703	1.25E-11	1.22E-08	34.78411	Up	potassium two pore domain channel subfamily K member 1
ILMN_1760180	MID2	190.6607	1.34E-11	1.25E-08	34.52547	Up	midline 2
ILMN_1658702	HIST1H2BJ	276.0322	1.4E-11	1.27E-08	34.38902	Up	histone cluster 1 H2B family member j
ILMN_3243457	ANKLE1	128.7505	1.47E-11	1.31E-08	34.19914	Up	ankyrin repeat and LEM domain containing 1

ILMN_1738989	SYBU	333.2647	1.66E-11	1.46E-08	33.77447	Up	syntabulin
ILMN_2061537	UGT1A8	109.3142	1.74E-11	1.49E-08	33.62886	Up	UDP glucuronosyltransferase family 1 member A8
ILMN_1775542	FCMR	368.2925	1.83E-11	1.55E-08	33.44337	Up	Fc fragment of IgM receptor
ILMN_2346316	PTPN20	185.2835	2E-11	1.66E-08	33.13919	Up	protein tyrosine phosphatase, non-receptor type 20
ILMN_1764709	MAFB	230.0595	2.09E-11	1.71E-08	32.99049	Up	MAF bZIP transcription factor B
ILMN_1782412	IRX2	558.6375	2.26E-11	1.75E-08	32.7352	Up	iroquoishomeobox 2
ILMN_1657451	SRPK2	248.7737	2.26E-11	1.75E-08	32.72759	Up	SRSF protein kinase 2
ILMN_1787259	RNF212	361.6083	2.26E-11	1.75E-08	32.72537	Up	ring finger protein 212
ILMN_1704987	ZFR2	989.1009	2.3E-11	1.75E-08	32.67869	Up	zinc finger RNA binding protein 2
ILMN_1742947	SEC14L4	232.5852	2.47E-11	1.82E-08	32.43813	Up	SEC14 like lipid binding 4
ILMN_1701025	EPHX1	919.4404	3.2E-11	2.26E-08	31.58637	Up	epoxide hydrolase 1
ILMN_1732071	HIST2H2BE	201.4815	3.52E-11	2.32E-08	31.27832	Up	histone cluster 2 H2B family member e
ILMN_1746158	HOXD11	388.9459	3.53E-11	2.32E-08	31.2676	Up	homeobox D11
ILMN_1736546	SLC16A14	144.273	3.54E-11	2.32E-08	31.26036	Up	solute carrier family 16 member 14
ILMN_1653730	OXCT2	218.2732	3.58E-11	2.32E-08	31.22454	Up	3-oxoacid CoA-transferase 2
ILMN_3238785	SNHG9	1930.849	4.88E-11	3.04E-08	30.2443	Up	small nucleolar RNA host gene 9
ILMN_2354953	NQO1	429.3587	5.11E-11	3.14E-08	30.10341	Up	NAD(P)H quinone dehydrogenase 1
ILMN_3245869	SMIM4	1359.116	5.62E-11	3.3E-08	29.81239	Up	small integral membrane protein 4
ILMN_1717639	SIK1	288.3458	5.99E-11	3.42E-08	29.61529	Up	salt inducible kinase 1
ILMN_3209070	ASS1	534.5687	7.75E-11	4.17E-08	28.84285	Up	argininosuccinate synthase 1
ILMN_1659047	HIST2H2AA4	966.365	7.95E-11	4.18E-08	28.77046	Up	histone cluster 2 H2A family member a4
ILMN_1727288	EVPL	140.8534	8.06E-11	4.18E-08	28.72663	Up	envoplakin
ILMN_1696702	NEO1	401.6901	8.13E-11	4.18E-08	28.70408	Up	neogenin 1
ILMN_2216815	MAP7	144.4914	8.24E-11	4.19E-08	28.6637	Up	microtubule associated protein 7
ILMN_1807016	LHX2	274.3183	8.76E-11	4.4E-08	28.4845	Up	LIM homeobox 2
ILMN_1663490	ZNF541	140.7029	8.83E-11	4.4E-08	28.46151	Up	zinc finger protein 541
ILMN_3217276	FAM86B3P	254.7998	9.11E-11	4.46E-08	28.36964	Up	family with sequence similarity 86, member A pseudogene
ILMN_3243366	KIAA1211L	1095.317	9.15E-11	4.46E-08	28.35652	Up	KIAA1211 like
ILMN_1792689	HIST1H2AC	558.7353	9.45E-11	4.56E-08	28.26278	Up	histone cluster 1 H2A family member c
ILMN_1905548	MBNL3	152.1192	9.77E-11	4.62E-08	28.16663	Up	muscleblind like splicing regulator 3
ILMN_1732923	SIPA1L2	436.353	9.77E-11	4.62E-08	28.16477	Up	signal induced proliferation associated 1 like 2
ILMN_1722309	ENDOG	585.0578	1.07E-10	4.99E-08	27.90677	Up	endonuclease G
ILMN_2096784	TFAP2C	1464.479	1.09E-10	4.99E-08	27.84816	Up	transcription factor AP-2 gamma
ILMN_1739001	TACSTD2	1050.494	1.1E-10	4.99E-08	27.83311	Up	tumor associated calcium signal transducer 2
ILMN_3309634	PSORS1C3	307.1606	1.14E-10	5.12E-08	27.72946	Up	psoriasis susceptibility 1 candidate 3 (non-protein coding)
ILMN_1807277	IFI30	888.1854	1.43E-10	6.14E-08	27.08778	Up	IFI30, lysosomalthiolreductase
ILMN_1804735	CBS	3304.31	1.46E-10	6.19E-08	27.02461	Up	cystathionine-beta-synthase
ILMN_1755638	TAF7L	481.1609	1.46E-10	6.19E-08	27.01788	Up	TATA-box binding protein associated factor 7 like
ILMN_1697703	HPDL	241.4089	1.48E-10	6.2E-08	26.98615	Up	4-hydroxyphenylpyruvate dioxygenase like
ILMN_1769849	HORMAD1	191.0888	1.57E-10	6.44E-08	26.83275	Up	HORMA domain containing 1
ILMN_3242900	HIST2H2AA4	717.8713	1.63E-10	6.65E-08	26.72263	Up	histone cluster 2 H2A family member a4
ILMN_2359945	CES1	135.4178	1.72E-10	6.77E-08	26.57534	Up	carboxylesterase 1
ILMN_1793410	SNTB1	177.4438	1.73E-10	6.77E-08	26.55954	Up	syntrophin beta 1

ILMN_1811328	DPP7	240.9712	1.73E-10	6.77E-08	26.55757	Up	dipeptidyl peptidase 7
ILMN_3241081	LINC01315	210.6253	1.8E-10	7E-08	26.44467	Up	long intergenic non-protein coding RNA 1315
ILMN_1776121	KIAA1211L	2740.506	1.85E-10	7.13E-08	26.37242	Up	KIAA1211 like
ILMN_1675677	TMPRSS3	168.2018	1.92E-10	7.31E-08	26.27968	Up	transmembrane protease, serine 3
ILMN_1699491	DMRTA2	128.4503	2.09E-10	7.74E-08	26.04054	Up	DMRT like family A2
ILMN_1728885	KIAA1644	670.8563	2.28E-10	8.29E-08	25.81602	Up	KIAA1644
ILMN_2203729	HCG4	90.11997	2.34E-10	8.46E-08	25.74428	Up	HLA complex group 4 (non-protein coding)
ILMN_1715189	LHX6	218.5718	2.37E-10	8.49E-08	25.71315	Up	LIM homeobox 6
ILMN_2411781	RYR1	146.2488	2.7E-10	9.47E-08	25.36737	Up	ryanodine receptor 1
ILMN_1751328	FAM83H	829.7031	3E-10	1.02E-07	25.09296	Up	family with sequence similarity 83 member H
ILMN_1800317	WNT5A	1789.724	3.01E-10	1.02E-07	25.08604	Up	Wnt family member 5A
ILMN_1739325	LOC284023	389.2972	3.09E-10	1.04E-07	25.01802	Up	uncharacterized LOC284023
ILMN_1675939	IFNGR1	1259.334	3.09E-10	1.04E-07	25.01404	Up	interferon gamma receptor 1
ILMN_1720373	SLC7A5	13098.42	3.16E-10	1.05E-07	24.95682	Up	solute carrier family 7 member 5
ILMN_1668910	CIDEB	164.0721	3.25E-10	1.07E-07	24.88446	Up	cell death-inducing DFFA-like effector b
ILMN_1693192	PI3	622.4863	3.39E-10	1.1E-07	24.77892	Up	peptidase inhibitor 3
ILMN_1687921	JMJD8	2350.822	3.49E-10	1.12E-07	24.70736	Up	jumonji domain containing 8
ILMN_1731745	NINJ2	264.8807	3.58E-10	1.14E-07	24.64349	Up	ninjurin 2
ILMN_1859127	MIR193BHG	125.3924	3.62E-10	1.15E-07	24.61082	Up	MIR193B host gene
ILMN_1765668	IL20RB	392.1992	3.99E-10	1.21E-07	24.36776	Up	interleukin 20 receptor subunit beta
ILMN_3251423	CHDH	138.3649	4.01E-10	1.21E-07	24.35684	Up	choline dehydrogenase
ILMN_1669479	MEST	124.2121	4.29E-10	1.28E-07	24.18692	Up	mesoderm specific transcript
ILMN_1702503	ALDH3A1	219.782	4.33E-10	1.29E-07	24.16258	Up	aldehyde dehydrogenase 3 family member A1
ILMN_1786326	KCTD15	183.198	4.54E-10	1.33E-07	24.04604	Up	potassium channel tetramerization domain containing 15
ILMN_1802780	CD163L1	628.1012	4.73E-10	1.37E-07	23.94248	Up	CD163 molecule like 1
ILMN_1674390	PRKAR1B	318.9957	4.83E-10	1.38E-07	23.89143	Up	protein kinase cAMP-dependent type I regulatory subunit beta
ILMN_1730639	SLC22A15	239.8666	4.85E-10	1.38E-07	23.88096	Up	solute carrier family 22 member 15
ILMN_2382648	MID2	157.1438	4.95E-10	1.4E-07	23.83283	Up	midline 2
ILMN_1694470	FOXL2NB	136.3776	5.24E-10	1.46E-07	23.69058	Up	FOXL2 neighbor
ILMN_1720282	NQO1	11684.04	5.28E-10	1.46E-07	23.673	Up	NAD(P)H quinone dehydrogenase 1
ILMN_1754241	MEIOB	213.8382	5.32E-10	1.46E-07	23.65669	Up	meiosis specific with OB domains
ILMN_1752932	MPZL2	380.4982	5.46E-10	1.47E-07	23.59281	Up	myelin protein zero like 2
ILMN_2395451	ASS1	3792.073	5.57E-10	1.49E-07	23.54376	Up	argininosuccinate synthase 1
ILMN_1691946	FAM173B	390.7175	5.8E-10	1.53E-07	23.44269	Up	family with sequence similarity 173 member B
ILMN_1697268	EMILIN2	347.5199	5.89E-10	1.54E-07	23.40535	Up	elastin microfibrilinterfacer 2
ILMN_1671489	PC	262.2465	6.37E-10	1.61E-07	23.21932	Up	pyruvate carboxylase
ILMN_1675130	NFIC	1263.523	6.43E-10	1.62E-07	23.19734	Up	nuclear factor I C
ILMN_1664303	HTATIP2	1269.879	6.75E-10	1.65E-07	23.081	Up	HIV-1 Tat interactive protein 2
ILMN_1755897	UGT2B7	96.81211	7.1E-10	1.72E-07	22.95849	Up	UDP glucuronosyltransferase family 2 member B7
ILMN_1681780	MKX	454.5425	7.24E-10	1.74E-07	22.9123	Up	mohawkhomeobox
ILMN_1794505	SEM1	892.9933	7.35E-10	1.76E-07	22.87702	Up	SEM1, 26S proteasome complex subunit
ILMN_1714592	CDA	743.5956	7.56E-10	1.79E-07	22.81176	Up	cytidinedeaminase
ILMN_3224926	RBM47	998.0536	7.91E-10	1.85E-07	22.70516	Up	RNA binding motif protein 47

ILMN_1776188	MAP1LC3A	1153.14	7.94E-10	1.85E-07	22.69729	Up	microtubule associated protein 1 light chain 3 alpha
ILMN_1889555	AC016876.1	175.4732	8.07E-10	1.87E-07	22.65808	Up	NA
ILMN_1680624	CREG1	759.1886	8.8E-10	2.01E-07	22.45523	Up	cellular repressor of E1A stimulated genes 1
ILMN_1745607	A2M	556.623	8.96E-10	2.02E-07	22.41347	Up	alpha-2-macroglobulin
ILMN_1667432	HYAL3	124.5279	1.04E-09	2.27E-07	22.08124	Up	hyaluronoglucosaminidase 3
ILMN_2169761	CPNE8	373.8957	1.05E-09	2.29E-07	22.04721	Up	copine 8
ILMN_1729095	PDZD2	427.1761	1.11E-09	2.38E-07	21.92489	Up	PDZ domain containing 2
ILMN_1672536	FBLN1	2242.746	1.11E-09	2.38E-07	21.91696	Up	fibulin 1
ILMN_3234142	LINC00623	258.4497	1.12E-09	2.38E-07	21.8948	Up	long intergenic non-protein coding RNA 623
ILMN_2324561	SLC7A6	647.0188	1.16E-09	2.43E-07	21.82618	Up	solute carrier family 7 member 6
ILMN_2408796	MFSD12	193.7139	1.16E-09	2.43E-07	21.82561	Up	major facilitator superfamily domain containing 12
ILMN_1657347	PODXL2	382.7564	1.21E-09	2.49E-07	21.73602	Up	podocalyxin like 2
ILMN_1770940	CDH1	59.79132	1.21E-09	2.49E-07	21.7292	Up	cadherin 1
ILMN_2326509	CASP1	740.7477	1.22E-09	2.5E-07	21.70595	Up	caspase 1
ILMN_1761474	HNRNPA3	205.8962	1.26E-09	2.56E-07	21.63033	Up	heterogeneous nuclear ribonucleoprotein A3
ILMN_1709227	CCDC84	391.1836	1.31E-09	2.56E-07	21.55803	Up	coiled-coil domain containing 84
ILMN_1706275	C8orf33	557.9455	1.31E-09	2.56E-07	21.55712	Up	chromosome 8 open reading frame 33
ILMN_1789357	DLX3	118.8501	1.31E-09	2.56E-07	21.5566	Up	distal-less homeobox 3
ILMN_1680364	CD109	82.8302	1.33E-09	2.59E-07	21.51711	Up	CD109 molecule
ILMN_1787345	FKBP11	531.5462	1.36E-09	2.61E-07	21.46342	Up	FK506 binding protein 11
ILMN_1718863	KCNK1	134.8217	1.5E-09	2.77E-07	21.24349	Up	potassium two pore domain channel subfamily K member 1
ILMN_1679041	SLC3A2	1076.961	1.55E-09	2.84E-07	21.17386	Up	solute carrier family 3 member 2
ILMN_1726928	TCEA3	609.1932	1.56E-09	2.84E-07	21.15964	Up	transcription elongation factor A3
ILMN_1679194	UGT2B7	255.9061	1.59E-09	2.88E-07	21.11867	Up	UDP glucuronosyltransferase family 2 member B7
ILMN_1771862	TXNDC11	340.2873	1.6E-09	2.88E-07	21.10283	Up	thioredoxin domain containing 11
ILMN_2412336	AKR1C2	5591.446	1.61E-09	2.88E-07	21.0876	Up	aldo-ketoreductase family 1 member C2
ILMN_1769135	DPP7	322.4056	1.67E-09	2.93E-07	21.01824	Up	dipeptidyl peptidase 7
ILMN_1792748	CPS1	3167.282	1.71E-09	2.98E-07	20.96855	Up	carbamoyl-phosphate synthase 1
ILMN_1661599	DDIT4	4519.544	1.82E-09	3.14E-07	20.82442	Up	DNA damage inducible transcript 4
ILMN_1722073	MLXIPL	128.7104	1.88E-09	3.2E-07	20.76136	Up	MLX interacting protein like
ILMN_1756657	TRIM7	77.02271	1.91E-09	3.22E-07	20.72908	Up	tripartite motif containing 7
ILMN_1661695	IRAK3	279.0153	1.92E-09	3.22E-07	20.70926	Up	interleukin 1 receptor associated kinase 3
ILMN_1678493	CHN1	643.9155	1.96E-09	3.25E-07	20.66513	Up	chimerin 1
ILMN_1724194	NPEPL1	546.2156	1.99E-09	3.28E-07	20.63709	Up	aminopeptidase like 1
ILMN_1751904	EDNRB	99.11929	1.99E-09	3.28E-07	20.63084	Up	endothelin receptor type B
ILMN_1815313	EPGN	47.13669	2E-09	3.28E-07	20.62285	Up	epithelial mitogen
ILMN_1796179	HIST1H2BK	4501.228	2.18E-09	3.51E-07	20.4404	Up	histone cluster 1 H2B family member k
ILMN_1712719	MAP7	165.9016	2.19E-09	3.52E-07	20.42724	Up	microtubule associated protein 7
ILMN_2384056	GPER1	139.7793	2.21E-09	3.53E-07	20.40961	Up	G protein-coupled estrogen receptor 1
ILMN_1687757	AKR1C4	3190.923	2.3E-09	3.62E-07	20.33039	Up	aldo-ketoreductase family 1 member C4
ILMN_1725241	GSTK1	1937.501	2.39E-09	3.72E-07	20.2453	Up	glutathione S-transferase kappa 1
ILMN_3249366	JMJD8	1707.023	2.48E-09	3.83E-07	20.16764	Up	jumonji domain containing 8
ILMN_1716832	ACSF3	371.2484	2.49E-09	3.83E-07	20.16411	Up	acyl-CoA synthetase family member 3

ILMN_1785424	ABLIM1	840.3364	2.52E-09	3.88E-07	20.13374	Up	actin binding LIM protein 1
ILMN_1674386	PITX1	2703.667	2.56E-09	3.91E-07	20.10252	Up	paired like homeodomain 1
ILMN_1698912	PDF	133.7145	2.64E-09	3.99E-07	20.03848	Up	peptide deformylase (mitochondrial)
ILMN_1772036	STEAP4	944.1414	2.65E-09	4E-07	20.02772	Up	STEAP4 metalloreductase
ILMN_1665428	GSDMD	422.4371	2.72E-09	4.06E-07	19.97481	Up	gasdermin D
ILMN_1754921	FAM43B	64.42392	2.75E-09	4.07E-07	19.95046	Up	family with sequence similarity 43 member B
ILMN_3246678	NPW	193.2937	2.75E-09	4.07E-07	19.95043	Up	neuropeptide W
ILMN_1751744	ANKLE1	148.7648	2.78E-09	4.09E-07	19.93306	Up	ankyrin repeat and LEM domain containing 1
ILMN_1749096	BCL2L10	79.03967	2.79E-09	4.09E-07	19.92773	Up	BCL2 like 10
ILMN_3195198	KRT17	1917.352	2.89E-09	4.18E-07	19.85422	Up	keratin 17
ILMN_1749081	AUTS2	222.2582	2.91E-09	4.18E-07	19.8357	Up	AUTS2, activator of transcription and developmental regulator
ILMN_2117323	PIK3C2B	177.774	2.93E-09	4.18E-07	19.82289	Up	phosphatidylinositol-4-phosphate 3-kinase catalytic subunit type 2 beta
ILMN_2086222	WDR11	54.8491	2.93E-09	4.18E-07	19.81997	Up	WD repeat domain 11
ILMN_1792538	CD7	170.1743	3.03E-09	4.25E-07	19.75445	Up	CD7 molecule
ILMN_1661743	OLMALINC	765.291	3.04E-09	4.25E-07	19.74601	Up	oligodendrocyte maturation-associated long intergenic non-coding RNA
ILMN_1659273	#N/A	257.6977	3.05E-09	4.25E-07	19.73833	Up	NA
ILMN_1777190	CFD	555.7001	3.12E-09	4.31E-07	19.6952	Up	complement factor D
ILMN_2306540	PDE9A	68.00083	3.2E-09	4.39E-07	19.64373	Up	phosphodiesterase 9A
ILMN_1812559	SLC7A6	768.4103	3.27E-09	4.46E-07	19.59556	Up	solute carrier family 7 member 6
ILMN_1706246	CCT5	331.8291	3.33E-09	4.5E-07	19.56187	Up	chaperonin containing TCP1 subunit 5
ILMN_1733094	STEAP1	359.9111	3.35E-09	4.51E-07	19.54973	Up	STEAP family member 1
ILMN_1680937	HIST1H2BC	139.4643	3.48E-09	4.61E-07	19.47337	Up	histone cluster 1 H2B family member c
ILMN_1717722	PDZD2	70.57032	3.59E-09	4.71E-07	19.40755	Up	PDZ domain containing 2
ILMN_1676712	IGFL2-AS1	458.7655	3.61E-09	4.71E-07	19.39741	Up	IGFL2 antisense RNA 1
ILMN_1714691	HOXD10	412.9056	3.62E-09	4.71E-07	19.39183	Up	homeobox D10
ILMN_1670325	SLC47A1	207.8007	3.64E-09	4.71E-07	19.38164	Up	solute carrier family 47 member 1
ILMN_1793888	SERPINB5	431.3518	3.74E-09	4.8E-07	19.32669	Up	serpin family B member 5
ILMN_2371053	EFNA1	208.457	3.74E-09	4.8E-07	19.32631	Up	ephrin A1
ILMN_2130635	FOXRED2	2983.867	3.8E-09	4.86E-07	19.29605	Up	FAD dependent oxidoreductase domain containing 2
ILMN_1913678	IRAK3	386.7195	3.82E-09	4.86E-07	19.28129	Up	interleukin 1 receptor associated kinase 3
ILMN_1761828	E2F4	1532.259	4.09E-09	5.16E-07	19.14574	Up	E2F transcription factor 4
ILMN_2149971	SPINK6	922.4733	4.25E-09	5.33E-07	19.07228	Up	serine peptidase inhibitor, Kazal type 6
ILMN_3275388	ASS1	918.1933	4.58E-09	5.73E-07	18.92394	Up	argininosuccinate synthase 1
ILMN_1801216	S100P	2618.296	4.64E-09	5.77E-07	18.89735	Up	S100 calcium binding protein P
ILMN_1726554	IREB2	262.769	4.66E-09	5.77E-07	18.88851	Up	iron responsive element binding protein 2
ILMN_1697543	SPINK13	358.476	4.92E-09	6E-07	18.78183	Up	serine peptidase inhibitor, Kazal type 13 (putative)
ILMN_2219002	KRT6A	454.2013	5.01E-09	6.07E-07	18.74535	Up	keratin 6A
ILMN_1812073	ATP6V1B1	119.7905	5.19E-09	6.24E-07	18.67721	Up	ATPase H+ transporting V1 subunit B1
ILMN_2131381	PDE3B	55.09345	5.19E-09	6.24E-07	18.67655	Up	phosphodiesterase 3B
ILMN_1695485	FAM225A	101.6419	5.27E-09	6.25E-07	18.64667	Up	family with sequence similarity 225 member A (non-protein coding)
ILMN_1693338	CYP1B1	6504.41	5.27E-09	6.25E-07	18.64658	Up	cytochrome P450 family 1 subfamily B member 1
ILMN_1786766	SNTB2	519.4613	5.32E-09	6.29E-07	18.62957	Up	syntrophin beta 2

ILMN_1739103	MPZL1	236.508	5.43E-09	6.39E-07	18.59172	Up	myelin protein zero like 1
ILMN_2052373	RAB17	84.77967	5.54E-09	6.48E-07	18.55004	Up	RAB17, member RAS oncogene family
ILMN_1740466	FAM46A	683.6034	5.59E-09	6.5E-07	18.53421	Up	family with sequence similarity 46 member A
ILMN_1691290	CELSR3	686.3474	6.11E-09	7.09E-07	18.3624	Up	cadherin EGF LAG seven-pass G-type receptor 3
ILMN_1815610	SYT12	89.84309	6.17E-09	7.11E-07	18.34527	Up	synaptotagmin 12
ILMN_3248676	CBWD3	139.8018	6.18E-09	7.11E-07	18.34087	Up	COBW domain containing 3
ILMN_1811468	IRX3	1030.947	6.33E-09	7.24E-07	18.2945	Up	iroquoishomeobox 3
ILMN_1775016	MPZL2	56.41249	6.38E-09	7.27E-07	18.28075	Up	myelin protein zero like 2
ILMN_1713829	PTGES	4073.284	6.47E-09	7.34E-07	18.25444	Up	prostaglandin E synthase
ILMN_1698766	PYCARD	66.24673	6.59E-09	7.46E-07	18.2178	Up	PYD and CARD domain containing
ILMN_2112638	SVEP1	557.525	7.03E-09	7.91E-07	18.09485	Up	sushi, von Willebrand factor type A, EGF and pentraxin domain containing 1
ILMN_2225144	EIF4E3	518.8962	7.08E-09	7.93E-07	18.08307	Up	eukaryotic translation initiation factor 4E family member 3
ILMN_1713141	LOC389641	139.6281	7.12E-09	7.93E-07	18.07221	Up	uncharacterized LOC389641
ILMN_1682928	CPVL	339.0591	7.35E-09	8.13E-07	18.01071	Up	carboxypeptidase, vitellogenic like
ILMN_1705247	ACSL5	802.6435	7.5E-09	8.18E-07	17.9734	Up	acyl-CoA synthetase long-chain family member 5
ILMN_1682428	HENMT1	1358.397	7.6E-09	8.27E-07	17.9479	Up	HEN1 methyltransferase homolog 1
ILMN_2082489	PRMT7	343.7705	7.99E-09	8.59E-07	17.85546	Up	protein arginine methyltransferase 7
ILMN_1782050	CEBPD	4374.001	8.04E-09	8.61E-07	17.84271	Up	CCAAT/enhancer binding protein delta
ILMN_2355168	MGST1	4709.026	8.28E-09	8.84E-07	17.79008	Up	microsomal glutathione S-transferase 1
ILMN_1718932	MTRR	975.2755	8.37E-09	8.88E-07	17.76863	Up	5-methyltetrahydrofolate-homocysteine methyltransferasereductase
ILMN_1688722	IL13RA2	355.255	8.46E-09	8.94E-07	17.74869	Up	interleukin 13 receptor subunit alpha 2
ILMN_1750130	GSPT1	5869.098	8.53E-09	8.95E-07	17.73459	Up	G1 to S phase transition 1
ILMN_2173294	THNSL2	119.0042	8.56E-09	8.96E-07	17.72724	Up	threonine synthase like 2
ILMN_2144401	GLB1L2	102.1204	8.81E-09	9.18E-07	17.67427	Up	galactosidase beta 1 like 2
ILMN_1651496	HIST1H2BD	511.086	9.11E-09	9.46E-07	17.61164	Up	histone cluster 1 H2B family member d
ILMN_1689046	RBM47	224.4374	9.23E-09	9.54E-07	17.58835	Up	RNA binding motif protein 47
ILMN_1726743	MRPS30	1624.639	9.89E-09	1E-06	17.46145	Up	mitochondrial ribosomal protein S30
ILMN_1663113	TTLL12	663.4658	9.97E-09	1.01E-06	17.44725	Up	tubulin tyrosine ligase like 12
ILMN_1731165	SLC16A8	44.18811	1.07E-08	1.07E-06	17.32295	Up	solute carrier family 16 member 8
ILMN_1746517	KYNU	3456.751	1.08E-08	1.08E-06	17.29393	Up	kynureninase
ILMN_1806030	PPL	294.0087	1.09E-08	1.08E-06	17.28965	Up	periplakin
ILMN_1727360	MAOB	126.9627	1.1E-08	1.09E-06	17.27407	Up	monoamine oxidase B
ILMN_1800512	HMOX1	452.5494	1.12E-08	1.1E-06	17.22974	Up	hemeoxygenase 1
ILMN_2371055	EFNA1	1705.947	1.15E-08	1.12E-06	17.19421	Up	ephrin A1
ILMN_1703855	SERPINB3	61.57566	1.18E-08	1.15E-06	17.14687	Up	serpin family B member 3
ILMN_1858599	RBM43	60.63259	1.18E-08	1.15E-06	17.14001	Up	RNA binding motif protein 43
ILMN_1754304	SNRNP48	144.0657	1.18E-08	1.15E-06	17.13701	Up	small nuclear ribonucleoprotein U11/U12 subunit 48
ILMN_2399463	VAV3	454.5123	1.19E-08	1.15E-06	17.12671	Up	vav guanine nucleotide exchange factor 3
ILMN_1662147	MANEAL	94.57188	1.23E-08	1.17E-06	17.07269	Up	mannosidaseendo-alpha like
ILMN_1672094	DLX1	950.0269	1.24E-08	1.19E-06	17.05302	Up	distal-less homeobox 1
ILMN_1758623	HIST1H2BD	262.1308	1.25E-08	1.19E-06	17.04503	Up	histone cluster 1 H2B family member d
ILMN_1842582	TTC39C	659.3893	1.3E-08	1.22E-06	16.97008	Up	tetratricopeptide repeat domain 39C

ILMN_1695687	SALL4	132.4573	1.3E-08	1.22E-06	16.96708	Up	spalt like transcription factor 4
ILMN_2197101	DDX19A	997.9422	1.31E-08	1.23E-06	16.94991	Up	DEAD-box helicase 19A
ILMN_1804884	C22orf39	86.68174	1.32E-08	1.24E-06	16.93903	Up	chromosome 22 open reading frame 39
ILMN_2412860	MCM4	679.9877	1.33E-08	1.25E-06	16.92248	Up	minichromosome maintenance complex component 4
ILMN_1718712	FAM217B	459.3664	1.35E-08	1.25E-06	16.90818	Up	family with sequence similarity 217 member B
ILMN_1806667	FRAS1	112.1091	1.36E-08	1.26E-06	16.89137	Up	Fraser extracellular matrix complex subunit 1
ILMN_1710752	NAPRT	394.307	1.38E-08	1.27E-06	16.86729	Up	nicotinatephosphoribosyltransferase
ILMN_1760778	ENG	650.5212	1.43E-08	1.32E-06	16.79567	Up	endoglin
ILMN_1752249	PIEZO1	1852.072	1.48E-08	1.36E-06	16.73463	Up	piezo type mechanosensitive ion channel component 1
ILMN_1707286	PLBD1	246.6455	1.51E-08	1.37E-06	16.7058	Up	phospholipase B domain containing 1
ILMN_1808374	SNTB2	2107.262	1.51E-08	1.37E-06	16.70166	Up	syntrophin beta 2
ILMN_1709039	RPL13	611.7929	1.52E-08	1.38E-06	16.6884	Up	ribosomal protein L13
ILMN_1730082	RPUSD4	714.3106	1.53E-08	1.38E-06	16.67739	Up	RNA pseudouridylate synthase domain containing 4
ILMN_2282077	MIB2	527.9457	1.58E-08	1.4E-06	16.63073	Up	mindbomb E3 ubiquitin protein ligase 2
ILMN_1688642	LAMC3	88.58333	1.66E-08	1.47E-06	16.54063	Up	laminin subunit gamma 3
ILMN_2296843	GCDH	256.4412	1.67E-08	1.47E-06	16.53342	Up	glutaryl-CoA dehydrogenase
ILMN_1799024	VAC14	277.9863	1.68E-08	1.47E-06	16.52016	Up	Vac14, PIKFYVE complex component
ILMN_1806790	ROBO1	79.79226	1.68E-08	1.47E-06	16.51434	Up	roundabout guidance receptor 1
ILMN_1764607	UGT1A7	1479.694	1.69E-08	1.47E-06	16.51205	Up	UDP glucuronosyltransferase family 1 member A7
ILMN_2400759	CPVL	767.5721	1.69E-08	1.48E-06	16.50513	Up	carboxypeptidase, vitellogenic like
ILMN_1715650	#N/A	107.3356	1.71E-08	1.49E-06	16.49124	Up	NA
ILMN_2369580	NPRL3	986.0075	1.73E-08	1.49E-06	16.46592	Up	NPR3 like, GATOR1 complex subunit
ILMN_1655758	#N/A	264.1277	1.74E-08	1.49E-06	16.45664	Up	NA
ILMN_1757387	UCHL1	-2605.73	7.07E-14	6.69E-10	-58.9842	Down	ubiquitin C-terminal hydrolase L1
ILMN_1700690	VAT1	-874.34	1.1E-12	3.15E-09	-44.598	Down	vesicle amine transport 1
ILMN_2150787	HLA-C	-324.64	1.4E-12	3.67E-09	-43.5152	Down	major histocompatibility complex, class I, C
ILMN_1757440	FAM69B	-702.912	1.57E-12	3.8E-09	-43.0048	Down	family with sequence similarity 69 member B
ILMN_1658333	ECM1	-339.364	1.77E-12	3.8E-09	-42.4833	Down	extracellular matrix protein 1
ILMN_1706643	COL6A3	-3559.43	2.17E-12	4.13E-09	-41.6049	Down	collagen type VI alpha 3 chain
ILMN_1660000	SNURF	-852.133	7.1E-12	8.61E-09	-36.8495	Down	SNRPN upstream reading frame
ILMN_1723358	SCARA3	-284.963	7.46E-12	8.61E-09	-36.6637	Down	scavenger receptor class A member 3
ILMN_2413330	TMEM107	-228.562	9.6E-12	1.01E-08	-35.7322	Down	transmembrane protein 107
ILMN_2372082	SNHG14	-1158.86	9.61E-12	1.01E-08	-35.7273	Down	small nucleolar RNA host gene 14
ILMN_2354547	TUSC3	-1027.45	1.06E-11	1.09E-08	-35.3629	Down	tumor suppressor candidate 3
ILMN_3240433	GSTT2B	-345.481	1.26E-11	1.22E-08	-34.7537	Down	glutathione S-transferase theta 2B (gene/pseudogene)
ILMN_1740842	SALL2	-314.981	1.29E-11	1.22E-08	-34.6628	Down	spalt like transcription factor 2
ILMN_1705682	P3H3	-544.709	2.46E-11	1.82E-08	-32.4546	Down	prolyl 3-hydroxylase 3
ILMN_3248906	ZC4H2	-122.475	3.1E-11	2.25E-08	-31.6925	Down	zinc finger C4H2-type containing
ILMN_1678805	POMT2	-217.527	3.2E-11	2.26E-08	-31.5847	Down	protein O-mannosyltransferase 2
ILMN_2327346	SSBP4	-290.318	3.4E-11	2.32E-08	-31.3912	Down	single stranded DNA binding protein 4
ILMN_1771482	KIAA1324	-152.364	3.55E-11	2.32E-08	-31.2486	Down	KIAA1324
ILMN_2058782	IFI27	-7174.44	3.82E-11	2.44E-08	-31.015	Down	interferon alpha inducible protein 27
ILMN_1715647	VANGL2	-416.592	3.98E-11	2.51E-08	-30.8827	Down	VANGL planar cell polarity protein 2

ILMN_1727577	GLI2	-136.207	5.41E-11	3.28E-08	-29.9304	Down	GLI family zinc finger 2
ILMN_1693341	SNRPN	-309.558	5.48E-11	3.28E-08	-29.8882	Down	small nuclear ribonucleoprotein polypeptide N
ILMN_3275575	BCRP5	-216.626	5.65E-11	3.3E-08	-29.7943	Down	breakpoint cluster region pseudogene 5
ILMN_2388070	TMEM44	-362.86	6E-11	3.42E-08	-29.6135	Down	transmembrane protein 44
ILMN_1682099	TNFAIP8L3	-101.805	6.38E-11	3.59E-08	-29.4266	Down	TNF alpha induced protein 8 like 3
ILMN_2348268	IFFO1	-517.434	6.9E-11	3.84E-08	-29.1913	Down	intermediate filament family orphan 1
ILMN_1723978	LGALS1	-12962.5	7.01E-11	3.86E-08	-29.1425	Down	galectin 1
ILMN_3273885	C8orf88	-459.567	7.73E-11	4.17E-08	-28.8508	Down	chromosome 8 open reading frame 88
ILMN_1734190	TCEAL3	-1712.17	8.08E-11	4.18E-08	-28.7207	Down	transcription elongation factor A like 3
ILMN_1758146	SIRPA	-1907.93	1.08E-10	4.99E-08	-27.888	Down	signal regulatory protein alpha
ILMN_2370208	CMTM3	-335.958	1.2E-10	5.36E-08	-27.5729	Down	CKLF like MARVEL transmembrane domain containing 3
ILMN_1730101	GSPT2	-247.201	1.28E-10	5.66E-08	-27.3925	Down	G1 to S phase transition 2
ILMN_2189668	NUDT11	-322.032	1.39E-10	6.09E-08	-27.1637	Down	nudix hydrolase 11
ILMN_2157099	CCNA1	-183.323	1.41E-10	6.11E-08	-27.1286	Down	cyclin A1
ILMN_1687592	WWC3	-182.106	1.52E-10	6.31E-08	-26.9159	Down	WWC family member 3
ILMN_1702835	SH3BGRL	-832.961	1.71E-10	6.77E-08	-26.586	Down	SH3 domain binding glutamate rich protein like
ILMN_1688848	TMEM44	-567.898	1.73E-10	6.77E-08	-26.5621	Down	transmembrane protein 44
ILMN_1666976	PLD3	-817.141	1.98E-10	7.49E-08	-26.1922	Down	phospholipase D family member 3
ILMN_1684554	COL16A1	-535.005	2.01E-10	7.53E-08	-26.1576	Down	collagen type XVI alpha 1 chain
ILMN_1726114	SLC45A3	-128.189	2.08E-10	7.74E-08	-26.0545	Down	solute carrier family 45 member 3
ILMN_1684211	SEC14L2	-123.947	2.25E-10	8.27E-08	-25.8451	Down	SEC14 like lipid binding 2
ILMN_1672102	PTPRB	-98.259	2.65E-10	9.42E-08	-25.4182	Down	protein tyrosine phosphatase, receptor type B
ILMN_1782098	SMO	-190.904	2.67E-10	9.42E-08	-25.4001	Down	smoothened, frizzled class receptor
ILMN_1809467	VAMP5	-331.053	2.82E-10	9.8E-08	-25.2581	Down	vesicle associated membrane protein 5
ILMN_3244457	EHBP1L1	-300.796	2.91E-10	1E-07	-25.1777	Down	EH domain binding protein 1 like 1
ILMN_1764082	MBOAT1	-96.1093	3.22E-10	1.06E-07	-24.9134	Down	membrane bound O-acyltransferase domain containing 1
ILMN_1656537	SNRPN	-770.697	3.39E-10	1.1E-07	-24.778	Down	small nuclear ribonucleoprotein polypeptide N
ILMN_2361862	VLDLR	-308.807	3.82E-10	1.19E-07	-24.4739	Down	very low density lipoprotein receptor
ILMN_1666057	REEP2	-319.204	3.83E-10	1.19E-07	-24.4681	Down	receptor accessory protein 2
ILMN_1681269	DYRK4	-470.241	3.83E-10	1.19E-07	-24.4677	Down	dual specificity tyrosine phosphorylation regulated kinase 4
ILMN_3244117	STMN3	-2030.11	3.89E-10	1.2E-07	-24.4312	Down	stathmin 3
ILMN_2058251	VIM	-11824.3	3.92E-10	1.2E-07	-24.4106	Down	vimentin
ILMN_1780132	PELI2	-115.111	3.92E-10	1.2E-07	-24.4096	Down	pellino E3 ubiquitin protein ligase family member 2
ILMN_1886769	#N/A	-110.631	4.43E-10	1.31E-07	-24.1047	Down	NA
ILMN_1815673	DKK3	-5004.87	4.69E-10	1.37E-07	-23.9636	Down	dickkopf WNT signaling pathway inhibitor 3
ILMN_1674344	PARD6G	-274.374	4.71E-10	1.37E-07	-23.9532	Down	par-6 family cell polarity regulator gamma
ILMN_1702301	DOCK10	-281.868	5.11E-10	1.43E-07	-23.7555	Down	dedicator of cytokinesis 10
ILMN_1766261	SLC2A12	-152.938	5.11E-10	1.43E-07	-23.7538	Down	solute carrier family 2 member 12
ILMN_1718961	BNIP3L	-929.891	5.35E-10	1.46E-07	-23.6428	Down	BCL2 interacting protein 3 like
ILMN_2376263	SMARCA1	-794.443	5.36E-10	1.46E-07	-23.6375	Down	SWI/SNF related, matrix associated, actin dependent regulator of chromatin, subfamily a, member 1
ILMN_1662799	GPSM3	-161.036	5.45E-10	1.47E-07	-23.5956	Down	G-protein signaling modulator 3
ILMN_2205032	MAGEE1	-258.738	5.71E-10	1.52E-07	-23.4845	Down	MAGE family member E1

ILMN_1768260	GAS6	-157.945	5.85E-10	1.54E-07	-23.4249	Down	growth arrest specific 6
ILMN_1730054	GSTT1	-883.186	5.93E-10	1.54E-07	-23.3915	Down	glutathione S-transferase theta 1
ILMN_1654697	ZNF280B	-113.685	6.07E-10	1.57E-07	-23.3351	Down	zinc finger protein 280B
ILMN_1687501	MOXD1	-239.61	6.13E-10	1.57E-07	-23.3123	Down	monooxygenase DBH like 1
ILMN_2373062	RHBDF2	-362.926	6.15E-10	1.57E-07	-23.302	Down	rhomboid 5 homolog 2
ILMN_1808117	C1QL4	-520.083	6.27E-10	1.59E-07	-23.2582	Down	complement C1q like 4
ILMN_1707077	SORT1	-433.425	6.58E-10	1.64E-07	-23.1426	Down	sortilin 1
ILMN_1821517	#N/A	-9827.96	6.65E-10	1.64E-07	-23.1166	Down	NA
ILMN_1773940	GPR161	-82.8194	6.65E-10	1.64E-07	-23.1138	Down	G protein-coupled receptor 161
ILMN_1735996	NOX4	-95.3751	6.66E-10	1.64E-07	-23.1125	Down	NADPH oxidase 4
ILMN_1719170	METTL27	-208.011	7E-10	1.71E-07	-22.9947	Down	methyltransferase like 27
ILMN_1756469	GAMT	-980.58	7.21E-10	1.74E-07	-22.9249	Down	guanidinoacetate N-methyltransferase
ILMN_2165753	HLA-A	-1229.57	7.4E-10	1.76E-07	-22.8627	Down	major histocompatibility complex, class I, A
ILMN_1750338	PROSER2	-241.449	7.88E-10	1.85E-07	-22.7133	Down	proline and serine rich 2
ILMN_1668426	SPESP1	-237.135	8.76E-10	2.01E-07	-22.4675	Down	sperm equatorial segment protein 1
ILMN_1803367	MECOM	-337.391	8.79E-10	2.01E-07	-22.4572	Down	MDS1 and EVI1 complex locus
ILMN_1699829	CTGF	-9206.64	8.85E-10	2.01E-07	-22.4416	Down	connective tissue growth factor
ILMN_2063168	MALL	-118.864	8.89E-10	2.01E-07	-22.431	Down	mal, T-cell differentiation protein like
ILMN_1735014	KLF6	-1735.15	9.02E-10	2.02E-07	-22.3975	Down	Kruppel like factor 6
ILMN_1739450	NFE2L1	-451.685	9.36E-10	2.09E-07	-22.3138	Down	nuclear factor, erythroid 2 like 1
ILMN_1784749	GAS6	-2564.1	9.4E-10	2.09E-07	-22.3031	Down	growth arrest specific 6
ILMN_1809963	RSAD1	-157.871	9.49E-10	2.1E-07	-22.2815	Down	radical S-adenosyl methionine domain containing 1
ILMN_1798458	KAZN	-82.5401	9.64E-10	2.12E-07	-22.246	Down	kazrin, periplakin interacting protein
ILMN_2185845	BRSK1	-271.322	1.07E-09	2.33E-07	-21.9975	Down	BR serine/threonine kinase 1
ILMN_1694589	PAQR8	-135.064	1.11E-09	2.38E-07	-21.9229	Down	progestin and adipoQ receptor family member 8
ILMN_2343048	ABCB9	-324.118	1.12E-09	2.38E-07	-21.9087	Down	ATP binding cassette subfamily B member 9
ILMN_3203444	CERCAM	-1228.14	1.14E-09	2.4E-07	-21.8678	Down	cerebral endothelial cell adhesion molecule
ILMN_3238326	RNF144A	-133.043	1.17E-09	2.43E-07	-21.8091	Down	ring finger protein 144A
ILMN_2049536	TRPV2	-152.22	1.21E-09	2.49E-07	-21.7294	Down	transient receptor potential cation channel subfamily V member 2
ILMN_1695299	PDLIM3	-205.983	1.26E-09	2.56E-07	-21.6379	Down	PDZ and LIM domain 3
ILMN_1742866	F2R	-493.159	1.27E-09	2.56E-07	-21.6236	Down	coagulation factor II thrombin receptor
ILMN_1709795	RAC2	-2918.37	1.27E-09	2.56E-07	-21.6148	Down	ras-related C3 botulinum toxin substrate 2 (rho family, small GTP binding protein Rac2)
ILMN_2188521	NECTIN3	-643.543	1.29E-09	2.56E-07	-21.5859	Down	nectin cell adhesion molecule 3
ILMN_2413779	SEZ6L2	-630.432	1.29E-09	2.56E-07	-21.5841	Down	seizure related 6 homolog like 2
ILMN_2145280	DEF6	-109.111	1.29E-09	2.56E-07	-21.5761	Down	DEF6, guanine nucleotide exchange factor
ILMN_2145670	TNC	-936.58	1.31E-09	2.57E-07	-21.5464	Down	tenascin C
ILMN_1777644	INPP5J	-143.3	1.36E-09	2.61E-07	-21.4658	Down	inositol polyphosphate-5-phosphatase J
ILMN_1808707	FSCN1	-3367.34	1.37E-09	2.61E-07	-21.456	Down	fascin actin-bundling protein 1
ILMN_1775762	GNAI2	-1597.52	1.37E-09	2.61E-07	-21.4545	Down	G protein subunit alpha i2
ILMN_1740319	IFI27L2	-777.466	1.37E-09	2.61E-07	-21.4494	Down	interferon alpha inducible protein 27 like 2
ILMN_2367707	PKN1	-406.974	1.39E-09	2.64E-07	-21.4202	Down	protein kinase N1
ILMN_1777660	RNF144A	-186.999	1.42E-09	2.69E-07	-21.3655	Down	ring finger protein 144A
ILMN_1783182	FBN1	-157.123	1.43E-09	2.7E-07	-21.3496	Down	fibrillin 1

ILMN_1730572	HNRNPDL	-338.214	1.44E-09	2.7E-07	-21.3408	Down	heterogeneous nuclear ribonucleoprotein D like
ILMN_1810560	NUPR1	-337.842	1.45E-09	2.72E-07	-21.3205	Down	nuclear protein 1, transcriptional regulator
ILMN_1655614	DSP	-558.855	1.46E-09	2.72E-07	-21.3026	Down	desmoplakin
ILMN_1762255	GSTM1	-146.795	1.46E-09	2.72E-07	-21.302	Down	glutathione S-transferase mu 1
ILMN_2082209	TOX2	-2597.64	1.49E-09	2.76E-07	-21.2573	Down	TOX high mobility group box family member 2
ILMN_1709307	GPSM1	-1494.54	1.55E-09	2.84E-07	-21.1781	Down	G-protein signaling modulator 1
ILMN_1808238	RBPMS2	-1185.07	1.57E-09	2.85E-07	-21.1466	Down	RNA binding protein with multiple splicing 2
ILMN_3244611	MXRA7	-380.188	1.61E-09	2.88E-07	-21.0927	Down	matrix remodeling associated 7
ILMN_1762678	NMT1	-184.889	1.62E-09	2.89E-07	-21.0782	Down	N-myristoyltransferase 1
ILMN_1722820	KDELR3	-866.757	1.65E-09	2.93E-07	-21.0388	Down	KDEL endoplasmic reticulum protein retention receptor 3
ILMN_1687301	VCAN	-697.606	1.66E-09	2.93E-07	-21.0223	Down	versican
ILMN_1801377	SLC29A4	-291.176	1.69E-09	2.96E-07	-20.9903	Down	solute carrier family 29 member 4
ILMN_2372974	SIRPA	-224.17	1.78E-09	3.09E-07	-20.8772	Down	signal regulatory protein alpha
ILMN_1765109	TNFRSF25	-293.448	1.81E-09	3.14E-07	-20.8373	Down	TNF receptor superfamily member 25
ILMN_3245057	ASAP1	-1863.21	1.82E-09	3.14E-07	-20.8312	Down	ArfGAP with SH3 domain, ankyrin repeat and PH domain 1
ILMN_1809850	RCN3	-249.906	1.86E-09	3.19E-07	-20.7788	Down	reticulocalbin 3
ILMN_2398159	DKK3	-5678.5	1.88E-09	3.2E-07	-20.7604	Down	dickkopf WNT signaling pathway inhibitor 3
ILMN_3220888	RPS6	-2849.83	1.91E-09	3.22E-07	-20.7244	Down	ribosomal protein S6
ILMN_1795778	P4HA2	-1714.8	1.92E-09	3.22E-07	-20.7106	Down	prolyl 4-hydroxylase subunit alpha 2
ILMN_1747217	C15orf41	-140.81	1.93E-09	3.22E-07	-20.7002	Down	chromosome 15 open reading frame 41
ILMN_1788250	LDOC1	-1166.74	1.93E-09	3.22E-07	-20.6997	Down	LDOC1, regulator of NFKB signaling
ILMN_1694174	TRIM68	-274.772	1.96E-09	3.25E-07	-20.669	Down	tripartite motif containing 68
ILMN_1705035	FBXL7	-100.197	2.01E-09	3.29E-07	-20.6095	Down	F-box and leucine rich repeat protein 7
ILMN_3191922	KRT8P41	-457.889	2.03E-09	3.3E-07	-20.5928	Down	keratin 8 pseudogene 41
ILMN_1808765	ZNF25	-210.719	2.11E-09	3.42E-07	-20.5085	Down	zinc finger protein 25
ILMN_2307861	COL6A3	-1651.81	2.16E-09	3.49E-07	-20.4572	Down	collagen type VI alpha 3 chain
ILMN_1687508	ALDH7A1	-2486.97	2.22E-09	3.53E-07	-20.406	Down	aldehyde dehydrogenase 7 family member A1
ILMN_1653203	EFEMP2	-1002.4	2.23E-09	3.55E-07	-20.3903	Down	EGF containing fibulin like extracellular matrix protein 2
ILMN_3284584	RPL10	-3174.97	2.25E-09	3.57E-07	-20.3699	Down	ribosomal protein L10
ILMN_1775708	SLC2A3	-1246.56	2.33E-09	3.66E-07	-20.2966	Down	solute carrier family 2 member 3
ILMN_1712413	RPL39L	-1368.54	2.34E-09	3.66E-07	-20.2927	Down	ribosomal protein L39 like
ILMN_3246895	ACTBL2	-87.3228	2.35E-09	3.66E-07	-20.2861	Down	actin, beta like 2
ILMN_1674620	SGCE	-963.156	2.46E-09	3.82E-07	-20.183	Down	sarcoglycan epsilon
ILMN_1671292	LOC390705	-108.385	2.56E-09	3.91E-07	-20.1041	Down	protein phosphatase 2 regulatory subunit B", beta pseudogene
ILMN_2366463	FN1	-373.595	2.62E-09	3.98E-07	-20.0587	Down	fibronectin 1
ILMN_3239445	ZBTB42	-186.788	2.62E-09	3.98E-07	-20.0528	Down	zinc finger and BTB domain containing 42
ILMN_2108735	EEF1A2	-3122.85	2.71E-09	4.06E-07	-19.9808	Down	eukaryotic translation elongation factor 1 alpha 2
ILMN_2054607	CYP4V2	-102.137	2.72E-09	4.06E-07	-19.9799	Down	cytochrome P450 family 4 subfamily V member 2
ILMN_1753370	ABTB2	-113.649	2.75E-09	4.07E-07	-19.9542	Down	ankyrin repeat and BTB domain containing 2
ILMN_1688318	FRG1BP	-573.48	2.79E-09	4.09E-07	-19.9232	Down	FSHD region gene 1 family member B, pseudogene
ILMN_1746378	GHDC	-208.157	2.8E-09	4.09E-07	-19.9177	Down	GH3 domain containing
ILMN_1711606	PRDX5	-3561.51	2.81E-09	4.09E-07	-19.9095	Down	peroxiredoxin 5
ILMN_3247023	CASC15	-39.126	2.86E-09	4.15E-07	-19.8725	Down	cancer susceptibility 15 (non-protein coding)

ILMN_1662587	PNPLA7	-96.6812	2.91E-09	4.18E-07	-19.8349	Down	patatin like phospholipase domain containing 7
ILMN_1881514	LOC90768	-94.7969	2.94E-09	4.18E-07	-19.8188	Down	uncharacterized LOC90768
ILMN_1671568	ECHDC2	-592.365	2.95E-09	4.19E-07	-19.8101	Down	enoyl-CoA hydratase domain containing 2
ILMN_1786108	TUT1	-63.6409	2.99E-09	4.23E-07	-19.7821	Down	terminal uridylyltransferase 1, U6 snRNA-specific
ILMN_1745501	DNALI1	-119.383	2.99E-09	4.23E-07	-19.7781	Down	dynein axonemal light intermediate chain 1
ILMN_1802397	GNA11	-701.272	3.02E-09	4.25E-07	-19.7611	Down	G protein subunit alpha 11
ILMN_1708143	RTL8C	-1102.58	3.05E-09	4.25E-07	-19.7434	Down	retrotransposon Gag like 8C
ILMN_1795342	MLPH	-761.4	3.1E-09	4.31E-07	-19.705	Down	melanophilin
ILMN_2325506	BCAS4	-598.177	3.17E-09	4.38E-07	-19.6595	Down	breast carcinoma amplified sequence 4
ILMN_1701998	AFAP1	-916.583	3.2E-09	4.39E-07	-19.6411	Down	actin filament associated protein 1
ILMN_1798496	HOXB8	-278.962	3.22E-09	4.41E-07	-19.6278	Down	homeobox B8
ILMN_1654109	EGFLAM	-252.238	3.3E-09	4.48E-07	-19.5798	Down	EGF like, fibronectin type III and laminin G domains
ILMN_1669617	GRB10	-376.49	3.3E-09	4.48E-07	-19.578	Down	growth factor receptor bound protein 10
ILMN_2176251	MGME1	-597.07	3.39E-09	4.55E-07	-19.5275	Down	mitochondrial genome maintenance exonuclease 1
ILMN_1772824	WNT5B	-610.971	3.41E-09	4.55E-07	-19.5156	Down	Wnt family member 5B
ILMN_1692731	TTYH3	-484.956	3.41E-09	4.55E-07	-19.5152	Down	tweety family member 3
ILMN_1769575	JAM3	-601.969	3.44E-09	4.58E-07	-19.4973	Down	junctional adhesion molecule 3
ILMN_1703374	NAV1	-360.237	3.45E-09	4.59E-07	-19.4874	Down	neuron navigator 1
ILMN_2323801	MOCS1	-301.288	3.51E-09	4.63E-07	-19.4566	Down	molybdenum cofactor synthesis 1
ILMN_1692398	CNTNAP1	-705.228	3.6E-09	4.71E-07	-19.4015	Down	contactin associated protein 1
ILMN_2077952	GALNT16	-116.113	3.61E-09	4.71E-07	-19.396	Down	polypeptide N-acetylgalactosaminyltransferase 16
ILMN_2098743	ACOT13	-739.646	3.64E-09	4.71E-07	-19.3817	Down	acyl-CoA thioesterase 13
ILMN_1712257	PPP2R3B	-158.5	3.64E-09	4.71E-07	-19.3802	Down	protein phosphatase 2 regulatory subunit B''beta
ILMN_2059689	TMEM54	-352.927	3.68E-09	4.74E-07	-19.3601	Down	transmembrane protein 54
ILMN_1714444	KLF12	-115.691	3.81E-09	4.86E-07	-19.2897	Down	Kruppel like factor 12
ILMN_3237946	PXDN	-8497.98	3.86E-09	4.9E-07	-19.2616	Down	peroxidasin
ILMN_1749868	FAM171A1	-1960.65	4.09E-09	5.16E-07	-19.1458	Down	family with sequence similarity 171 member A1
ILMN_1773855	CPT1C	-323.777	4.15E-09	5.22E-07	-19.1182	Down	carnitinepalmitoyltransferase 1C
ILMN_2232478	APOBEC3G	-99.305	4.6E-09	5.74E-07	-18.9149	Down	apolipoprotein B mRNA editing enzyme catalytic subunit 3G
ILMN_1676448	WDFY1	-1179.6	4.65E-09	5.77E-07	-18.8932	Down	WD repeat and FYVE domain containing 1
ILMN_1675268	LRP4	-76.4727	4.76E-09	5.87E-07	-18.8489	Down	LDL receptor related protein 4
ILMN_2079786	NUAK1	-591.688	4.77E-09	5.88E-07	-18.8407	Down	NUAK family kinase 1
ILMN_1701173	KCNK6	-132.216	4.84E-09	5.94E-07	-18.8158	Down	potassium two pore domain channel subfamily K member 6
ILMN_1764158	NLGN2	-675.914	4.91E-09	6E-07	-18.7871	Down	neuroligin 2
ILMN_1725366	SLC27A5	-144.973	4.91E-09	6E-07	-18.7842	Down	solute carrier family 27 member 5
ILMN_2311020	DNAJC12	-139.863	4.98E-09	6.05E-07	-18.7595	Down	DnaJ heat shock protein family (Hsp40) member C12
ILMN_1910180	NRP2	-60.9845	5E-09	6.06E-07	-18.7512	Down	neuropilin 2
ILMN_1731640	TMSB15B	-421.994	5.04E-09	6.08E-07	-18.7353	Down	thymosin beta 15B
ILMN_1709044	TGIF2	-438.635	5.21E-09	6.24E-07	-18.671	Down	TGFB induced factor homeobox 2
ILMN_3274711	RPL26	-371.55	5.23E-09	6.25E-07	-18.6632	Down	ribosomal protein L26
ILMN_1745329	PRR14	-1146.72	5.25E-09	6.25E-07	-18.6564	Down	proline rich 14
ILMN_1698733	CNIH2	-140.329	5.34E-09	6.3E-07	-18.6226	Down	cornichon family AMPA receptor auxiliary protein 2
ILMN_1705570	H2AFY2	-1051.54	5.49E-09	6.44E-07	-18.5696	Down	H2A histone family member Y2

ILMN_1790891	CKAP4	-2745.85	5.5E-09	6.44E-07	-18.5647	Down	cytoskeleton associated protein 4
ILMN_1755024	IKBKE	-121.533	5.57E-09	6.49E-07	-18.5424	Down	inhibitor of nuclear factor kappa B kinase subunit epsilon
ILMN_2131523	SACS	-416.758	6.17E-09	7.11E-07	-18.3428	Down	sacsin molecular chaperone
ILMN_3283032	CERCAM	-362.418	6.27E-09	7.2E-07	-18.3122	Down	cerebral endothelial cell adhesion molecule
ILMN_3245707	RIMKLB	-139.442	6.32E-09	7.24E-07	-18.2967	Down	ribosomal modification protein rimK like family member B
ILMN_2372200	ZNF586	-130.696	6.46E-09	7.34E-07	-18.256	Down	zinc finger protein 586
ILMN_2413780	SEZ6L2	-174.966	6.75E-09	7.63E-07	-18.1717	Down	seizure related 6 homolog like 2
ILMN_1652407	ZMYND8	-278.215	7.02E-09	7.91E-07	-18.099	Down	zinc finger MYND-type containing 8
ILMN_1664434	TCF3	-476.58	7.08E-09	7.93E-07	-18.0811	Down	transcription factor 3
ILMN_1906110	RAB18	-126.33	7.1E-09	7.93E-07	-18.0763	Down	RAB18, member RAS oncogene family
ILMN_1776516	ITPKA	-221.461	7.15E-09	7.95E-07	-18.0627	Down	inositol-trisphosphate 3-kinase A
ILMN_1694878	MIER2	-188.352	7.35E-09	8.13E-07	-18.013	Down	MIER family member 2
ILMN_2381697	P4HA2	-883.249	7.39E-09	8.15E-07	-18.0007	Down	prolyl 4-hydroxylase subunit alpha 2
ILMN_1701933	SNCA	-307.679	7.42E-09	8.15E-07	-17.9935	Down	synuclein alpha
ILMN_1912185	GPC6	-288.793	7.43E-09	8.15E-07	-17.9908	Down	glypican 6
ILMN_3208576	HOXA-AS2	-141.302	7.44E-09	8.15E-07	-17.9881	Down	HOXA cluster antisense RNA 2
ILMN_1681777	SHROOM2	-109.188	7.47E-09	8.17E-07	-17.9809	Down	shroom family member 2
ILMN_2400372	SULT1A2	-166.027	7.62E-09	8.27E-07	-17.9438	Down	sulfotransferase family 1A member 2
ILMN_1683660	EIF3H	-1664.24	7.73E-09	8.37E-07	-17.9183	Down	eukaryotic translation initiation factor 3 subunit H
ILMN_1708029	TMEM8B	-249.091	7.9E-09	8.53E-07	-17.8777	Down	transmembrane protein 8B
ILMN_1779470	ABCG4	-72.723	7.92E-09	8.54E-07	-17.8721	Down	ATP binding cassette subfamily G member 4
ILMN_2062468	IGFBP7	-6580.25	8.01E-09	8.59E-07	-17.851	Down	insulin like growth factor binding protein 7
ILMN_1692077	MXRA7	-381.678	8.33E-09	8.88E-07	-17.7768	Down	matrix remodeling associated 7
ILMN_1787981	MFAP2	-333.928	8.35E-09	8.88E-07	-17.773	Down	microfibrillar associated protein 2
ILMN_1790680	PDE6D	-333.286	8.47E-09	8.94E-07	-17.7464	Down	phosphodiesterase 6D
ILMN_1666727	ZNF586	-269.74	8.49E-09	8.94E-07	-17.742	Down	zinc finger protein 586
ILMN_1880983	CDYL2	-209.249	8.51E-09	8.94E-07	-17.7389	Down	chromodomain Y like 2
ILMN_1828967	#N/A	-65.9558	8.61E-09	9E-07	-17.7155	Down	NA
ILMN_1785405	SLC17A9	-114.495	9.07E-09	9.43E-07	-17.6201	Down	solute carrier family 17 member 9
ILMN_1720241	TRIP12	-663.437	9.13E-09	9.46E-07	-17.6077	Down	thyroid hormone receptor interactor 12
ILMN_1679025	RPS3A	-740.458	9.33E-09	9.62E-07	-17.5689	Down	ribosomal protein S3A
ILMN_3248975	PPP4C	-789.062	9.54E-09	9.81E-07	-17.5284	Down	protein phosphatase 4 catalytic subunit
ILMN_1769299	MTMR11	-439.015	9.6E-09	9.85E-07	-17.5167	Down	myotubularin related protein 11
ILMN_1800733	MANBA	-200.132	9.64E-09	9.87E-07	-17.5084	Down	mannosidase beta
ILMN_1658289	WDR54	-2968.3	9.69E-09	9.91E-07	-17.4985	Down	WD repeat domain 54
ILMN_1811313	SLIT3	-150.14	9.8E-09	9.98E-07	-17.4787	Down	slit guidance ligand 3
ILMN_1808898	NPR2	-255.484	9.81E-09	9.98E-07	-17.4766	Down	natriuretic peptide receptor 2
ILMN_1723467	ITGB1	-9302.85	1.03E-08	1.04E-06	-17.3944	Down	integrin subunit beta 1
ILMN_1681737	TMSB15A	-236.041	1.04E-08	1.05E-06	-17.3708	Down	thymosin beta 15a
ILMN_1752895	STX8	-506.161	1.07E-08	1.07E-06	-17.325	Down	syntaxin 8
ILMN_1763461	ALDH7A1	-2316.96	1.07E-08	1.07E-06	-17.3145	Down	aldehyde dehydrogenase 7 family member A1
ILMN_1685433	COL8A1	-691.842	1.09E-08	1.08E-06	-17.2893	Down	collagen type VIII alpha 1 chain
ILMN_1664878	NLRP2	-120.823	1.1E-08	1.09E-06	-17.2666	Down	NLR family pyrin domain containing 2

ILMN_1692865	VPS37D	-174.23	1.11E-08	1.1E-06	-17.2543	Down	VPS37D, ESCRT-I subunit
ILMN_1657836	PLEKHG2	-86.4437	1.11E-08	1.1E-06	-17.2497	Down	pleckstrin homology and RhoGEF domain containing G2
ILMN_1718303	NECTIN2	-436.436	1.11E-08	1.1E-06	-17.2463	Down	nectin cell adhesion molecule 2
ILMN_2129234	TMEM47	-199.699	1.12E-08	1.1E-06	-17.2346	Down	transmembrane protein 47
ILMN_1726417	MRPL33	-1726.23	1.13E-08	1.1E-06	-17.2272	Down	mitochondrial ribosomal protein L33
ILMN_1789599	NBL1	-1115.42	1.19E-08	1.15E-06	-17.1305	Down	neuroblastoma 1, DAN family BMP antagonist
ILMN_1765796	ENO2	-1527.12	1.19E-08	1.15E-06	-17.1218	Down	enolase 2
ILMN_2072178	ECHDC3	-95.7285	1.21E-08	1.16E-06	-17.0997	Down	enoyl-CoA hydratase domain containing 3
ILMN_1805007	SEMA4F	-388.039	1.22E-08	1.17E-06	-17.0874	Down	ssemaphorin 4F
ILMN_1810420	DYSF	-275.438	1.22E-08	1.17E-06	-17.0757	Down	dysferlin
ILMN_1763941	LRRC49	-150.912	1.25E-08	1.19E-06	-17.0413	Down	leucine rich repeat containing 49
ILMN_1741356	PRICKLE1	-323.773	1.25E-08	1.19E-06	-17.0323	Down	prickle planar cell polarity protein 1
ILMN_1666503	DENND2A	-161.214	1.26E-08	1.19E-06	-17.0292	Down	DENN domain containing 2A
ILMN_1756071	MFGE8	-4301.63	1.28E-08	1.21E-06	-17	Down	milk fat globule-EGF factor 8 protein
ILMN_1654946	ZSCAN18	-218.941	1.3E-08	1.22E-06	-16.9676	Down	zinc finger and SCAN domain containing 18
ILMN_1775330	C15orf52	-1571.91	1.33E-08	1.24E-06	-16.9269	Down	chromosome 15 open reading frame 52
ILMN_1678692	MPRIP	-622.494	1.34E-08	1.25E-06	-16.9132	Down	myosin phosphatase Rho interacting protein
ILMN_1741371	TMEM8A	-362.477	1.36E-08	1.26E-06	-16.8889	Down	transmembrane protein 8A
ILMN_1706571	SLC35D2	-144.526	1.4E-08	1.3E-06	-16.8318	Down	solute carrier family 35 member D2
ILMN_1794595	GAMT	-662.402	1.42E-08	1.31E-06	-16.8149	Down	guanidinoacetate N-methyltransferase
ILMN_2355549	GSTT2	-344.202	1.43E-08	1.32E-06	-16.7948	Down	glutathione S-transferase theta 2 (gene/pseudogene)
ILMN_1815141	MTR	-138.348	1.46E-08	1.33E-06	-16.7652	Down	5-methyltetrahydrofolate-homocysteine methyltransferase
ILMN_2386179	ZMYND8	-230.821	1.49E-08	1.36E-06	-16.723	Down	zinc finger MYND-type containing 8
ILMN_1808404	RHBDF1	-712.67	1.5E-08	1.36E-06	-16.7186	Down	rhomboid 5 homolog 1
ILMN_1678353	FARP1	-613.659	1.53E-08	1.38E-06	-16.6759	Down	FERM, ARH/RhoGEF and pleckstrin domain protein 1
ILMN_1728057	SDHAF4	-155.106	1.54E-08	1.39E-06	-16.6696	Down	succinate dehydrogenase complex assembly factor 4
ILMN_1719986	PIK3IP1	-156.167	1.55E-08	1.39E-06	-16.663	Down	phosphoinositide-3-kinase interacting protein 1
ILMN_1699472	ST3GAL3	-136.784	1.57E-08	1.4E-06	-16.6394	Down	ST3 beta-galactoside alpha-2,3-sialyltransferase 3
ILMN_1708987	HSD11B1L	-97.3369	1.57E-08	1.4E-06	-16.6317	Down	hydroxysteroid 11-beta dehydrogenase 1 like
ILMN_2360710	TPM1	-5544.47	1.58E-08	1.4E-06	-16.6292	Down	tropomyosin 1 (alpha)
ILMN_2352023	DSTYK	-474.039	1.61E-08	1.43E-06	-16.5906	Down	dual serine/threonine and tyrosine protein kinase
ILMN_1815346	TMEM136	-227.333	1.63E-08	1.45E-06	-16.5733	Down	transmembrane protein 136
ILMN_2093720	THG1L	-125.789	1.65E-08	1.46E-06	-16.5465	Down	tRNA-histidineguanylyltransferase 1 like
ILMN_2363668	YIF1B	-485.644	1.66E-08	1.47E-06	-16.5376	Down	Yip1 interacting factor homolog B, membrane trafficking protein
ILMN_1749636	DNAJC22	-223.093	1.69E-08	1.47E-06	-16.5124	Down	DnaJ heat shock protein family (Hsp40) member C22
ILMN_1740493	TRAF5	-222.981	1.71E-08	1.49E-06	-16.4894	Down	TNF receptor associated factor 5
ILMN_1809566	ZSCAN16	-181.258	1.71E-08	1.49E-06	-16.4846	Down	zinc finger and SCAN domain containing 16
ILMN_1796923	LOC81691	-135.33	1.72E-08	1.49E-06	-16.4744	Down	exonuclease NEF-sp
ILMN_1769013	ASGR1	-77.4936	1.73E-08	1.49E-06	-16.4656	Down	asialoglycoprotein receptor 1
ILMN_1685339	TPM1	-250.252	1.73E-08	1.49E-06	-16.4651	Down	tropomyosin 1 (alpha)
ILMN_1779558	GAS6	-2654.8	1.73E-08	1.49E-06	-16.4651	Down	growth arrest specific 6
ILMN_1798177	CHURC1	-760.303	1.74E-08	1.49E-06	-16.4571	Down	churchill domain containing 1

N/A - Not Annotated

Table 2. Pathway enrichment analysis of the up regulated differentially expressed genes.

Pathway ID	Pathway Name	LogP	Gene Count	Genes
**BIOCYC**				
142426	urea cycle	3.08138859	2	ASS1,CPS1
782401	pyrimidine deoxyribonucleosides degradation	2.862942799	2	CDA,TYMP
1108771	allopregnanolone biosynthesis	2.690251203	2	AKR1C2,AKR1C4
545337	putrescine degradation III	2.425974194	2	ALDH3A1,MAOB
835392	superpathway of tryptophan utilization	2.197680219	3	CYP1B1,KYNU,MAOB
142141	cysteine biosynthesis/homocysteine degradation (trans-sulfuration)	1.91968774	1	CBS
1108776	hydrogen sulfide biosynthesis (trans-sulfuration)	1.91968774	1	CBS
142435	glutathione-mediated detoxification	1.677249926	2	GSTK1,MGST1
142299	choline degradation	1.62641838	1	CHDH
142431	pyrimidine ribonucleosides degradation	1.452924595	1	CDA
**KEGG**				
673221	Chemical carcinogenesis	10	11	AKR1C2,ALDH3A1,CYP1B1, EPHX1,GSTK1,GSTO2,MGST1, UGT1A10,UGT1A7,UGT1A8, UGT2B7
83032	Drug metabolism - cytochrome P450	10	9	ALDH3A1,GSTK1,GSTO2,MAOB,MGST1,UGT1A10,UGT1A7, UGT1A8,UGT2B7
83031	Metabolism of xenobiotics by cytochrome P450	10	10	ALDH3A1,CYP1B1,EPHX1,GSTK1,GSTO2,MGST1,UGT1A10, UGT1A7,UGT1A8,UGT2B7
83033	Drug metabolism - other enzymes	5.976093317	7	CDA,CES1,TYMP,UGT1A10, UGT1A7,UGT1A8,UGT2B7
82940	Steroid hormone biosynthesis	5.278262272	7	AKR1C2,AKR1C4,CYP1B1, UGT1A10,UGT1A7,UGT1A8, UGT2B7
83021	Porphyrin and chlorophyll metabolism	3.878083085	5	HMOX1,UGT1A10,UGT1A7, UGT1A8,UGT2B7
83122	Systemic lupus erythematosus	3.733288033	8	HIST1H2AC,HIST1H2BC ,HIST1H2BD,HIST1H2BJ, HIST1H2BK,HIST2H2AA3, HIST2H2AA4,HIST2H2BE
997799	Glucuronate pathway (uronate pathway)	3.565440004	4	UGT1A10,UGT1A7,UGT1A8,UGT2B7
82932	Ascorbate and aldarate metabolism	3.565440004	4	UGT1A10,UGT1A7,UGT1A8, UGT2B7
585563	Alcoholism	3.529115138	9	HIST1H2AC,HIST1H2BC, HIST1H2BD,HIST1H2BJ, HIST1H2BK,HIST2H2AA3, HIST2H2AA4,HIST2H2BE,MAOB
**Pathway Interaction Database**				
137961	EPHA2 forward signaling	1.769988299	2	EFNA1,VAV3
137966	E-cadherin signaling events	1.62641838	1	CDH1
138082	Class IB PI3K non-lipid kinase events	1.452924595	1	PDE3B
138045	HIF-1-alpha transcription factor network	1.375496122	3	ADM,ENG,HMOX1
**REACTOME**				
1269864	Packaging Of Telomere Ends	10	8	HIST1H2AC,HIST1H2BC,HIST1H2BD,HIST1H2BJ,HIST1H2BK,HIST2H2AA3,HIST2H2AA4, HIST2H2BE
1269659	RNA Polymerase I Promoter Opening	10	8	HIST1H2AC,HIST1H2BC,HIST1H2BD,HIST1H2BJ,HIST1H2BK,HIST2H2AA3,HIST2H2AA4, HIST2H2BE
1270429	DNA Damage/Telomere Stress Induced Senescence	10	9	HIST1H1C,HIST1H2AC,HIST1H2BC,HIST1H2BD,HIST1H2BJ,HIST1H2BK,HIST2H2AA3,HIST2H2AA4,HIST2H2BE
1269867	Meiotic synapsis	10	9	HIST1H2AC,HIST1H2BC,HIST1H2BD,HIST1H2BJ,HIST1H2BK,HIST2H2AA3,HIST2H2AA4, HIST2H2BE,SYCP2

1269740	DNA methylation	5.975472892	8	HIST1H2AC,HIST1H2BC,HIST1H2BD,HIST1H2BJ,HIST1H2BK,HIST2H2AA3,HIST2H2AA4,HIST2H2BE
1269513	Activated PKN1 stimulates transcription of AR (androgen receptor) regulated genes KLK2 and KLK3	5.874592306	8	HIST1H2AC,HIST1H2BC,HIST1H2BD,HIST1H2BJ,HIST1H2BK,HIST2H2AA3,HIST2H2AA4,HIST2H2BE
1269738	SIRT1 negatively regulates rRNA Expression	5.777065352	8	HIST1H2AC,HIST1H2BC,HIST1H2BD,HIST1H2BJ,HIST1H2BK,HIST2H2AA3,HIST2H2AA4,HIST2H2BE
1269855	Deposition of new CENPA-containing nucleosomes at the centromere	5.636631204	8	HIST1H2AC,HIST1H2BC,HIST1H2BD,HIST1H2BJ,HIST1H2BK,HIST2H2AA3,HIST2H2AA4,HIST2H2BE
1269854	Nucleosome assembly	5.636631204	8	HIST1H2AC,HIST1H2BC,HIST1H2BD,HIST1H2BJ,HIST1H2BK,HIST2H2AA3,HIST2H2AA4,HIST2H2BE
1269735	PRC2 methylates histones and DNA	5.54665253	8	HIST1H2AC,HIST1H2BC,HIST1H2BD,HIST1H2BJ,HIST1H2BK,HIST2H2AA3,HIST2H2AA4,HIST2H2BE
		**GenMAPP**		
MAP00330	Arginine and proline metabolism	1.989499	3	ASS1,CPS1,MAOB
MAP00360	Phenylalanine metabolism	1.820982	2	ALDH3A1,MAOB
MAP00220	Urea cycle and metabolism of amino groups	1.769988	2	ASS1,CPS1
MAP00380	Tryptophan metabolism	1.764206	3	GCDH,KYNU,MAOB
MAP00340	Histidine metabolism	1.634847	2	ALDH3A1,MAOB
MAP00252	Alanine and aspartate metabolism	1.594733	2	ASS1,PC
MAP00260	Glycine serine and threonine metabolism	1.520524	2	CBS,MAOB
MAP00350	Tyrosine metabolism	1.308476	2	ALDH3A1,MAOB
		**MSigDB C2 BIOCARTA (v6.0)**		
M3468	Genes encoding enzymes and their regulators involved in the remodeling of the extracellular matrix	1.6276	7	A2M, CD109, HYAL3, PI3, SERPINB3, SERPINB5, SLPI
		**PantherDB**		
P05914	Nicotine degradation	4.392989178	4	UGT1A10,UGT1A7,UGT1A8,UGT2B7
P02728	Arginine biosynthesis	2.547519644	2	ASS1,CPS1
P02737	Cysteine biosynthesis	1.91968774	1	CBS
P04372	5-Hydroxytryptamine degredation	1.72219682	2	ALDH3A1,MAOB
P02754	Methylcitrate cycle	1.62641838	1	IREB2
P02774	Salvage pyrimidine deoxyribonucleotides	1.452924595	1	CDA
P00009	Axon guidance mediated by netrin	1.308475736	2	ABLIM1,PIK3C2B
		**Pathway Ontology**		
PW:0000050	arginine and proline metabolic	2.72790933	3	ASS1,CPS1,MAOB
PW:0000054	tryptophan metabolic	2.611088865	3	CYP1B1,KYNU,MAOB
PW:0000053	phenylalanine metabolic	2.320219814	2	ALDH3A1,MAOB
PW:0000028	alanine and aspartate metabolic	2.226696687	2	ASS1,PC
PW:0000051	histidine metabolic	2.067129404	2	ALDH3A1,MAOB
PW:0000076	pathway of urea cycle and metabolism of amino groups	1.997955977	2	ASS1,CPS1
PW:0000047	glycine, serine and threonine metabolic	1.934383201	2	CBS,MAOB
PW:0000413	heme catabolic	1.91968774	1	HMOX1
PW:0000378	oxidative stress responses	1.91968774	1	NQO1
PW:0000023	immune response	1.91968774	1	A2M
		**SMPDB**		
SMP00375	Verapamil Pathway	2.332712643	4	EDNRB,KCNK1,SNTB1,SNTB2
SMP00359	Diltiazem Pathway	2.332712643	4	EDNRB,KCNK1,SNTB1,SNTB2
SMP00059	Urea Cycle	2.320219814	2	ASS1,CPS1
SMP00323	Quinidine Pathway	2.021474016	3	KCNK1,SNTB1,SNTB2
SMP00330	Tocainide Pathway	2.021474016	3	KCNK1,SNTB1,SNTB2
SMP00328	Lidocaine (Antiarrhythmic) Pathway	2.021474016	3	KCNK1,SNTB1,SNTB2

SMP00331	Flecainide Pathway	2.021474016	3	KCNK1,SNTB1,SNTB2
SMP00324	Procainamide (Antiarrhythmic) Pathway	2.021474016	3	KCNK1,SNTB1,SNTB2
SMP00327	Phenytoin (Antiarrhythmic) Pathway	2.021474016	3	KCNK1,SNTB1,SNTB2
SMP00325	Disopyramide Pathway	2.021474016	3	KCNK1,SNTB1,SNTB2

Table 3 Pathway enrichment analysis of the down regulated differentially expressed genes

BIOCYC

Pathway ID	Pathway Name	LogP	Gene Count	Genes
142292	creatine biosynthesis	1.879927	1	GAMT
545332	1D-myo-inositol hexakisphosphate biosynthesis II (mammalian)	1.80106	2	INPP5J,ITPKA
545328	D-myo-inositol (1,3,4)-trisphosphate biosynthesis	1.80106	2	INPP5J,ITPKA
142435	glutathione-mediated detoxification	1.603978	2	GSTM1,GSTT1
142299	choline degradation	1.587404	1	ALDH7A1
142362	lysine degradation I (saccharopine pathway)	1.587404	1	ALDH7A1
545325	superpathway of D-myo-inositol (1,4,5)-trisphosphate metabolism	1.561892	2	INPP5J,ITPKA
142159	methionine salvage	1.414162	1	MTR
	**KEGG**			
83069	Cell adhesion molecules (CAMs)	3.940436	9	CNTNAP1,HLA-A, HLA-C, ITGB1,JAM3,NECTIN2, NECTIN3,NLGN2,VCAN
83065	Axon guidance	2.688949	8	GNAI2,ITGB1,PARD6G,RAC2, SEMA4F,SLIT3,SMO,WNT5B
673221	Chemical carcinogenesis	2.369043	5	GSTM1,GSTT1,GSTT2,GSTT2B, SULT1A2
82973	Glutathione metabolism	2.278173	4	GSTM1,GSTT1,GSTT2,GSTT2B
83105	Pathways in cancer	2.259846	12	CCNA1,F2R,FN1,GLI2,GNA11,GNAI2,ITGB1,MECOM,RAC2, SMO,TRAF5,WNT5B
83032	Drug metabolism - cytochrome P450	1.885209	4	GSTM1,GSTT1,GSTT2,GSTT2B
83070	Adherens junction	1.843833	4	NECTIN2,NECTIN3,PTPRB,RAC2
1404797	Platinum drug resistance	1.823672	4	GSTM1,GSTT1,GSTT2,GSTT2B
83031	Metabolism of xenobiotics by cytochrome P450	1.803849	4	GSTM1,GSTT1,GSTT2,GSTT2B
83068	ECM-receptor interaction	1.656392	4	COL6A3,FN1,ITGB1,TNC
	**Pathway Interaction Database**			
138064	Syndecan-4-mediated signaling events	2.128333	3	FN1,ITGB1,TNC
137945	amb2 Integrin signaling	1.612626	3	CTGF,FN1,JAM3
137931	Sphingosine 1-phosphate (S1P) pathway	1.522093	2	GNA11,GNAI2
137950	Signaling events mediated by the Hedgehog family	1.484364	2	GLI2,SMO
137996	VEGFR3 signaling in lymphatic endothelium	1.448515	2	FN1,ITGB1
138059	S1P2 pathway	1.414382	2	GNA11,GNAI2
137965	a4b1 and a4b7 Integrin signaling	1.414162	1	ITGB1
138054	Nectin adhesion pathway	1.381821	2	NECTIN2,NECTIN3
	**REACTOME**			
1270244	Extracellular matrix organization	4.464123	14	COL16A1,COL6A3,COL8A1, EFEMP2,FBN1,FN1,ITGB1, jAM3,LRP4,MFAP2,P3H3,P4HA2,TNC,VCAN
1270260	Integrin cell surface interactions	3.604255	6	COL16A1,FBN1,FN1,ITGB1, AM3,TNC
1268678	Translation	2.850402	8	EEF1A2,EIF3H,GSPT2,RPL10, RPL26,RPL39L,RPS3A,RPS6
1268692	Eukaryotic Translation Termination	2.782871	6	GSPT2,RPL10,RPL26,RPL39L, RPS3A,RPS6

1268690	Eukaryotic Translation Elongation	2.760047	6	EEF1A2,RPL10,RPL26,RPL39L, RPS3A,RPS6
1270213	Glutathione conjugation	2.756259	4	GSTM1,GSTT1,GSTT2,GSTT2B
1269718	Nonsense Mediated Decay (NMD) independent of the Exon Junction Complex (EJC)	2.715247	6	GSPT2,RPL10,RPL26,RPL39L, RPS3A,RPS6
1270251	Elastic fibre formation	2.676954	4	EFEMP2,FBN1,ITGB1,MFAP2
1270246	Collagen biosynthesis and modifying enzymes	2.668023	5	COL16A1,COL6A3,COL8A1,P3H3,P4HA2
1270250	Fibronectin matrix formation	2.612799	2	FN1,ITGB1
**GenMAPP**				
MAP00480	Glutathione metabolism	2.5564	3	GSTM1, GSTT1, GSTT2
**MSigDB C2 BIOCARTA (v6.0)**				
M5884	Ensemble of genes encoding core extracellular matrix including ECM glycoproteins, collagens and proteoglycans	10	17	COL16A1,COL6A3,COL8A1,CTGF,ECM1,EFEMP2,EGFLAM,FBN1,FN1,GAS6,IGFBP7,MFAP2,MFGE8,PXDN,SLIT3,TNC,VCAN
M3008	Genes encoding structural ECM glycoproteins	5.787088	13	CTGF,ECM1,EFEMP2,EGFLAM,FBN1,FN1,GAS6,IGFBP7,MFAP2,MFGE8,PXDN,SLIT3,TNC
M5889	Ensemble of genes encoding extracellular matrix and extracellular matrix-associated proteins	2.77273	25	C1QL4,COL16A1,COL6A3,COL8A1,CTGF,ECM1,EFEMP2,EGFLAM,FBN1,FN1,GAS6,GPC6,IGFBP7,LGALS1,MFAP2,MFGE8,P3H3,P4HA2,PIK3IP1,PXDN,SEMA4F,SLIT3,TNC,VCAN,WNT5B
M11792	Sonic Hedgehog (Shh) Pathway	1.746755	2	GLI2,SMO
M3005	Genes encoding collagen proteins	1.712267	3	COL16A1,COL6A3,COL8A1
M16518	Wnt/Ca2+/cyclic GMP signaling.	1.561892	3	ITPKA,PDE6D
M16355	Ras-Independent pathway in NK cell-mediated cytotoxicity	1.561892	2	HLA-A,ITGB1
M8731	Aspirin Blocks Signaling Pathway Involved in Platelet Activation	1.484364	2	F2R,ITGB1
**PantherDB**				
P00034	Integrin signalling pathway	2.208542	7	ACTBL2,COL16A1,COL6A3, COL8A1,FN1,ITGB1,RAC2
P02753	Methionine biosynthesis	1.879927	1	MTR
P00008	Axon guidance mediated by Slit/Robo	1.648607	2	RAC2,SLIT3
P02773	S-adenosylmethionine biosynthesis	1.414162	1	MTR
**Pathway Ontology**				
PW:0000144	ubiquitin/proteasome degradation	2.785164	2	SNCA,UCHL1
PW:0000294	altered ubiquitin/proteasome degradation	2.785164	2	SNCA,UCHL1
PW:0000373	glutathione conjugation	2.243742	2	GSTM1,GSTT1
PW:0000200	the planar cell polarity Wntsignaling	1.80106	2	PRICKLE1,VANGL2
PW:0000018	Parkinson disease	1.746755	2	SNCA,UCHL1
PW:0000495	Gas6 - Axlsignaling axis	1.587404	2	GAS6
PW:0000321	altered Hedgehog signaling	1.587404	1	SMO
PW:0000399	remethylation of homocysteine metabolism - cobalamin dependent	1.587404	1	MTR
PW:0000122	Hedgehog signaling	1.587404	1	SMO
PW:0000302	glomerulonephritis	1.587404	1	ITGB1
**SMPDB**				
SMP00188	GuanidinoacetateMethyltransferase Deficiency (GAMT Deficiency)	1.8799	1	GAMT
SMP00011	Inositol Metabolism	1.8011	2	INPP5J, ITPKA

Table 4 Significant up regulated DEGs from GO analysis across 3 categories including BP, CC and MF.

GO ID	GO TERM	GO Name	logP	Gene Count	Genes
GO:0071466	BP	cellular response to xenobiotic stimulus	10	10	ALDH3A1,CES1,CYP1B1,EPHX1, GSTO2,MGST1,NQO1,UGT1A10, UGT1A7,UGT1A8
GO:0009410	BP	response to xenobiotic stimulus	10	10	ALDH3A1,CES1,CYP1B1,EPHX1, GSTO2,MGST1,NQO1,UGT1A10, UGT1A7,UGT1A8
GO:0006805	BP	xenobiotic metabolic process	10	10	ALDH3A1,CES1,CYP1B1,EPHX1, GSTO2,MGST1,NQO1,UGT1A10 ,UGT1A7,UGT1A8
GO:0052547	BP	regulation of peptidase activity	10	19	A2M,BCL2L10,CASP1,CD109, CDH1,CIDEB,FBLN1,FCMR, GPER1,NOL3,PI3,PYCARD, ROBO1,SERPINB3,SERPINB5, SLPI,SPINK13, SPINK6,SPINT2
GO:0052548	BP	regulation of endopeptidase activity	5.887326	18	A2M,BCL2L10,CASP1,CD109, CDH1,CIDEB,FCMR,GPER1, NOL3,PI3,PYCARD,ROBO1, SERPINB3,SERPINB5,SLPI, SPINK13, SPINK6,SPINT2
GO:0007494	BP	midgut development	4.739146	4	ASS1,CPS1,EDNRB,WNT5A
GO:0009636	BP	response to toxic substance	4.640497	13	ASS1,CDH1,CES1,CPS1,CYP1B1, ENDOG,EPHX1,GSTK1,GSTO2, HYAL3,MAOB,MGST1,NQO1
GO:0051552	BP	flavone metabolic process	4.481589	3	UGT1A10,UGT1A7,UGT1A8
GO:0019731	BP	antibacterial humoral response	4.265238	6	ADM,HIST1H2BC,HIST1H2BJ, HIST1H2BK,HIST2H2BE,SLPI
GO:0009954	BP	proximal/distal pattern formation	4.244012	5	DLX1,HOXD10,HOXD11,IRX2, IRX3
GO:0000786	CC	nucleosome	5.354024	9	HIST1H1C,HIST1H2AC, HIST1H2BC,HIST1H2BD, HIST1H2BJ,HIST1H2BK, HIST2H2AA3,HIST2H2AA4, HIST2H2BE
GO:0044815	CC	DNA packaging complex	5.154995	9	HIST1H1C,HIST1H2AC, HIST1H2BC,HIST1H2BD ,HIST1H2BJ,HIST1H2BK, HIST2H2AA3,HIST2H2AA4, HIST2H2BE
GO:0072559	CC	NLRP3 inflammasome complex	4.054761	3	CASP1,GSDMD,PYCARD
GO:0000788	CC	nuclear nucleosome	3.874916	5	HIST1H2BC,HIST1H2BD , HIST1H2BJ,HIST1H2BK,

					HIST2H2BE
GO:0032993	CC	protein-DNA complex	3.462067	9	HIST1H1C,HIST1H2AC, HIST1H2BC,HIST1H2BD , HIST1H2BJ,HIST1H2BK, HIST2H2AA3,HIST2H2AA4, HIST2H2BE
GO:0061702	CC	inflammasome complex	3.365649	3	CASP1,GSDMD,PYCARD
GO:0072558	CC	NLRP1 inflammasome complex	3.082655	2	CASP1,PYCARD
GO:0097169	CC	AIM2 inflammasome complex	2.691532	2	CASP1,PYCARD
GO:0044454	CC	nuclear chromosome part	2.342884	14	E2F4,HIST1H1C,HIST1H2AC, HIST1H2BC,HIST1H2BD, HIST1H2BJ,HIST1H2BK ,HIST2H2AA3,HIST2H2AA4, HIST2H2BE,HORMAD1,MCM4, RNF212,SYCP2
GO:0000790	CC	nuclear chromatin	2.262669	10	E2F4,HIST1H1C,HIST1H2AC, HIST1H2BC,HIST1H2BD, HIST1H2BJ,HIST1H2BK, HIST2H2AA3,HIST2H2AA4 , HIST2H2BE
GO:0004867	MF	serine-type endopeptidase inhibitor activity	5.604487	9	A2M,CD109,PI3,SERPINB3, SERPINB5,SLPI,SPINK13,SPINK6, SPINT2
GO:0061134	MF	peptidase regulator activity	5.483628	13	A2M,CASP1,CD109,FBLN1,NOL3, PI3,PYCARD,SERPINB3, SERPINB5,SLPI,SPINK13,SPINK6, SPINT2
GO:0004866	MF	endopeptidase inhibitor activity	4.338386	10	A2M,CD109,NOL3,PI3,SERPINB3, SERPINB5,SLPI,SPINK13,SPINK6, SPINT2
GO:0061135	MF	endopeptidase regulator activity	4.211932	10	A2M,CD109,NOL3,PI3,SERPINB3, SERPINB5,SLPI,SPINK13,SPINK6, SPINT2
GO:0030414	MF	peptidase inhibitor activity	4.150696	10	A2M,CD109,NOL3,PI3,SERPINB3, SERPINB5,SLPI,SPINK13,SPINK6, SPINT2
GO:0046982	MF	protein heterodimerization activity	4.149112	19	BCL2L10,CEBPD,DMRTA2, HIST1H2AC,HIST1H2BC, HIST1H2BD,HIST1H2BJ, HIST1H2BK,HIST2H2AA3, HIST2H2AA4,HIST2H2BE,IRAK3, MID2,MLXIPL,NEFH,SYCP2, UGT1A10,UGT1A7,UGT1A8
GO:0001972	MF	retinoic acid binding	3.810235	4	UGT1A10,UGT1A7,UGT1A8,

					UGT2B7
GO:0016723	MF	oxidoreductase activity, oxidizing metal ions, NAD or NADP as acceptor	3.569935	3	MTRR,STEAP1,STEAP4
GO:0016491	MF	oxidoreductase activity	3.49622	21	AKR1C2,AKR1C4,ALDH3A1,CBS, CHDH,CREG1,CYP1B1,FOXRED2, GCDH,GSTK1,GSTO2,HMOX1,HP DL,HTATIP2,IFI30,MAOB,MGST1, MTRR,NQO1,STEAP1,STEAP4
GO:0004857	MF	enzyme inhibitor activity	3.486003	14	A2M,CD109,NOL3,PI3,PRKAR1B, SERPINB3,SERPINB5,SLPI, SPINK13,SPINK6,SPINT2, UGT1A10, UGT1A7,UGT1A8

BP- Biological Process, CC- Cellular Components and MF- Molecular Function

Table 5 Significant down regulated DEGs from GO analysis across 3 categories including BP, CC and MF.

GO ID	GO TERM	GO Name	logP	Gene Count	Genes
GO:0030198	BP	extracellular matrix organization	4.794060318	16	COL16A1,COL6A3,COL8A1,CTGF, EGFLAM,FBN1,FN1,FSCN1,GAS6,IT GB1,JAM3,MFAP2,PXDN,SNCA,TN C,VCAN
GO:0043062	BP	extracellular structure organization	4.779002472	16	COL16A1,COL6A3,COL8A1,CTGF, EGFLAM,FBN1,FN1,FSCN1,GAS6,IT GB1,JAM3,MFAP2,PXDN,SNCA,TN C,VCAN
GO:0031175	BP	neuron projection development	4.767829706	29	ASAP1,BRSK1,CNTNAP1,FARP1,FN 1, GLI2,HLA-A , INPP5J,ITGB1,ITPKA,JAM3,LGALS1 , LRP4,MTR,NBL1,NLGN2,NRP2,SEM A4F,SLIT3,SMO,STMN3,TNC,TRPV 2,UCHL1,VANGL2,VIM,VLDLR,ZM YND8,ZNF280B
GO:0002484	BP	antigen processing and presentation of endogenous peptide antigen via MHC class I via ER pathway	3.770235982	3	ABCB9,HLA-A,HLA-C
GO:0006790	BP	sulfur compound metabolic process	3.579205205	15	ACOT13,EGFLAM,GAMT,GSTM1,G STT1,GSTT2,GSTT2B,MTR,NOX4SL C35D2, SNCA,ST3GAL3,SULT1A2,VANGL2 , VCAN
GO:0048666	BP	neuron development	3.575250765	29	ASAP1,BRSK1,CNTNAP1,FARP1,FN 1, GLI2,HLA-A, INPP5J,ITGB1,ITPKA,JAM3,LGALS1 , LRP4,MTR,NBL1,NLGN2,NRP2,SEM A4F,SLIT3,SMO,STMN3,TNC,TRPV

					2,UCHL1,VANGL2,VIM,VLDLR,ZMYND8,ZNF280B
GO:0030182	BP	neuron differentiation	3.561879295	34	ASAP1,BRSK1,CNTNAP1,FARP1,FN1, GLI2,HLA-A, INPP5J,ITGB1,ITPKA,JAM3,LGALS1, LRP4,MTR,NBL1,NLGN2,NRP2,SEMA4F,SLIT3,SMARCA1,SMO,STMN3, TCF3, TGIF2,TNC,TRPV2,UCHL1,VANGL2,VIM,VLDLR,WNT5B,ZC4H2,ZMYND8, ZNF280B
GO:0031589	BP	cell-substrate adhesion	3.515144761	13	COL16A1,COL8A1,CTGF,EGFLAM, FN1, GAS6,ITGB1,JAM3,LGALS1,RAC2,SGCE,SIRPA,TMEM8B
GO:0030030	BP	cell projection organization	3.421293178	34	ASAP1,BRSK1,CNTNAP1,FARP1,FN1, FSCN1,GLI2,HLA-A, INPP5J,ITGB1,ITPKA,JAM3,LGALS1, LRP4,MTR,NBL1,NECTIN2,NLGN2, NRP2, RAC2,SEMA4F,SLIT3,SMO,STMN3, TMEM107,TNC,TPM1,TRPV2,UCHL1, VANGL2,VIM,VLDLR,ZMYND8,ZNF280B
GO:0061564	BP	axon development	3.397133579	17	BRSK1,FN1,GLI2,ITGB1,JAM3,LRP4, MTR,NRP2,SEMA4F,SLIT3,SMO,TNC, TRPV2,UCHL1,VANGL2,VIM,ZNF280B
GO:0031012	CC	extracellular matrix	10	21	COL16A1,COL6A3,COL8A1,CTGF,ECM1,EFEMP2,EGFLAM,FBN1,FN1, GPC6 , IGFBP7,ITGB1,LGALS1,MFAP2,MFGE8, PXDN,SLIT3,SNCA,TNC,VCAN,WNT5B
GO:0005578	CC	proteinaceous extracellular matrix	10	19	COL16A1,COL6A3,COL8A1,CTGF,ECM1,EFEMP2,EGFLAM,FBN1,FN1, GPC6, ITGB1,LGALS1,MFAP2,PXDN,SLIT3, SNCA,TNC,VCAN,WNT5B
GO:0044432	CC	endoplasmic reticulum part	5.019861528	34	ABCB9,CERCAM,CKAP4,CNIH2, COL16A1,COL6A3,COL8A1,CPT1C, CYP4V2,FAM69B,GAS6,GPSM1, HLA-A,HLA-C, KDELR3,MBOAT1,MOXD1,NFE2L1,NOX4,P4HA2,PLD3,POMT2,RAB18,RCN3,REEP2,RHBDF1,RHBDF2,SCARA3,SEZ6L2,SLC27A5,SORT1,TUS

					C3,UCHL1,WNT5B
GO:0044420	CC	extracellular matrix component	4.807767499	10	COL6A3,COL8A1,EFEMP2,EGFLAM, FBN1,FN1,ITGB1,MFAP2,SNCA,TNC
GO:0097517	CC	contractile actin filament bundle	3.638892609	6	FSCN1,MLPH,MPRIP,NOX4,TPM1, VANGL2
GO:0001725	CC	stress fiber	3.638892609	6	FSCN1,MLPH,MPRIP,NOX4,TPM1, VANGL2
GO:0030425	CC	dendrite	3.579902617	19	ASAP1,CNIH2,CPT1C,FARP1,FSCN1, GNAI2,INPP5J,ITGB1,ITPKA,LRP4, MAGEE1,MLPH,NLGN2,RPS6,SACS, SGCE,SMO,SORT1,ZMYND8
GO:0042175	CC	nuclear outer membrane-endoplasmic reticulum membrane network	3.491291542	27	ABCB9,CKAP4,CNIH2,CPT1C,CYP4V2, FAM69B,GPSM1,HLA-A,HLA-C, KDELR3,MBOAT1,MOXD1,NFE2L1, NOX4,PLD3,POMT2,RAB18,REEP2, RHBDF1,RHBDF2,SCARA3,SEZ6L2,SLC27A5,SNCA,SORT1,TUSC3,UCHL1
GO:0032432	CC	actin filament bundle	3.435008596	6	FSCN1,MLPH,MPRIP,NOX4,TPM1, VANGL2
GO:0036477	CC	somatodendritic compartment	3.399737417	24	ASAP1,CNIH2,CPT1C,EEF1A2,ENO2, FARP1,FSCN1,GNAI2,INPP5J,ITGB1, ITPKA,LRP4,MAGEE1,MLPH,NLGN2, RPS6,SACS,SEZ6L2,SGCE,SHROOM2, SMO,SORT1,UCHL1,ZMYND8
GO:0050839	MF	cell adhesion molecule binding	4.081606647	11	COL16A1,CTGF,DSP,FBN1,FN1,ITGB1, JAM3,MFGE8,NECTIN2,NECTIN3, NLGN2
GO:0004601	MF	peroxidase activity	3.566969085	5	GSTT1,GSTT2,GSTT2B,PRDX5,PXDN
GO:0016684	MF	oxidoreductase activity, acting on peroxide as acceptor	3.477211008	5	GSTT1,GSTT2,GSTT2B,PRDX5,PXDN
GO:0001968	MF	fibronectin binding	3.089125718	4	CTGF,ITGB1,MFAP2,TNC
GO:0004364	MF	glutathione transferase activity	3.039134323	4	GSTM1,GSTT1,GSTT2,GSTT2B
GO:0005178	MF	integrin binding	3.012293979	7	COL16A1,CTGF,FBN1,FN1,ITGB1,JAM3,MFGE8

GO:0005521	MF	lamin binding	2.903011586	3	BNIP3L,IFI27,PPP4C
GO:0016765	MF	transferase activity, transferring alkyl or aryl (other than methyl) groups	2.888457589		
				5	GSTM1,GSTT1,GSTT2,GSTT2B,SEC 14L2
GO:0004602	MF	glutathione peroxidase activity	2.690972913		
				3	GSTT1,GSTT2,GSTT2B
GO:0019838	MF	growth factor binding	2.612556934	7	CTGF,IGFBP7,NRP2,PXDN,RHBDF1 , RHBDF2,SORT1

BP- Biological Process, CC- Cellular Components and MF- Molecular Function

Table 6 Topology table for up and down regulated genes

Regulation	Node	Degree	Betweenness	Stress	Closeness	Clustering Coefficient
Up	NPTX1	8	0.00253	183634	0.30005	0
Up	KLHL35	2	4.73E-04	55790	0.193838	0
Up	NEFH	3	9.46E-04	52466	0.292555	0
Up	ASS1	43	0.01351	1677568	0.318086	0.002439
Up	SLPI	3	4.73E-04	49308	0.187104	0
Up	FBLN1	36	1.10E-02	1279202	0.290325	0.002151
Up	TYMP	9	0.001002	151840	0.307961	0
Up	ARHGEF35	1	0.00E+00	0	0.292474	0
Up	SPINT2	5	6.58E-04	203154	0.30767	0
Up	ASS1	43	0.01351	1677568	0.318086	0.002439
Up	UGT1A10	1	0.00E+00	0	0.216554	0
Up	NOL3	37	8.30E-03	1241228	0.270125	0
Up	LONRF3	2	9.14E-06	1382	0.22282	0
Up	HIST1H2BK	15	1.26E-03	350520	0.308884	0
Up	RASIP1	6	1.89E-03	104924	0.292636	0
Up	OLFM1	4	7.79E-04	388740	0.231684	0
Up	GAL	1	0.00E+00	0	0.227239	0
Up	FOXRED2	7	0.00132	112270	0.29691	0
Up	CD14	33	1.09E-02	656264	0.24757	0.002849
Up	TMPRSS3	4	7.59E-04	48364	0.303452	0
Up	MFSD3	5	1.48E-03	92998	0.292636	0
Up	GSTO2	1	0.00E+00	0	0.292474	0
Up	ISL2	4	9.55E-04	55286	0.301075	0
Up	LRRC8B	1	0.00E+00	0	0.233733	0
Up	HIST1H1C	102	2.80E-02	5941838	0.336839	0.011728
Up	KIF7	5	3.68E-03	260750	0.295396	0.2
Up	OLFM1	4	7.79E-04	388740	0.231684	0

Up	IL17RB	11	2.86E-03	747146	0.216487	0.027778
Up	KCNK1	6	2.19E-04	35572	0.299137	0
Up	MID2	41	1.03E-02	744118	0.308658	0
Up	ANKLE1	2	9.47E-06	10200	0.299052	0
Up	SYBU	5	6.76E-04	146226	0.223941	0
Up	MAFB	21	6.05E-03	1490840	0.232091	0.005848
Up	IRX2	1	0.00E+00	0	1	0
Up	SRPK2	288	7.20E-02	22460310	0.332547	0.002283
Up	RNF212	1	0.00E+00	0	0.292474	0
Up	SEC14L4	1	0.00E+00	0	0.292474	0
Up	EPHX1	7	2.34E-03	145322	0.302779	0
Up	HIST2H2BE	117	3.45E-02	8392118	0.330959	0.002077
Up	HOXD11	1	0.00E+00	0	0.152575	0
Up	OXCT2	2	0.00E+00	0	0	0
Up	NQO1	22	3.17E-03	423314	0.308276	0.016667
Up	SIK1	11	2.82E-03	275892	0.304107	0
Up	ASS1	43	0.01351	1677568	0.318086	0.002439
Up	HIST2H2AA4	30	8.70E-03	1687486	0.313464	0
Up	EVPL	10	1.57E-03	208780	0.306799	0.055556
Up	NEO1	11	3.46E-03	215610	0.292838	0
Up	MAP7	14	3.72E-03	365170	0.320182	0.012821
Up	LHX2	5	9.54E-04	120598	0.197266	0
Up	ZNF541	1	0	0	0.292474	0
Up	HIST1H2AC	7	2.31E-03	304432	0.303932	0
Up	MBNL3	3	4.18E-05	8384	0.292534	0
Up	SIPA1L2	10	5.99E-03	370548	0.299986	0.066667
Up	ENDOG	8	2.18E-03	139324	0.306	0.047619
Up	TFAP2C	91	3.56E-02	4370646	0.321619	0.00112
Up	TACSTD2	7	2.58E-03	182278	0.248794	0.095238
Up	IFI30	2	2.16E-05	8344	0.223763	0
Up	CBS	1	0.00E+00	0	0.23442	0
Up	TAF7L	6	1.27E-03	217488	0.260923	0.066667
Up	HPDL	15	4.38E-03	369550	0.268427	0
Up	HIST2H2AA4	30	8.70E-03	1687486	0.313464	0
Up	CES1	6	1.14E-05	622	0.199651	0
Up	SNTB1	12	2.70E-03	263106	0.295293	0.030303
Up	DPP7	11	2.85E-03	223330	0.302129	0.018182
Up	TMPRSS3	4	7.59E-04	48364	0.303452	0
Up	LHX6	1	0.00E+00	0	0.167326	0
Up	RYR1	18	0.003647	239738	0.305359	0.015152
Up	FAM83H	11	4.14E-03	305822	0.307872	0.109091
Up	WNT5A	6	2.36E-03	131150	0.292676	0
Up	IFNGR1	24	6.17E-03	382206	0.293082	0.019048

Up	SLC7A5	6	1.68E-04	47400	0.307044	0.266667
Up	CIDEB	5	1.00E+00	6	1	0
Up	PI3	5	9.97E-04	152498	0.217434	0
Up	IL20RB	7	6.06E-01	62	0.818182	0.190476
Up	CHDH	5	4.77E-04	191288	0.249469	0
Up	MEST	30	4.38E-03	632746	0.252177	0
Up	ALDH3A1	4	9.50E-04	249746	0.210296	0
Up	KCTD15	9	1.33E-03	653240	0.255114	0
Up	PRKAR1B	20	3.79E-03	359296	0.295025	0
Up	MID2	41	1.03E-02	744118	0.308658	0
Up	NQO1	22	3.17E-03	423314	0.308276	0.016667
Up	MEIOB	1	0.00E+00	0	0.203759	0
Up	MPZL2	4	2.67E-03	176454	0.294532	0
Up	ASS1	43	0.01351	1677568	0.318086	0.002439
Up	EMILIN2	2	1.76E-05	1668	0.219011	0
Up	PC	21	4.34E-03	679930	0.313278	0.004762
Up	NFIC	14	4.52E-03	555014	0.321643	0.010989
Up	HTATIP2	3	5.59E-04	34086	0.292676	0
Up	UGT2B7	1	0.00E+00	0	1	0
Up	MKX	6	7.75E-04	284964	0.250118	0
Up	CDA	2	4.73E-04	26234	0.292514	0
Up	RBM47	2	4.18E-05	19240	0.298841	0
Up	MAP1LC3A	153	3.70E-02	6879644	0.324084	8.19E-04
Up	CREG1	6	7.09E-04	134810	0.23673	0
Up	A2M	169	5.40E-02	7759798	0.304962	0.004625
Up	HYAL3	1	0.00E+00	0	1	0
Up	CPNE8	4	9.94E-04	63072	0.292595	0
Up	PDZD2	8	1.65E-03	529056	0.242487	0.071429
Up	FBLN1	36	1.10E-02	1279202	0.290325	0.002151
Up	SLC7A6	2	0.00E+00	0	0.249278	1
Up	PODXL2	3	0.00E+00	0	1	0
Up	CDH1	168	5.00E-02	12109668	0.331504	0.001696
Up	CASP1	79	1.88E-02	1453856	0.310198	0.019359
Up	HNRNPA3	80	1.85E-02	4091474	0.341767	0.341767
Up	CCDC84	1	0.00E+00	0	1	0
Up	C8orf33	70	1.32E-02	1464996	0.313929	0
Up	DLX3	12	3.85E-03	1538078	0.22113	0
Up	FKBP11	2	5.07E-06	1824	0.229571	0
Up	KCNK1	6	2.19E-04	35572	0.299137	0
Up	SLC3A2	41	2.61E-02	2771314	0.331114	0.017949
Up	TCEA3	1	0.00E+00	0	1	0
Up	UGT2B7	1	0.00E+00	0	1	0
Up	TXNDC11	17	2.77E-03	349440	0.306777	0

Up	AKR1C2	5	0.00E+00	0	0.292474	0
Up	DPP7	11	0.002855	223330	0.302129	0.018182
Up	CPS1	28	5.75E-03	884158	0.317537	0.012308
Up	DDIT4	24	2.56E-03	402954	0.306821	0.010989
Up	MLXIPL	3	4.73E-04	100708	0.220726	0
Up	TRIM7	10	6.25E-04	265380	0.22997	0
Up	IRAK3	31	1.61E-02	959210	0.308636	0.02
Up	CHN1	14	2.76E-03	878684	0.230033	0
Up	EDNRB	9	2.78E-03	192470	0.301784	0
Up	HIST1H2BK	15	1.26E-03	350520	0.308884	0
Up	MAP7	14	3.72E-03	365170	0.320182	0.012821
Up	AKR1C4	2	4.60E-05	10248	0.292514	0
Up	GSTK1	40	0.010165	1569418	0.324084	0.006006
Up	ACSF3	7	0.001429	122084	0.301763	0.066667
Up	ABLIM1	23	0.006167	761716	0.311065	0
Up	PITX1	26	0.009879	3751440	0.24633	0
Up	PDF	1	0	0	0.187803	0
Up	STEAP4	3	1.58E-04	17396	0.292555	0
Up	GSDMD	1	0	0	0.292474	0
Up	FAM43B	1	0	0	0.292474	0
Up	NPW	1	0	0	1	0
Up	ANKLE1	2	9.47E-06	10200	0.299052	0
Up	BCL2L10	7	0.001152	79762	0.293082	0
Up	KRT17	41	0.009499	1354360	0.320935	0.035088
Up	AUTS2	4	9.49E-04	330030	0.22972	0
Up	PIK3C2B	16	0.001872	295394	0.308118	0.030303
Up	WDR11	6	0.00119	91762	0.292717	0
Up	CD7	9	0.003309	334780	0.199246	0
Up	CFD	3	7.42E-04	149586	0.295685	0
Up	PDE9A	4	4.73E-04	25270	0.190915	0
Up	SLC7A6	2	0.00E+00	0	0.249278	1
Up	CCT5	126	0.044179	10033170	0.34534	0.023613
Up	STEAP1	1	0	0	0.292474	0
Up	HIST1H2BC	4	5.35E-04	168706	0.249351	0
Up	PDZD2	8	1.65E-03	529056	0.242487	0.071429
Up	HOXD10	4	0.001431	356758	0.219534	0
Up	SLC47A1	1	0	0	1	0
Up	SERPINB5	17	0.002673	278418	0.302086	0
Up	EFNA1	16	0.003371	927630	0.235872	0.025641
Up	FOXRED2	7	0.00132	112270	0.29691	0
Up	IRAK3	31	0.016114	959210	0.308636	0.02
Up	E2F4	479	0.205549	20592222	0.340666	1.19E-04
Up	SPINK6	1	0	0	0.216554	0

Up	ASS1	43	0.01351	1677568	0.318086	0.002439
Up	S100P	21	0.003357	254344	0.303496	0
Up	IREB2	16	0.003949	302678	0.306177	0
Up	SPINK13	1	0	0	0.292474	0
Up	KRT6A	33	0.005165	972594	0.31948	0.032258
Up	ATP6V1B1	88	0.032117	3026874	0.322921	9.26E-04
Up	PDE3B	6	0.001893	113534	0.295726	0.1
Up	CYP1B1	2	4.73E-04	26234	0.292514	0
Up	SNTB2	24	0.008921	687370	0.309766	0.009524
Up	MPZL1	6	0.001062	71482	0.302454	0
Up	RAB17	5	0.001442	536898	0.19276	0
Up	FAM46A	32	0.00519	421904	0.307804	0
Up	CELSR3	4	1	12	1	0
Up	SYT12	1	0	0	0.292474	0
Up	CBWD3	1	0	0	0.292474	0
Up	IRX3	1	0	0	0.142491	0
Up	MPZL2	4	2.67E-03	176454	0.294532	0
Up	PTGES	5	0	0	0.292474	0
Up	PYCARD	47	0.008292	656866	0.301612	0.058712
Up	SVEP1	9	6.33E-04	108246	0.226569	0
Up	EIF4E3	1	0	0	0.205342	0
Up	CPVL	16	0.009752	765028	0.311478	0
Up	ACSL5	2	2.60E-04	24072	0.292575	0
Up	HENMT1	3	7.20E-05	7628	0.296556	0
Up	PRMT7	18	0.003641	306412	0.301741	0
Up	CEBPD	119	0.046304	5340774	0.313766	0.00177
Up	MGST1	4	0.001298	95348	0.304107	0
Up	MTRR	5	0.001115	73138	0.302454	0
Up	IL13RA2	3	4.73E-04	26234	0.292514	0
Up	GSPT1	36	0.010438	1069760	0.323811	0.017204
Up	THNSL2	1	0	0	1	0
Up	GLB1L2	1	0	0	0.292474	0
Up	HIST1H2BD	18	0.002701	680682	0.313417	0.03268
Up	RBM47	2	4.18E-05	19240	0.298841	0
Up	MRPS30	7	0.00641	487496	0.310357	0.133333
Up	TTLL12	56	0.019125	2780144	0.313069	0.005844
Up	SLC16A8	2	4.73E-04	61574	0.203084	0
Up	KYNU	31	0.011328	575952	0.307939	0
Up	PPL	26	0.003519	380700	0.306	0.013072
Up	MAOB	7	6.22E-04	59916	0.292656	0
Up	HMOX1	17	0.001982	263200	0.307827	0
Up	EFNA1	16	0.003371	927630	0.235872	0.025641
Up	SERPINB3	17	0.005351	511084	0.304282	0

Up	RBM43	1	0	0	0.292474	0
Up	SNRNP48	4	0.001706	124368	0.30343	0
Up	VAV3	23	0.008307	670150	0.306221	0
Up	DLX1	1	0	0	0.198135	0
Up	HIST1H2BD	18	0.002701	680682	0.313417	0.03268
Up	TTC39C	3	9.46E-04	290518	0.220714	0
Up	SALL4	7	0.002334	216012	0.303823	0
Up	DDX19A	14	0.003256	451380	0.325156	0.087912
Up	C22orf39	2	0	0	0.208893	0
Up	MCM4	97	0.027287	3833458	0.332809	0.035284
Up	FRAS1	5	7.48E-04	77152	0.293143	0
Up	ENG	34	0.008202	1307086	0.251712	0.05
Up	PIEZO1	3	9.46E-04	52466	0.292555	0
Up	PLBD1	3	3.01E-04	45980	0.299412	0.333333
Up	SNTB2	24	8.92E-03	687370	0.309766	0.009524
Up	RPL13	127	3.01E-02	6293776	0.348328	0.021589
Up	RPUSD4	8	8.32E-03	633056	0.310563	0.035714
Up	MIB2	16	4.08E-03	266254	0.301612	0.012821
Up	LAMC3	21	7.79E-03	864456	0.299327	0
Up	GCDH	16	8.65E-03	874358	0.304479	0.066667
Up	VAC14	16	3.83E-03	1285316	0.252403	0
Up	ROBO1	8	3.83E-03	205744	0.299667	0
Up	UGT1A7	1	0.00E+00	0	1	0
Up	CPVL	16	0.009752	765028	0.311478	0
Up	NPRL3	6	1.76E-03	188546	0.298946	0
Down	UCHL1	61	0.015169	2258798	0.32743	0.003771
Down	VAT1	19	0.007237	515036	0.323638	0.011696
Down	HLA-C	30	0.00858	797012	0.317873	0.012308
Down	FAM69B	2	5.55E-04	146124	0.248604	0
Down	ECM1	10	0.002251	238282	0.299269	0.027778
Down	COL6A3	2	2.59E-06	416	0.243105	0
Down	SNURF	2	2.33E-05	3116	0.23658	0
Down	SCARA3	4	2.19E-04	16418	0.29705	0
Down	TMEM107	1	0.00E+00	0	0.190348	0
Down	TUSC3	4	5.95E-04	76688	0.309018	0
Down	ZC4H2	5	2.22E-03	1310100	0.175819	0
Down	POMT2	2	1.87E-05	11316	0.233545	0
Down	SSBP4	8	2.65E-03	195422	0.300968	0
Down	VANGL2	4	1.66E-03	100260	0.29661	0
Down	GLI2	19	6.38E-03	1627552	0.255095	0
Down	SNRPN	28	6.88E-03	906314	0.324453	0.005291
Down	TMEM44	2	0.00E+00	0	0.229837	0
Down	IFFO1	8	2.63E-03	239882	0.298452	0

Down	LGALS1	57	1.55E-02	1654992	0.351433	0.021116
Down	TCEAL3	2	1.94E-05	25754	0.300267	0
Down	SIRPA	11	4.07E-03	318340	0.296903	0
Down	CMTM3	6	6.20E-04	103532	0.241573	0
Down	GSPT2	26	7.08E-03	780746	0.321703	0.003953
Down	NUDT11	1	0.00E+00	0	0.296464	0
Down	CCNA1	69	1.59E-02	6292602	0.319253	0
Down	WWC3	1	0.00E+00	0	0.234578	0
Down	SH3BGRL	15	3.62E-03	382642	0.298798	0
Down	TMEM44	2	0.00E+00	0	0.229837	0
Down	PLD3	19	3.60E-03	395068	0.3125	0.008333
Down	COL16A1	2	7.08E-06	1054	0.261251	0
Down	SEC14L2	2	5.55E-04	33422	0.296513	0
Down	PTPRB	40	9.57E-03	833620	0.301749	0.004301
Down	SMO	10	1.20E-03	349232	0.251447	0
Down	VAMP5	4	2.29E-04	23086	0.29661	0
Down	EHBP1L1	10	5.57E-03	459214	0.311447	0
Down	SNRPN	28	0.006875	906314	0.324453	0.005291
Down	VLDLR	15	4.23E-03	293538	0.298501	0
Down	DYRK4	17	1.81E-03	474360	0.283881	0
Down	STMN3	7	1.94E-03	156204	0.298378	0
Down	VIM	268	1.05E-01	10345086	0.400111	0.004648
Down	PELI2	25	9.14E-03	1217118	0.334478	0.019048
Down	DKK3	1	0.00E+00	0	0.203007	0
Down	PARD6G	24	6.16E-03	445938	0.311232	0.026316
Down	DOCK10	2	5.55E-04	33422	0.296513	0
Down	SLC2A12	2	6.94E-05	48758	0.302255	0
Down	BNIP3L	42	1.40E-02	1126694	0.307253	0.002463
Down	SMARCA1	23	8.81E-03	749718	0.322826	0.025974
Down	GPSM3	4	2.50E-05	1554	0.227459	0
Down	MAGEE1	8	4.70E-04	80182	0.308383	0
Down	GAS6	7	1.66E-03	100260	0.29661	0
Down	MOXD1	4	9.35E-05	20142	0.305612	0
Down	RHBDF2	1	0.00E+00	0	0.296464	0
Down	SORT1	30	1.15E-02	656976	0.313342	0.023333
Down	GPR161	1	0.00E+00	0	0.20546	0
Down	NOX4	2	5.55E-04	33422	0.296513	0
Down	GAMT	7	1.62E-03	150496	0.299071	0
Down	HLA-A	44	1.44E-02	1525888	0.318238	0.002845
Down	SPESP1	1	0.00E+00	0	0.211449	0
Down	MECOM	63	1.20E-02	8070650	0.29419	0.006734
Down	CTGF	16	2.66E-03	304640	0.336727	0.111111
Down	MALL	3	5.58E-04	76428	0.198448	0

Down	KLF6	30	1.05E-02	779656	0.312175	0
Down	NFE2L1	24	6.06E-03	549446	0.306391	0
Down	GAS6	7	1.66E-03	100260	0.29661	0
Down	KAZN	4	5.97E-04	38980	0.296561	0
Down	BRSK1	3	7.09E-04	47768	0.296561	0
Down	ABCB9	1	0.00E+00	0	0.296464	0
Down	CERCAM	3	5.75E-04	46866	0.296659	0
Down	RNF144A	14	3.96E-03	577140	0.260026	0
Down	TRPV2	1	0.00E+00	0	0.296464	0
Down	PDLIM3	3	1.45E-05	3048	0.254249	0
Down	F2R	23	7.56E-03	449620	0.311393	0.010526
Down	RAC2	37	9.45E-03	1658346	0.320872	0.008602
Down	SEZ6L2	6	2.24E-03	162720	0.296708	0
Down	DEF6	9	1.50E-03	115402	0.304863	0.035714
Down	TNC	8	1.18E-03	486070	0.323231	0.190476
Down	FSCN1	34	7.61E-03	1169670	0.342615	0.022989
Down	GNAI2	89	3.47E-02	4342230	0.332289	0.006013
Down	IFI27L2	1	0.00E+00	0	0.225849	0
Down	PKN1	65	1.57E-02	1194246	0.33122	0.005797
Down	RNF144A	14	3.96E-03	577140	0.260026	0
Down	FBN1	16	3.09E-03	1041778	0.325068	0.102564
Down	NUPR1	9	1.74E-03	461542	0.263043	0
Down	DSP	72	2.36E-02	2589184	0.358921	0.015865
Down	GSTM1	7	1.34E-03	87992	0.300768	0
Down	TOX2	3	8.98E-05	32024	0.29917	0
Down	GPSM1	21	5.21E-03	597696	0.309628	0.014706
Down	RBPMS2	2	1.33E-05	2496	0.296586	0
Down	MXRA7	1	0.00E+00	0	0.296464	0
Down	NMT1	41	5.93E-03	1665216	0.322653	0
Down	KDELR3	1	0.00E+00	0	0.296464	0
Down	VCAN	34	1.80E-02	1448362	0.302738	0.019763
Down	SIRPA	11	4.07E-03	318340	0.296903	0
Down	TNFRSF25	12	2.55E-03	166716	0.302154	0
Down	ASAP1	47	1.27E-02	1776394	0.310776	0.00135
Down	RCN3	8	2.91E-03	215978	0.297344	0
Down	DKK3	1	0.00E+00	0	0.203007	0
Down	RPS6	197	3.99E-02	11434072	0.373924	0.019684
Down	P4HA2	6	1.68E-03	143088	0.298057	0
Down	C15orf41	2	5.55E-04	33422	0.296513	0
Down	LDOC1	45	1.46E-02	926560	0.3167	0
Down	TRIM68	11	2.79E-03	160418	0.319111	0.018182
Down	FBXL7	7	1.25E-03	220704	0.255638	0
Down	ZNF25	3	6.86E-04	29880	0.29823	0.333333

Down	COL6A3	2	2.59E-06	416	0.243105	0
Down	ALDH7A1	28	6.37E-03	738526	0.321387	0.014245
Down	EFEMP2	47	1.80E-02	10312416	0.270768	0
Down	RPL10	91	1.12E-02	3150450	0.362384	0.042297
Down	SLC2A3	4	8.85E-04	152558	0.309921	0
Down	RPL39L	2	1.11E-04	11986	0.296561	0
Down	ACTBL2	32	5.58E-03	853598	0.325009	0.012097
Down	SGCE	5	6.90E-04	57028	0.305716	0
Down	FN1	755	2.91E-01	213338628	0.407621	0.001123
Down	EEF1A2	54	1.05E-02	1790384	0.33037	0.006275
Down	CYP4V2	3	5.63E-04	65388	0.237515	0
Down	ABTB2	9	1.17E-03	148932	0.31307	0
Down	PRDX5	36	1.87E-02	1327448	0.324512	0
Down	PNPLA7	1	0.00E+00	0	0.296464	0
Down	ECHDC2	4	1.11E-03	151610	0.234639	0
Down	TUT1	29	6.91E-03	835238	0.314792	0.051724
Down	DNALI1	14	1.51E-03	113056	0.26613	0
Down	GNA11	19	4.74E-03	295254	0.307279	0.006536
Down	MLPH	13	4.49E-03	448076	0.30117	0.036364
Down	BCAS4	1	0.00E+00	0	0.296464	0
Down	AFAP1	9	6.37E-04	256016	0.239455	0
Down	HOXB8	2	1.00E+00	2	1	0
Down	EGFLAM	3	6.63E-05	15316	0.300692	0
Down	GRB10	53	1.30E-02	937116	0.318013	0
Down	WNT5B	2	5.55E-04	33422	0.296513	0
Down	TTYH3	4	8.60E-05	45138	0.297565	0
Down	JAM3	9	2.39E-03	163668	0.296757	0.066667
Down	NAV1	10	9.61E-04	99672	0.304502	0
Down	MOCS1	1	0.00E+00	0	0.194718	0
Down	CNTNAP1	8	1.83E-03	174746	0.301094	0
Down	ACOT13	15	5.02E-03	1938832	0.325832	0.015152
Down	PPP2R3B	13	4.11E-03	206754	0.309044	0.166667
Down	TMEM54	7	1.23E-03	426786	0.263119	0
Down	KLF12	4	4.55E-05	9362	0.236937	0
Down	PXDN	3	5.59E-04	100588	0.237077	0
Down	CPT1C	1	0.00E+00	0	0.296464	0
Down	APOBEC3G	14	2.32E-03	194926	0.304065	0.018182
Down	WDFY1	5	1.24E-03	192124	0.277265	0
Down	LRP4	9	2.47E-03	240366	0.305768	0
Down	NUAK1	28	7.57E-03	715316	0.317649	0
Down	NLGN2	6	1.00E+00	30	1	0
Down	SLC27A5	1	0.00E+00	0	0.241557	0
Down	DNAJC12	2	3.50E-05	11186	0.236782	0

Down	NRP2	5	1.67E-03	116176	0.222696	0
Down	TGIF2	3	5.85E-04	162532	0.251887	0
Down	RPL26	59	1.01E-02	2072648	0.354613	0.036364
Down	PRR14	11	0.00118	126382	0.310428	0
Down	H2AFY2	13	1.56E-03	168378	0.31091	0.051282
Down	CKAP4	34	1.01E-02	1209402	0.321961	0.006048
Down	IKBKE	117	3.21E-02	5852150	0.330461	0.004245
Down	SACS	10	6.11E-03	350306	0.321215	0
Down	CERCAM	3	5.75E-04	46866	0.296659	0
Down	RIMKLB	2	5.55E-04	33422	0.296513	0
Down	ZNF586	1	0.00E+00	0	0.296464	0
Down	SEZ6L2	6	2.24E-03	162720	0.296708	0
Down	ZMYND8	27	7.00E-03	976580	0.321674	0.003333
Down	TCF3	187	6.52E-02	18049254	0.34148	0.002068
Down	RAB18	6	6.87E-04	102622	0.307044	0
Down	ITPKA	1	0.00E+00	0	1	0
Down	P4HA2	6	1.68E-03	143088	0.298057	0
Down	SNCA	1	0.00E+00	0	0.285794	0
Down	EIF3H	2	5.77E-05	72830	0.283123	0
Down	MXRA7	1	0.00E+00	0	0.296464	0
Down	MFAP2	1	0.00E+00	0	0.245338	0
Down	PDE6D	1	0.00E+00	0	0.23493	0
Down	ZNF586	1	0.00E+00	0	0.296464	0
Down	TRIP12	1	0.00E+00	0	0.274709	0
Down	RPS3A	6	9.63E-05	128074	0.299817	0.333333
Down	ITGB1	5	2.61E-04	191458	0.296513	0.333333
Down	ALDH7A1	28	6.37E-03	738526	0.321387	0.014245
Down	COL8A1	1	0.00E+00	0	0.213087	0
Down	GAMT	7	1.62E-03	150496	0.299071	0
Down	ZMYND8	27	7.00E-03	976580	0.321674	0.003333
Down	TPM1	2	0.00E+00	0	0.258053	1
Down	DSTYK	1	0.00E+00	0	0.248398	0
Down	TPM1	2	0.00E+00	0	0.258053	1
Down	GAS6	7	1.66E-03	100260	0.29661	0

Table 7. miRNA - target gene interaction table

Regulation	Target Genes	Degree	Top MicroRNA	Regulation	Target Genes	Degree	Top MicroRNA
Up	NPTX1	200	hsa-miR-8485	Down	UCHL1	22	hsa-miR-4455
Up	KLHL35	11	hsa-miR-1281	Down	VAT1	126	hsa-miR-8089
Up	NEFH	49	hsa-miR-4328	Down	HLA-C	8	hsa-miR-9500
Up	ASS1	13	hsa-miR-3175	Down	FAM69B	20	hsa-miR-4292
Up	SLPI	5	hsa-miR-527	Down	ECM1	20	hsa-miR-4779
Up	HS6ST2	98	hsa-miR-5094	Down	COL6A3	24	hsa-miR-4262
Up	ACTR3C	25	hsa-miR-30d-5p	Down	SNURF	43	hsa-miR-3116
Up	FBLN1	54	hsa-miR-4313	Down	SCARA3	66	hsa-miR-4491

Up	TYMP	1	hsa-miR-92a-3p	Down	TMEM107	20	hsa-miR-4530
Up	ARHGEF35	23	hsa-miR-3125	Down	TUSC3	60	hsa-miR-4452
Up	CHCHD10	12	hsa-miR-7977	Down	GSTT2B	20	hsa-miR-3153
Up	SPINT2	18	hsa-miR-4261	Down	SALL2	46	hsa-miR-4448
Up	ASS1	13	hsa-miR-1275	Down	ZC4H2	112	hsa-miR-4531
Up	UGT1A10	16	hsa-miR-4496	Down	POMT2	69	hsa-miR-4430
Up	TMEM238	3	hsa-miR-4264	Down	SSBP4	14	hsa-miR-7150
Up	ADM	133	hsa-miR-4495	Down	KIAA1324	74	hsa-miR-4478
Up	NOL3	15	hsa-miR-4492	Down	IFI27	6	hsa-miR-6802-3p
Up	LONRF3	51	hsa-miR-3668	Down	VANGL2	83	hsa-miR-4656
Up	HIST2H2AA3	47	hsa-miR-8081	Down	GLI2	70	hsa-miR-4271
Up	HIST1H2BK	15	hsa-miR-3910	Down	SNRPN	9	hsa-miR-3118
Up	RASIP1	1	hsa-miR-4713-5p	Down	TMEM44	9	hsa-miR-4487
Up	OLFM1	32	hsa-miR-1184	Down	TNFAIP8L3	48	hsa-miR-1303
Up	GAL	1	hsa-miR-3925-5p	Down	IFFO1	50	hsa-miR-4442
Up	FOXRED2	59	hsa-miR-9500	Down	LGALS1	3	hsa-miR-22-3p
Up	CD14	4	hsa-miR-296-3p	Down	C8orf88	18	hsa-miR-4306
Up	TMPRSS3	36	hsa-miR-4316	Down	TCEAL3	15	hsa-miR-4259
Up	MFSD3	1	hsa-let-7b-5p	Down	SIRPA	125	hsa-miR-6134
Up	GSTO2	4	hsa-miR-4471	Down	CMTM3	34	hsa-miR-4316
Up	ISL2	5	hsa-miR-4441	Down	GSPT2	40	hsa-miR-4429
Up	LRRC8B	109	hsa-miR-4516	Down	NUDT11	41	hsa-miR-4282
Up	HIST1H1C	22	hsa-miR-5693	Down	CCNA1	10	hsa-miR-640
Up	KIF7	13	hsa-miR-4455	Down	WWC3	112	hsa-miR-4775
Up	OLFM1	32	hsa-miR-4296	Down	SH3BGRL	55	hsa-miR-6844
Up	IL17RB	16	hsa-miR-4307	Down	TMEM44	9	hsa-miR-4276
Up	SYCP2	37	hsa-miR-4452	Down	PLD3	27	hsa-miR-4651
Up	KCNK1	45	hsa-miR-4528	Down	COL16A1	20	hsa-miR-4681
Up	MID2	93	hsa-miR-4531	Down	SLC45A3	81	hsa-miR-4323
Up	HIST1H2BJ	72	hsa-miR-5697	Down	SEC14L2	62	hsa-miR-1284
Up	ANKLE1	20	hsa-miR-4651	Down	PTPRB	178	hsa-miR-4490
Up	SYBU	87	hsa-miR-4261	Down	SMO	29	hsa-miR-4516
Up	UGT1A8	15	hsa-miR-8065	Down	VAMP5	3	hsa-miR-4492
Up	MAFB	60	hsa-miR-4303	Down	EHBP1L1	6	hsa-miR-4532
Up	IRX2	76	hsa-miR-891a-3p	Down	MBOAT1	57	hsa-miR-4510
Up	SRPK2	137	hsa-miR-4267	Down	SNRPN	9	hsa-miR-302f
Up	RNF212	50	hsa-miR-4439	Down	VLDLR	116	hsa-miR-4435
Up	ZFR2	63	hsa-miR-4270	Down	REEP2	36	hsa-miR-4498
Up	SEC14L4	59	hsa-miR-4430	Down	DYRK4	16	hsa-miR-4803
Up	EPHX1	10	hsa-miR-4447	Down	STMN3	18	hsa-miR-4472
Up	HIST2H2BE	26	hsa-miR-3196	Down	VIM	30	hsa-miR-4418
Up	HOXD11	16	hsa-miR-4498	Down	PELI2	158	hsa-miR-4310
Up	SLC16A14	80	hsa-miR-4330	Down	DKK3	56	hsa-miR-4641
Up	OXCT2	6	hsa-miR-4739	Down	PARD6G	36	hsa-miR-3651
Up	NQO1	65	hsa-miR-3938	Down	DOCK10	31	hsa-miR-4719
Up	SMIM4	2	hsa-miR-1279	Down	SLC2A12	124	hsa-miR-5696
Up	SIK1	138	hsa-miR-3119	Down	BNIP3L	169	hsa-miR-6071
Up	ASS1	13	hsa-miR-22-3p	Down	SMARCA1	56	hsa-miR-4422
Up	HIST2H2AA4	26	hsa-miR-6745	Down	GPSM3	29	hsa-miR-4518
Up	EVPL	10	hsa-miR-4419a	Down	MAGEE1	28	hsa-miR-4705
Up	NEO1	109	hsa-miR-4719	Down	GAS6	13	hsa-miR-5095
Up	MAP7	86	hsa-miR-3674	Down	GSTT1	16	hsa-miR-4663
Up	LHX2	54	hsa-miR-5688	Down	ZNF280B	133	hsa-miR-4251
Up	ZNF541	23	hsa-miR-4660	Down	MOXD1	67	hsa-miR-4328
Up	KIAA1211L	12	hsa-miR-30c-5p	Down	RHBDF2	19	hsa-miR-2861
Up	HIST1H2AC	7	hsa-miR-221-3p	Down	C1QL4	6	hsa-miR-4293
Up	MBNL3	308	hsa-miR-2392	Down	SORT1	189	hsa-miR-4270
Up	SIPA1L2	90	hsa-miR-1284	Down	GPR161	47	hsa-miR-3646
Up	ENDOG	1	hsa-miR-26b-5p	Down	NOX4	82	hsa-miR-4303
Up	TFAP2C	49	hsa-miR-4514	Down	GAMT	15	hsa-miR-5095
Up	TACSTD2	14	hsa-miR-4279	Down	HLA-A	23	hsa-miR-4533
Up	IFI30	12	hsa-miR-5682	Down	PROSER2	62	hsa-miR-4521
Up	CBS	18	hsa-miR-4291	Down	SPESP1	7	hsa-miR-4643
Up	TAF7L	34	hsa-miR-4266	Down	MECOM	59	hsa-miR-4268
Up	HPDL	22	hsa-miR-4689	Down	CTGF	71	hsa-miR-1305
Up	HORMAD1	35	hsa-miR-2053	Down	MALL	47	hsa-miR-4673
Up	HIST2H2AA4	26	hsa-miR-6822-3p	Down	KLF6	227	hsa-miR-5093
Up	CES1	2	hsa-miR-370-5p	Down	NFE2L1	74	hsa-miR-4294
Up	SNTB1	105	hsa-miR-3919	Down	GAS6	13	hsa-miR-3123
Up	DPP7	1	hsa-miR-6769a-3p	Down	RSAD1	26	hsa-miR-4308

Up	KIAA1211L	12	hsa-miR-561-3p	Down	KAZN	100	hsa-miR-4299
Up	TMPRSS3	36	hsa-miR-571	Down	BRSK1	21	hsa-miR-4651
Up	DMRTA2	29	hsa-miR-1184	Down	PAQR8	68	hsa-miR-6131
Up	KIAA1644	265	hsa-miR-940	Down	ABCB9	73	hsa-miR-8085
Up	LHX6	168	hsa-miR-5093	Down	CERCAM	56	hsa-miR-4512
Up	RYR1	12	hsa-miR-6134	Down	RNF144A	128	hsa-miR-4698
Up	FAM83H	55	hsa-miR-1827	Down	TRPV2	5	hsa-miR-6858-3p
Up	WNT5A	74	hsa-miR-2110	Down	PDLIM3	39	hsa-miR-1275
Up	IFNGR1	9	hsa-miR-3609	Down	F2R	94	hsa-miR-4791
Up	SLC7A5	67	hsa-miR-761	Down	RAC2	30	hsa-miR-4515
Up	CIDEB	19	hsa-miR-6069	Down	SEZ6L2	37	hsa-miR-3943
Up	PI3	5	hsa-miR-1255a	Down	DEF6	9	hsa-miR-4487
Up	JMJD8	51	hsa-miR-4265	Down	TNC	40	hsa-miR-8054
Up	NINJ2	16	hsa-miR-939-3p	Down	INPP5J	38	hsa-miR-497-5p
Up	IL20RB	14	hsa-miR-648	Down	FSCN1	38	hsa-miR-1912
Up	CHDH	57	hsa-miR-661	Down	GNAI2	85	hsa-miR-4441
Up	MEST	55	hsa-miR-3163	Down	PKN1	3	hsa-miR-129-5p
Up	ALDH3A1	11	hsa-miR-8077	Down	RNF144A	128	hsa-miR-2392
Up	KCTD15	117	hsa-miR-429	Down	FBN1	110	hsa-miR-5682
Up	CD163L1	8	hsa-miR-5696	Down	HNRNPDL	91	hsa-miR-1283
Up	PRKAR1B	28	hsa-miR-2278	Down	NUPR1	9	hsa-miR-4455
Up	SLC22A15	133	hsa-miR-944	Down	DSP	31	hsa-miR-4509
Up	MID2	93	hsa-miR-570-3p	Down	GSTM1	8	hsa-miR-4278
Up	NQO1	65	hsa-miR-3938	Down	TOX2	21	hsa-miR-3168
Up	MEIOB	15	hsa-miR-4699-5p	Down	GPSM1	89	hsa-miR-1205
Up	MPZL2	97	hsa-miR-3611	Down	RBPMS2	76	hsa-miR-3660
Up	ASS1	13	hsa-miR-5008-5p	Down	MXRA7	92	hsa-miR-3908
Up	FAM173B	46	hsa-miR-4679	Down	NMT1	61	hsa-miR-2110
Up	EMILIN2	26	hsa-miR-557	Down	KDELR3	43	hsa-miR-4302
Up	PC	11	hsa-miR-6846-3p	Down	VCAN	124	hsa-miR-3671
Up	NFIC	185	hsa-miR-762	Down	SLC29A4	51	hsa-miR-1538
Up	HTATIP2	34	hsa-miR-107	Down	SIRPA	125	hsa-miR-4499
Up	UGT2B7	8	hsa-miR-6128	Down	TNFRSF25	16	hsa-miR-3609
Up	MKX	139	hsa-miR-4656	Down	ASAP1	127	hsa-miR-548aa
Up	CDA	19	hsa-miR-640	Down	RCN3	18	hsa-miR-4443
Up	RBM47	79	hsa-miR-4426	Down	DKK3	56	hsa-miR-5706
Up	MAP1LC3A	30	hsa-miR-140-3p	Down	RPS6	30	hsa-miR-4312
Up	CREG1	48	hsa-miR-320d	Down	P4HA2	32	hsa-miR-5688
Up	A2M	1	hsa-miR-4251	Down	C15orf41	144	hsa-miR-4528
Up	HYAL3	11	hsa-miR-3148	Down	LDOC1	40	hsa-miR-4764-5p
Up	CPNE8	122	hsa-miR-6126	Down	TRIM68	28	hsa-miR-940
Up	PDZD2	149	hsa-miR-3153	Down	FBXL7	117	hsa-miR-302e
Up	FBLN1	54	hsa-miR-1296-3p	Down	ZNF25	85	hsa-miR-629-3p
Up	SLC7A6	122	hsa-miR-378b	Down	COL6A3	24	hsa-miR-877-5p
Up	MFSD12	10	hsa-miR-4710	Down	ALDH7A1	39	hsa-miR-4691-5p
Up	PODXL2	8	hsa-miR-1538	Down	EFEMP2	14	hsa-miR-1827
Up	CDH1	78	hsa-miR-185-5p	Down	RPL10	33	hsa-miR-548m
Up	CASP1	10	hsa-miR-887-5p	Down	SLC2A3	42	hsa-miR-6800-3p
Up	HNRNPA3	227	hsa-miR-548i	Down	RPL39L	1	hsa-miR-4261
Up	CCDC84	8	hsa-miR-3646	Down	ACTBL2	37	hsa-miR-299-5p
Up	C8orf33	20	hsa-miR-2909	Down	SGCE	18	hsa-miR-586
Up	DLX3	96	hsa-miR-765	Down	FN1	72	hsa-miR-217
Up	CD109	81	hsa-miR-30d-3p	Down	ZBTB42	43	hsa-miR-223-3p
Up	FKBP11	22	hsa-miR-4419b	Down	EEF1A2	10	hsa-miR-3180
Up	KCNK1	45	hsa-miR-1283	Down	CYP4V2	77	hsa-miR-2052
Up	SLC3A2	16	hsa-miR-3154	Down	ABTB2	77	hsa-miR-539-3p
Up	TCEA3	3	hsa-miR-3925-3p	Down	GHDC	12	hsa-miR-6077
Up	UGT2B7	8	hsa-miR-4474-5p	Down	PRDX5	2	hsa-miR-3190-5p
Up	TXNDC11	8	hsa-miR-4282	Down	PNPLA7	7	hsa-miR-6132
Up	AKR1C2	41	hsa-miR-298	Down	ECHDC2	16	hsa-miR-4517
Up	DPP7	1	hsa-miR-6769a-3p	Down	TUT1	21	hsa-miR-1301-3p
Up	CPS1	78	hsa-miR-146b-3p	Down	DNALI1	74	hsa-miR-665
Up	DDIT4	70	hsa-miR-548y	Down	GNA11	29	hsa-miR-548z
Up	MLXIPL	15	hsa-miR-3689d	Down	MLPH	44	hsa-miR-892a
Up	TRIM7	26	hsa-miR-759	Down	BCAS4	11	hsa-miR-4638-3p
Up	IRAK3	61	hsa-miR-3670	Down	AFAP1	160	hsa-miR-936
Up	CHN1	25	hsa-miR-548ap-3p	Down	HOXB8	55	hsa-miR-3199
Up	NPEPL1	36	hsa-miR-520h	Down	EGFLAM	57	hsa-miR-214-3p
Up	EDNRB	94	hsa-miR-520e	Down	GRB10	133	hsa-miR-4318
Up	EPGN	35	hsa-miR-548an	Down	MGME1	21	hsa-miR-3680-5p

Up	HIST1H2BK	15	hsa-miR-525-5p	Down	WNT5B	44	hsa-miR-617
Up	MAP7	86	hsa-miR-23c	Down	TTYH3	176	hsa-miR-190a-3p
Up	GPER1	5	hsa-miR-4691-3p	Down	JAM3	55	hsa-miR-3149
Up	AKR1C4	3	hsa-miR-6165	Down	NAV1	153	hsa-miR-548ac
Up	GSTK1	15	hsa-miR-6764-5p	Down	MOCS1	35	hsa-miR-7108-5p
Up	JMJD8	51	hsa-miR-644a	Down	CNTNAP1	110	hsa-miR-3202
Up	ACSF3	19	hsa-miR-4306	Down	GALNT16	111	hsa-miR-762
Up	ABLIM1	69	hsa-miR-3977	Down	ACOT13	50	hsa-miR-4741
Up	PITX1	31	hsa-miR-6735-5p	Down	PPP2R3B	6	hsa-miR-519d-5p
Up	PDF	16	hsa-miR-934	Down	TMEM54	18	hsa-miR-5001-5p
Up	STEAP4	130	hsa-miR-369-3p	Down	KLF12	192	hsa-miR-4753-3p
Up	GSDMD	2	hsa-miR-4463	Down	PXDN	111	hsa-miR-548w
Up	FAM43B	36	hsa-miR-3180	Down	FAM171A1	63	hsa-miR-8052
Up	NPW	2	hsa-miR-4697-5p	Down	CPT1C	1	hsa-miR-4267
Up	ANKLE1	20	hsa-miR-4509	Down	APOBEC3G	7	hsa-miR-6839-5p
Up	BCL2L10	13	hsa-miR-3147	Down	WDFY1	115	hsa-miR-526a
Up	KRT17	6	hsa-miR-486-3p	Down	LRP4	152	hsa-miR-885-5p
Up	AUTS2	121	hsa-miR-548n	Down	NUAK1	114	hsa-miR-548s
Up	PIK3C2B	73	hsa-miR-650	Down	KCNK6	18	hsa-miR-4261
Up	WDR11	23	hsa-miR-590-3p	Down	NLGN2	83	hsa-miR-6763-5p
Up	CD7	13	hsa-miR-3619-5p	Down	SLC27A5	4	hsa-miR-619-5p
Up	CFD	8	hsa-miR-1224-3p	Down	DNAJC12	24	hsa-miR-4788
Up	PDE9A	3	hsa-miR-4276	Down	NRP2	129	hsa-miR-297
Up	SLC7A6	122	hsa-miR-4288	Down	TMSB15B	9	hsa-miR-548g-3p
Up	CCT5	20	hsa-miR-4716-3p	Down	TGIF2	109	hsa-miR-4436a
Up	STEAP1	58	hsa-miR-7-2-3p	Down	RPL26	8	hsa-miR-100-5p
Up	HIST1H2BC	8	hsa-miR-320a	Down	PRR14	3	hsa-miR-661
Up	PDZD2	149	hsa-miR-607	Down	CNIH2	73	hsa-miR-1913
Up	HOXD10	39	hsa-miR-8080	Down	H2AFY2	24	hsa-miR-4717-3p
Up	SLC47A1	29	hsa-miR-3115	Down	CKAP4	72	hsa-miR-5189-5p
Up	SERPINB5	59	hsa-miR-4477a	Down	IKBKE	28	hsa-miR-4419b
Up	EFNA1	49	hsa-miR-200c-3p	Down	SACS	90	hsa-miR-548y
Up	FOXRED2	59	hsa-miR-6733-5p	Down	CERCAM	56	hsa-miR-6860
Up	IRAK3	61	hsa-miR-3689c	Down	RIMKLB	242	hsa-miR-495-3p
Up	E2F4	28	hsa-miR-1227-3p	Down	ZNF586	47	hsa-miR-4782-5p
Up	SPINK6	5	hsa-miR-1226-3p	Down	SEZ6L2	37	hsa-miR-137
Up	ASS1	13	hsa-miR-6822-5p	Down	ZMYND8	131	hsa-miR-875-3p
Up	S100P	1	hsa-miR-4257	Down	TCF3	40	hsa-miR-4419a
Up	IREB2	86	hsa-miR-5692a	Down	RAB18	138	hsa-miR-548ax
Up	SPINK13	4	hsa-miR-375	Down	ITPKA	11	hsa-miR-378g
Up	KRT6A	18	hsa-miR-544a	Down	MIER2	64	hsa-miR-5196-5p
Up	ATP6V1B1	9	hsa-miR-935	Down	P4HA2	32	hsa-miR-495-3p
Up	PDE3B	114	hsa-miR-1200	Down	SNCA	97	hsa-miR-612
Up	CYP1B1	55	hsa-miR-585-5p	Down	GPC6	233	hsa-miR-544a
Up	SNTB2	98	hsa-miR-144-3p	Down	SHROOM2	103	hsa-miR-29b-3p
Up	MPZL1	112	hsa-miR-30c-2-3p	Down	EIF3H	5	hsa-miR-6820-3p
Up	RAB17	15	hsa-miR-217	Down	TMEM8B	35	hsa-miR-4668-5p
Up	FAM46A	197	hsa-miR-599	Down	ABCG4	193	hsa-miR-329-5p
Up	CELSR3	136	hsa-miR-802	Down	IGFBP7	22	hsa-miR-3686
Up	SYT12	40	hsa-miR-1913	Down	MXRA7	92	hsa-miR-6126
Up	CBWD3	16	hsa-miR-222-3p	Down	MFAP2	31	hsa-miR-608
Up	IRX3	15	hsa-miR-3941	Down	PDE6D	48	hsa-miR-765
Up	MPZL2	97	hsa-miR-8070	Down	ZNF586	47	hsa-miR-939-3p
Up	PTGES	32	hsa-miR-149-3p	Down	CDYL2	48	hsa-miR-491-5p
Up	PYCARD	5	hsa-miR-10b-3p	Down	SLC17A9	36	hsa-miR-484
Up	SVEP1	50	hsa-miR-4700-5p	Down	TRIP12	129	hsa-miR-5703
Up	EIF4E3	113	hsa-miR-548al	Down	RPS3A	20	hsa-miR-4730
Up	CPVL	2	hsa-miR-2117	Down	PPP4C	20	hsa-miR-1296-3p
Up	ACSL5	36	hsa-miR-134-3p	Down	MTMR11	33	hsa-miR-571
Up	HENMT1	14	hsa-miR-548ah-3	Down	MANBA	20	hsa-miR-320d
Up	PRMT7	23	hsa-miR-3943	Down	WDR54	4	hsa-miR-363-5p
Up	CEBPD	7	hsa-miR-885-5p	Down	SLIT3	62	hsa-miR-7515
Up	MGST1	52	hsa-miR-548u	Down	NPR2	16	hsa-miR-634
Up	MTRR	49	hsa-miR-421	Down	ITGB1	64	hsa-miR-7-2-3p
Up	IL13RA2	4	hsa-miR-937-5p	Down	TMSB15A	13	hsa-miR-8060
Up	GSPT1	174	hsa-miR-3150a-3p	Down	STX8	6	hsa-miR-625-5p
Up	THNSL2	8	hsa-miR-4497	Down	ALDH7A1	39	hsa-miR-3617-3p
Up	GLB1L2	18	hsa-miR-4319	Down	COL8A1	57	hsa-miR-6079
Up	HIST1H2BD	19	hsa-miR-888-5p	Down	NLRP2	10	hsa-miR-1914-3p
Up	RBM47	79	hsa-miR-1283	Down	VPS37D	9	hsa-miR-211-3p

Up	MRPS30	16	hsa-miR-563	Down	PLEKHG2	87	hsa-miR-1268b
Up	TTLL12	33	hsa-miR-6741-5p	Down	TMEM47	203	hsa-miR-4666b
Up	SLC16A8	4	hsa-miR-3918	Down	MRPL33	7	hsa-miR-6740-3p
Up	KYNU	15	hsa-miR-429	Down	NBL1	20	hsa-miR-6791-5p
Up	PPL	27	hsa-miR-4303	Down	ENO2	41	hsa-miR-548q
Up	MAOB	38	hsa-miR-5197-3p	Down	ECHDC3	10	hsa-miR-5787
Up	HMOX1	26	hsa-miR-5003-5p	Down	SEMA4F	122	hsa-miR-2909
Up	EFNA1	49	hsa-miR-1291	Down	DYSF	18	hsa-miR-1228-5p
Up	SERPINB3	19	hsa-miR-548k	Down	LRRC49	9	hsa-miR-6837-3p
Up	RBM43	56	hsa-miR-515-5p	Down	PRICKLE1	82	hsa-miR-2681-5p
Up	SNRNP48	54	hsa-miR-877-3p	Down	DENND2A	6	hsa-miR-577
Up	VAV3	106	hsa-miR-204-3p	Down	MFGE8	42	hsa-miR-326
Up	MANEAL	33	hsa-miR-3184-3p	Down	ZSCAN18	10	hsa-miR-6727-5p
Up	DLX1	55	hsa-miR-4703-5p	Down	C15orf52	58	hsa-miR-4291
Up	HIST1H2BD	19	hsa-miR-548ax	Down	MPRIP	230	hsa-miR-548ba
Up	TTC39C	112	hsa-miR-7157-3p	Down	TMEM8A	17	hsa-miR-4728-5p
Up	SALL4	23	hsa-miR-30a-5p	Down	SLC35D2	14	hsa-miR-5002-3p
Up	DDX19A	44	hsa-miR-507	Down	GAMT	15	hsa-miR-504-3p
Up	C22orf39	47	hsa-miR-5691	Down	GSTT2	20	hsa-miR-520h
Up	MCM4	29	hsa-miR-373-5p	Down	MTR	63	hsa-miR-96-5p
Up	FAM217B	53	hsa-miR-520d-5p	Down	ZMYND8	131	hsa-miR-378j
Up	FRAS1	155	hsa-miR-622	Down	RHBDF1	12	hsa-miR-588
Up	ENG	25	hsa-miR-4756-5p	Down	FARP1	98	hsa-miR-425-5p
Up	PIEZO1	5	hsa-miR-101-3p	Down	PIK3IP1	69	hsa-miR-548x-3p
Up	PLBD1	7	hsa-miR-33b-3p	Down	ST3GAL3	26	hsa-miR-7977
Up	SNTB2	98	hsa-miR-5008-3p	Down	HSD11B1L	18	hsa-miR-615-5p
Up	RPL13	31	hsa-miR-3121-3p	Down	TPM1	80	hsa-miR-548n
Up	RPUSD4	15	hsa-miR-196a-3p	Down	DSTYK	105	hsa-miR-143-3p
Up	MIB2	3	hsa-miR-3178	Down	TMEM136	60	hsa-miR-3135a
Up	LAMC3	59	hsa-miR-1915-5p	Down	THG1L	6	hsa-miR-302a-5p
Up	GCDH	16	hsa-miR-6828-3p	Down	YIF1B	17	hsa-miR-663a
Up	VAC14	11	hsa-miR-4664-5p	Down	DNAJC22	12	hsa-miR-3122
Up	ROBO1	105	hsa-miR-548z	Down	TRAF5	49	hsa-miR-18a-5p
Up	UGT1A7	16	hsa-miR-4786-3p	Down	ZSCAN16	5	hsa-miR-133b
Up	CPVL	2	hsa-miR-202-5p	Down	LOC81691	6	hsa-miR-183-5p
Up	NPRL3	24	hsa-miR-548at-3p	Down	ASGR1	4	hsa-miR-361-3p
				Down	TPM1	80	hsa-miR-548ak
				Down	GAS6	13	hsa-miR-874-5p
				Down	CHURC1	55	hsa-miR-4700-3p

Degree – No of miRNA interact with target gene. We selected any one miRNA in network. miRNA- microRNA.

Table 8 TF - target gene interaction table

Regulation	TF	Degree	Target Gene	Regulation	TF	Degree	Target Gene
Up	GLI1	1	HS6ST2	Down	TCF21	3	PPP4C
Up	E2F7	1	MCM4	Down	ZNF652	1	PNPLA7
Up	TCF21	2	LHX6	Down	ZNF263	3	KIAA1324
Up	ZNF652	2	ROBO1	Down	IKZF1	3	SNCA
Up	ZNF263	4	SLC3A2	Down	ZNF322	1	SMO
Up	IKZF1	1	LHX2	Down	PRDM16	1	MTR
Up	ZNF322	1	SALL4	Down	CHD7	3	RPL10
Up	PRDM16	3	CHN1	Down	SALL1	2	TRIP12
Up	CHD7	1	STEAP1	Down	NR4A2	6	RPL26
Up	NR4A2	2	KCTD15	Down	CDKN2AIP	4	TPM1
Up	CDKN2AIP	2	SRPK2	Down	THRA	3	TMEM54
Up	THRA	2	MAFB	Down	HOXD13	2	FBN1
Up	HOXD13	2	MPZL1	Down	FOXM1	2	ZMYND8
Up	FOXM1	2	SNTB1	Down	HTT	6	JAM3
Up	HTT	7	ADM	Down	ETS2	3	GPC6
Up	ETS2	1	SNRNP48	Down	GBX2	2	SNRPN
Up	GBX2	4	SLC22A15	Down	DROSHA	16	DYSF
Up	DROSHA	8	HIST1H1C	Down	FOXO1	3	NRP2
Up	FOXO1	6	TTC39C	Down	RARG	9	PKN1
Up	RARG	7	PPL	Down	RCOR2	10	SLC17A9
Up	RCOR2	5	FBLN1	Down	HIF1A	9	DSP
Up	HIF1A	7	CELSR3	Down	ESR2	9	GRB10
Up	ESR2	19	SYBU	Down	STAT6	7	WWC3
Up	STAT6	12	VAV3	Down	NR1H3	13	DEF6
Up	NR1H3	16	FAM46A	Down	NOTCH1	2	RNF144A

Up	NOTCH1	3	GSPT1	Down	SREBF1	7	FN1
Up	SREBF1	4	IRAK3	Down	THAP11	9	TUT1
Up	THAP11	4	DDX19A	Down	HCFC1	2	NMT1
Up	HCFC1	4	CCT5	Down	CLOCK	3	KDELR3
Up	CLOCK	9	DDIT4	Down	NFIB	9	LGALS1
Up	NFIB	6	RBM47	Down	IRF8	12	TCF3
Up	IRF8	12	ASS1	Down	IRF1	2	MLPH
Up	IRF1	9	UGT1A10	Down	CRX	5	REEP2
Up	CRX	6	NEFH	Down	DNAJC2	14	CDYL2
Up	DNAJC2	16	NQO1	Down	PAX6	20	SLC2A3
Up	PAX6	16	MRPS30	Down	STAT1	7	KLF6
Up	STAT1	10	OLFM1	Down	RBPJ	32	COL6A3
Up	RBPJ	17	PIK3C2B	Down	ELF1	12	SLC2A12
Up	ELF1	16	ANKLE1	Down	PDX1	9	IFFO1
Up	PDX1	5	PC	Down	PADI4	6	NFE2L1
Up	PADI4	14	TMPRSS3	Down	KDM6A	1	VLDLR
Up	KDM6A	5	PRMT7	Down	DCP1A	13	RAB18
Up	DCP1A	8	C8orf33	Down	TFEB	6	YIF1B
Up	TFEB	13	HMOX1	Down	ASXL1	9	MTMR11
Up	ASXL1	3	SLC3A2	Down	TCF7L2	8	ASAP1
Up	TCF7L2	8	EPHX1	Down	NR1I2	12	ST3GAL3
Up	NR1I2	17	FRAS1	Down	GATA3	12	RAC2
Up	GATA3	8	HIST1H2BC	Down	PRDM5	13	SGCE
Up	PRDM5	16	MEST	Down	SREBF2	15	BNIP3L
Up	SREBF2	14	ZFR2	Down	CEBPB	18	CHURC1
Up	CEBPB	22	IL20RB	Down	CEBPA	1	MTR
Up	CEBPA	10	JMJD8	Down	CDX2	11	HLA-A
Up	CDX2	13	A2M	Down	TAF7L	7	NUPR1
Up	BCL3	9	NFIC	Down	BCL3	9	CMTM3
Up	SMAD1	9	HIST1H2BJ	Down	SMAD1	12	F2R
Up	ZIC3	8	CD109	Down	ZIC3	6	VIM
Up	NR0B1	24	HPDL	Down	NR0B1	33	C15ORF41
Up	NACC1	15	MAP7	Down	NACC1	15	FSCN1
Up	SOX11	12	SVEP1	Down	SOX11	15	CCNA1
Up	STAT5A	21	PYCARD	Down	STAT5A	16	COL16A1
Up	TBP	10	HIST1H2BK	Down	TBP	14	DNAJC12
Up	HSF1	11	TRIM7	Down	HSF1	16	EIF3H
Up	BACH1	29	RAB17	Down	BACH1	19	APOBEC3G
Up	EWSR1	12	CPVL	Down	EWSR1	27	ABTB2
Up	ELF5	12	SLC16A14	Down	ELF5	14	BCAS4
Up	RELA	22	ALDH3A1	Down	RELA	24	DOCK10
Up	NUCKS1	9	DLX1	Down	NUCKS1	5	ITGB1
Up	RCOR1	16	TAF7L	Down	RCOR1	15	FBXL7
Up	POU3F2	14	MKX	Down	POU3F2	19	H2AFY2
Up	MEIS1	9	HMOX1	Down	MEIS1	22	CKAP4
Up	LYL1	4	FBLN1	Down	LYL1	12	ABCB9
Up	LMO2	16	FAM46A	Down	LMO2	23	GNA11
Up	MYB	14	IFNGR1	Down	MYB	19	LOC81691
Up	SIN3A	11	FOXRED2	Down	SIN3A	23	COL8A1
Up	PBX1	27	ACSL5	Down	PBX1	25	ECM1
Up	JUN	17	AUTS2	Down	JUN	15	FARP1
Up	TBX3	19	SALL4	Down	TBX3	14	ECHDC2
Up	CTNNB1	16	CBWD3	Down	CTNNB1	17	MALL
Up	TFAP2A	20	GAL	Down	TFAP2A	28	LDOC1
Up	ESRRB	23	LAMC3	Down	ESRRB	23	ACOT13
Up	SUZ12	92	KIAA1644	Down	SUZ12	95	C15ORF52
Up	MTF2	62	CREG1	Down	MTF2	61	C1QL4
Up	JARID2	38	DMRTA2	Down	JARID2	26	HOXB8
Up	RNF2	42	HIST2H2BE	Down	RNF2	33	EGFLAM
Up	EZH2	43	FAM83H	Down	EZH2	33	ABCG4
Up	PHC1	24	HOXD11	Down	PHC1	12	PELI2
Up	EED	27	DLX3	Down	EED	10	EEF1A2
Up	BMI1	34	HOXD10	Down	BMI1	16	SLC35D2
Up	RAD21	28	EVPL	Down	RAD21	38	RCN3
Up	CTCF	20	EMILIN2	Down	CTCF	20	RPS3A
Up	TCF7	18	HYAL3	Down	TCF7	20	GNAI2
Up	FOXP2	22	ENG	Down	FOXP2	29	SPESP1
Up	TBX5	23	DPP7	Down	TBX5	31	WDR54
Up	SRF	10	DDX19A	Down	SRF	13	STX8
Up	MEF2A	12	NINJ2	Down	MEF2A	15	TMEM107

Up	GATA4	22	SNTB2	Down	GATA4	28	FAM171A1
Up	STAT4	16	CCDC84	Down	STAT4	19	VAT1
Up	MECOM	13	HTATIP2	Down	ZFP42	12	TRPV2
Up	ZFP42	13	GSTO2	Down	YY1	28	ALDH7A1
Up	YY1	19	S100P	Down	CHD1	7	PRR14
Up	CHD1	6	GCDH	Down	MYBL2	21	VPS37D
Up	MYBL2	25	HIST1H2AC	Down	HOXB4	19	LRP4
Up	HOXB4	19	LRRC8B	Down	CCND1	28	CMTM3
Up	CCND1	19	FKBP11	Down	SCLY	26	TOX2
Up	SCLY	12	CD14	Down	CUX1	31	HLA-C
Up	CUX1	30	HCG4	Down	RUNX2	47	MOXD1
Up	RUNX2	38	ENDOG	Down	NR3C1	29	TNC
Up	NR3C1	29	BCL2L10	Down	SMAD3	40	MANBA
Up	SMAD3	44	AKR1C2	Down	SMAD2	23	MXRA7
Up	SMAD2	37	CASP1	Down	ATF3	29	PRICKLE1
Up	ATF3	28	ISL2	Down	TFCP2L1	26	SHROOM2
Up	TFCP2L1	16	MANEAL	Down	KLF1	27	SSBP4
Up	KLF1	26	NPRL3	Down	PRDM14	37	GLI2
Up	PRDM14	24	RYR1	Down	TEAD4	28	PLEKHG2
Up	TEAD4	26	SNRNP48	Down	DMRT1	41	CPT1C
Up	DMRT1	28	PLBD1	Down	ZNF281	41	TMEM8A
Up	ZNF281	33	CBS	Down	WT1	39	P4HA2
Up	WT1	29	IRX3	Down	SOX17	25	ZMYND8
Up	SOX17	20	CDA	Down	SALL4	37	SALL2
Up	YAP1	27	SIK1	Down	YAP1	39	KLF12
Up	SMARCA4	36	CYP1B1	Down	SMARCA4	50	MOCS1
Up	OLIG2	17	MAOB	Down	OLIG2	21	PARD6G
Up	ESR1	33	ABLIM1	Down	ESR1	45	SH3BGRL
Up	EP300	27	TYMP	Down	EP300	44	NFE2L1
Up	FOXO3	28	MTRR	Down	FOXO3	24	THG1L
Up	SRY	46	CD7	Down	SRY	62	RHBDF2
Up	SOX9	45	CHDH	Down	SOX9	47	VANGL2
Up	TRIM28	63	HENMT1	Down	TRIM28	54	TMEM47
Up	CNOT3	27	PITX1	Down	CNOT3	16	ABTB2
Up	TAL1	35	SLC7A6	Down	TAL1	50	GPSM3
Up	GFI1B	20	VAC14	Down	GFI1B	19	SIRPA
Up	ERG	46	FAM43B	Down	ERG	33	ZSCAN18
Up	MYCN	22	SLC7A5	Down	MYCN	27	TRIP12
Up	TET1	48	ATP6V1B1	Down	TET1	58	ZNF25
Up	PPARD	50	KYNU	Down	PPARD	56	TRIM68
Up	FOXP1	37	ARHGEF35	Down	FOXP1	60	RPS6
Up	EOMES	40	KCNK1	Down	TFAP2C	51	RAB18
Up	ASH2L	33	EDNRB	Down	EOMES	43	MFGE8
Up	SETDB1	57	EFNA1	Down	ASH2L	42	PDE6D
Up	REST	51	CD163L1	Down	SETDB1	62	ECHDC3
Up	SIN3B	53	PRKAR1B	Down	REST	51	TUSC3
Up	RCOR3	43	MAP1LC3A	Down	SIN3B	58	MRPL33
Up	ZFX	31	MPZL2	Down	RCOR3	42	CHURC1
Up	KDM5B	38	WNT5A	Down	E2F4	39	TMEM136
Up	GATA2	48	NPW	Down	ZFX	31	IFFO1
Up	GATA1	52	SLC47A1	Down	KDM5B	28	C15ORF41
Up	HNF4A	100	FAM217B	Down	GATA2	64	SLIT3
Up	FOXA2	45	TMPRSS3	Down	GATA1	57	SLC45A3
Up	PPARG	48	ACSF3	Down	HNF4A	97	DNAJC22
Up	TP53	79	LONRF3	Down	FOXA2	46	SLC17A9
Up	MITF	65	LOC284023	Down	PPARG	59	LOC81691
Up	EGR1	95	MFSD3	Down	TP53	92	ITPKA
Up	TCF3	43	TCEA3	Down	MITF	83	FAM69B
Up	SOX2	124	KRT6A	Down	EGR1	110	ZNF280B
Up	POU5F1	67	MIB2	Down	SOX2	120	TMSB15A
Up	NANOG	76	PDF	Down	POU5F1	77	PKN1
Up	KLF4	65	TXNDC11	Down	NANOG	95	RPL10
Up	SPI1	91	LOC389641	Down	KLF4	62	ECHDC2
Up	MYC	109	MRPS30	Down	SPI1	99	GSTT2
Up	RUNX1	83	KIF7	Down	MYC	105	BRSK1
Up	FLI1	57	PRMT7	Down	RUNX1	89	RPL39L
Up	E2F1	61	CHCHD10	Down	FLI1	103	PPP2R3B
Up	CREM	54	IREB2	Down	E2F1	71	DNAJC12
Up	CREB1	37	CPNE8	Down	CREM	60	FN1
Up	TP63	82	UGT2B7	Down	CREB1	55	NAV1

Up	SMAD4	63	UGT1A8	Down	TP63	103	IFI27L2
Up	TCF4	42	PI3	Down	SMAD4	66	GPC6
Up	STAT3	84	ZNF541	Down	TCF4	53	DOCK10
Up	AR	76	SLC16A14	Down	STAT3	84	FBN1
Up	PHF8	23	C8orf33	Down	AR	99	RIMKLB
Up	DACH1	18	HIST1H2BD	Down	PHF8	6	ALDH7A1
Up	ELK1	15	MRPS30	Down	DACH1	16	POMT2
Up	XRN2	14	DDX19A	Down	ELK1	26	RPL26
Up	TTF2	21	SNHG9	Down	XRN2	15	ZMYND8
Up	FOXP3	14	E2F4	Down	TTF2	20	STX8
Up	VDR	29	RBM47	Down	FOXP3	16	KLF6
Up	HOXC9	15	CELSR3	Down	VDR	30	RAC2
Up	KDM5A	12	RPUSD4	Down	HOXC9	25	UCHL1
Up	ETS1	4	GSPT1	Down	KDM5A	13	LRRC49
Up	AP1S2	3	RASIP1	Down	ETS1	15	DSTYK
Up	NFE2L2	24	ABLIM1	Down	AP1S2	5	TUT1
Up	PAX3	15	ROBO1	Down	NFE2L2	31	GPR161
Up	ZNF217	30	SYBU	Down	CEBPD	8	CTGF
Up	ARNT	20	SLC7A5	Down	PAX3	14	WNT5B
Up	AHR	14	CYP1B1	Down	ZNF217	19	TPM1
Up	KLF5	1	TFAP2C	Down	ARNT	14	ASAP1
Up	KLF2	1	TFAP2C	Down	AHR	9	PNPLA7
				Down	KLF5	3	SLC2A3
				Down	KLF2	3	DENND2A

Degree – No of target gene interact with TF.

We selected any one TF in network. TF -transcription factors
